# Environmental stochasticity reduces human planning effort

**DOI:** 10.1038/s41467-026-78023-9

**Published:** 2026-09-28

**Authors:** Jordan Lei, Jeroen Olieslagers, Nastaran Arfaei, Daisy Xinlei Lin, Wei Ji Ma

**Affiliations:** 1 Center for Neural Science, New York University, New York, NY USA; 2 Department of Psychology, New York University, New York, NY USA

**Keywords:** Human behaviour, Human behaviour

## Abstract

The world is stochastic, making planning difficult. Despite the ubiquity of stochasticity in real-world environments, it remains an open question how people effectively balance the cognitive costs of planning against its potential benefits when faced with stochasticity. To study this, we designed a planning task where participants face one of three forms of stochasticity commonly encountered in the real world: reliability, volatility, and controllability. We find a robust pattern across all three manipulations: as stochasticity increases, people reduce their planning effort as measured by first-choice response times. To understand the processes underlying this effect, we developed several computational cognitive models to account for participants’ choices. We find that rather than calculating expected values optimally, people choose a simpler strategy, acting as if the world were deterministic, a phenomenon known as determinizing. Consistent with our response time findings, we find that people reduce their effort by decreasing their sensitivity to values with increasing stochasticity, a signature of policy compression. Our work highlights the often overlooked role of stochasticity in human planning and its impact on planning strategy and effort. Moreover, it reveals the limitations of studying stochasticity solely through single-shot decisions.

## Abstract (web summary)

The real world is stochastic, making it difficult to know when planning is worthwhile. Here the authors show that as stochasticity increases, people spend less effort planning and adopt simpler, less value-sensitive decision policies.

## Introduction

Planning is a cognitive process that involves thinking multiple steps ahead to achieve a goal. In the real world, planning is complicated by the fact that the environment is often stochastic. Random factors out of our control, such as shifting climate patterns^1,2^ or unexpected disease pandemics^3^ can ruin even the best-laid plans.

Stochasticity in planning can manifest in various forms. Imagine you are planning a trip to an unfamiliar city and want to dine at the best restaurants, but your list of recommendations is outdated. Some restaurants may have improved or declined in quality since they were last rated. Here, the stochasticity lies in the reward. Reward stochasticity can take on different characteristics. In some cases, as with outdated recommendations or imperfect information, the environment can be described as *unreliable*. Developmental studies have suggested that reliability of future information modulates cognitive effort. For example, children and adults become more proactively engaged when information about future tasks is reliable than when it is unreliable^4,5^. In other cases, such as with stocks^6–8^ or weather patterns^9,10^, the reward may be known but is subject to unpredictable fluctuations over time. This dynamic form of stochasticity, which involves temporal fluctuations in reward, is sometimes described as *volatility*^11–16^. Existing work on volatility has focused on how agents learn in volatile environments^11–15^, but does not speak to its potential effects on planning effort.

Another form of stochasticity arises in the transition structure of the environment. Imagine you’re trying to get to your favorite restaurant by navigating a complex subway system. However, due to ongoing maintenance, some trains are unpredictably rerouted. Even if you plan your route carefully, a train might unexpectedly take you to a different station. In these environments, the connection between an agent’s choices and the resulting future states is disrupted. This reduced *controllability* adds a unique dimension to decision-making. For example, people engage in more counterfactual reasoning when faced with more controllable scenarios compared to less controllable ones^17–19^. However, it remains an open question how people change their effort in uncontrollable environments.

Despite the prevalence of stochasticity in the real world, most planning studies have either relied on deterministic tasks—such as the Tower of London^20–24^, chess^25–27^, Four-in-a-Row^28^—or used stochasticity merely as an incidental tool^29–32^. One critical question in the study of planning is how planners allocate cognitive resources to plan ahead^33–35^. This becomes especially relevant in stochastic environments, where determining the best plan may be cognitively demanding or even computationally infeasible. Much of the existing work on how stochasticity affects planning has focused on risk preferences in planning rather than on how individuals allocate cognitive resources in stochastic environments. For example, studies show that people develop effective contingency plans to overcome spatial stochasticity^36,37^, and individuals tend to choose safer routes over riskier ones, even when shorter paths are available^36,37^. While these studies highlight the importance of stochasticity in planning, they fail to address how people manage the tradeoff between the rewards and costs of planning in stochastic environments.

A growing body of work suggests that people weigh cognitive costs against potential benefits when making decisions^38–40^. This idea is formalized in the framework of *resource rationality*^39^, which describes how to optimally manage this tradeoff. For instance, when deciding whether to plan ahead or act on prior knowledge, people evaluate the benefits of exploiting existing knowledge against the cost of getting new information^41,42^. This echoes the earlier notion that optimal planners should consider the *Value of Perfect Information* relative to the cost of obtaining it^43^. Planning depth has also been shown to vary with task demands^35^ and individuals’ subjective cost of cognitive effort^44^. Along the same lines, people often prune their decision trees when confronted with large potential losses^45^, a behavior that can be understood as a strategic adjustment of planning effort based on expected value and uncertainty reduction^33^. Under resource-rational principles, we would expect stochasticity to cause a reduction in planning effort. One model formalizes this by predicting that agents plan less when outcomes are less controllable^46^. While this model replicates patterns associated with decision making under learned helplessness and anhedonia^47^, it left its predictions about planning empirically untested. A related behavioral study found that participants in a multi-step farming task chose shorter-horizon crops after unexpected negative shocks, consistent with reduced planning under unreliable environments^48^. However, this finding focuses exclusively on negative outcomes, leaving open the question of whether reduced planning effort is a general response to stochasticity or specifically tied to the threat of future losses. More broadly, it remains unclear whether stochasticity alone, independent of outcome valence, drives reductions in planning effort.

In this work, we show that people reduce their planning effort in response to stochasticity. We designed a planning task where participants chose the best path down a visualized decision tree with the aim of maximizing the number of points earned. Keeping the same task structure, we manipulated a different form of stochasticity in each of our three experiments: reliability, volatility, and controllability. This structured approach allowed us to systematically examine how different forms of stochasticity shape planning effort. Finally, we developed several models to capture participants’ planning processes and used the best model to estimate planning effort as a function of stochasticity level.

## Results

### Unreliability decreases planning effort

We started by examining a simple form of stochasticity: reliability. Reliability refers to the consistency between an agent’s knowledge of the world and the actual state of the world. This type of stochasticity, as illustrated earlier by the example of planning with an outdated list of recommendations, arises when an agent’s information about rewards is imperfect.

**Fig. 1:**
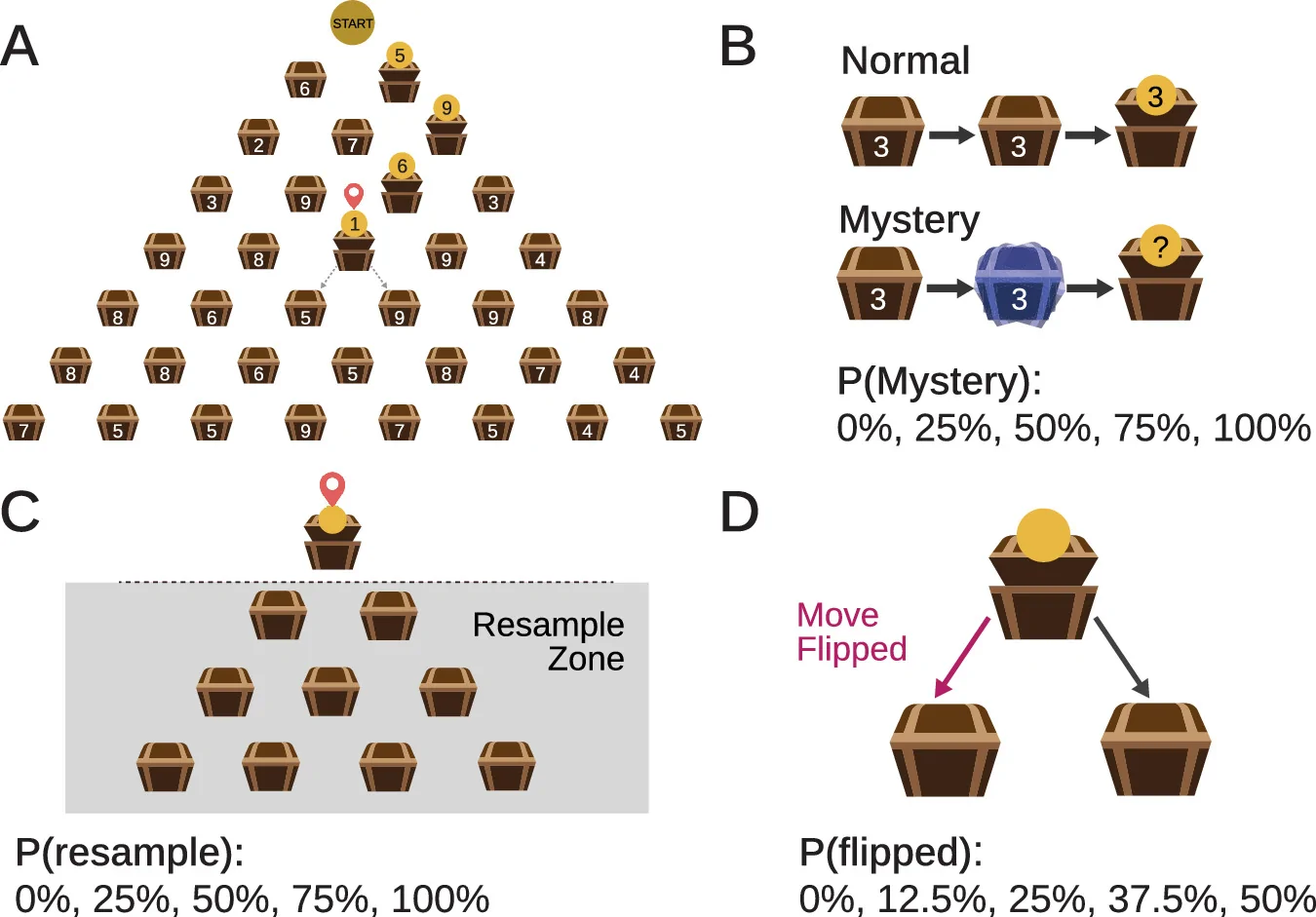
Task design. **A** Each game consists of a triangular arrangement of treasure chests. At each step, participants choose either left or right while progressing down the tree (arrows here are shown only for illustration). **B** Reliability: with probability *q*, the participant encounters a *mystery* treasure chest, which returns a random value between 1 and 9. **C** Volatility: with probability *q*, any treasure chest in the rows below the participant’s current position is re-drawn with a new random value between 1 and 9. **D** Controllability: with probability *q*, the participant’s choice (left or right) is flipped.

To study the effect of reliability on planning, we designed an online task loosely inspired by earlier work by Snider et al.^49^. The participant was instructed to accumulate as many points as possible while navigating a branching tree of treasure chests, making a binary left-or-right decision at each branch (Fig. 1A). Each chest displayed a number, drawn uniformly at random from 1 to 9. For clarity, we will refer to the number written on the front of the chest as the *label*, and the number of points received upon visiting the chest as *points earned*. To manipulate reliability, we introduced two types of treasure chests: *normal* chests, where the number of points each chest was worth was equal to its label, and *mystery* chests, where the label was uninformative, and the number of points earned was uniformly sampled at random from 1 to 9 (Fig. 1B). Participants were informed only of the overall probability (*q*_*r*_) that any given chest would be a mystery chest, which we varied across five levels: 0%, 25%, 50%, 75%, and 100%.

**Fig. 2:**
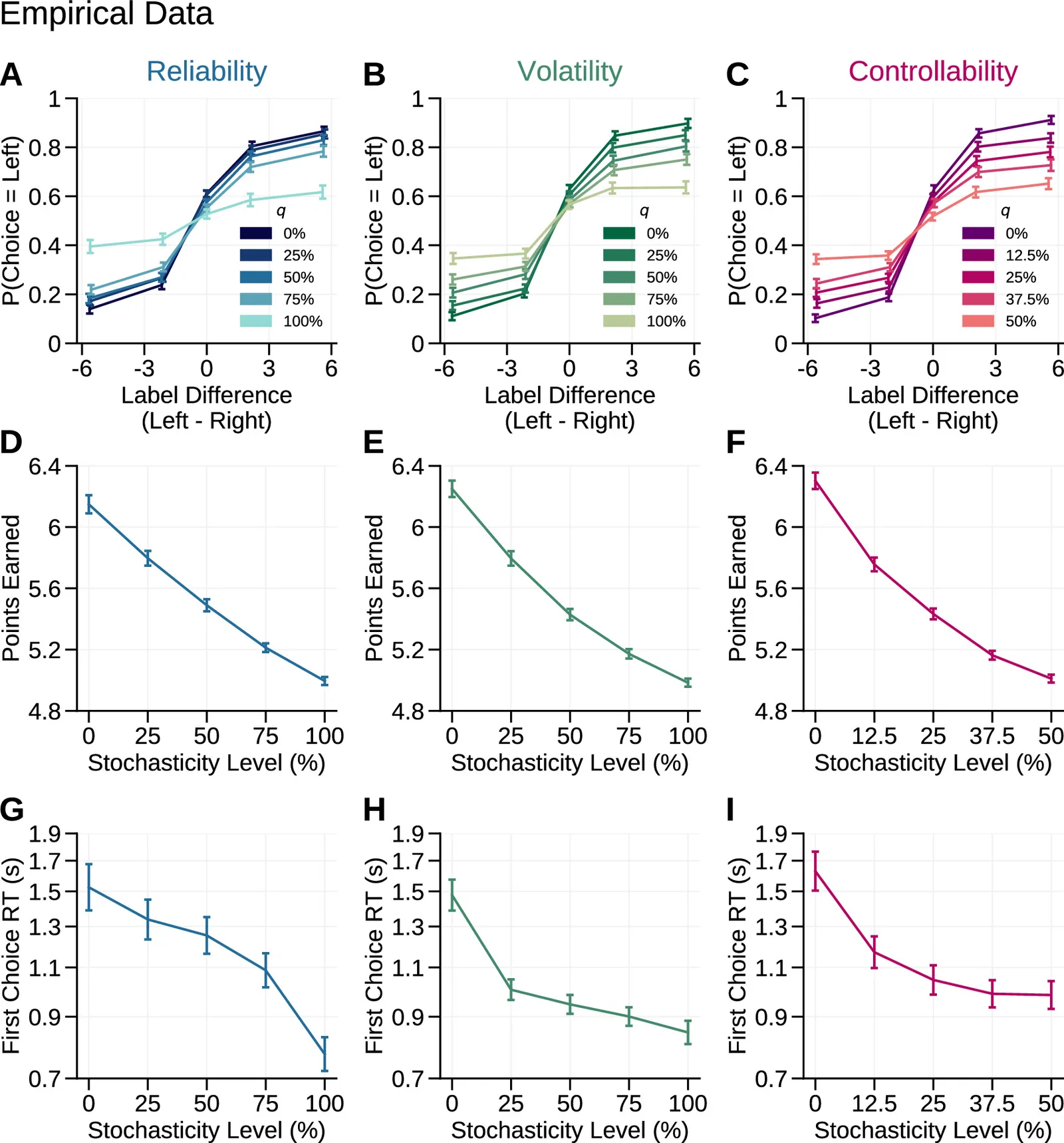
Participant behavior across three forms of stochasticity: reliability (*n* = 100 participants), volatility (*n* = 100), and controllability (*n* = 100). Error bars represent mean ± SEM across participants. Colors distinguish different forms of stochasticity (reliability in blue; volatility in green; controllability in magenta). **A–C** Probability of leftward choice based on label difference for different stochasticity levels *q*, shaded by stochasticity level. **D–F** Earnings per trial across different stochasticity levels. **G–I** first-choice response times as a function of stochasticity level, plotting on a log-scaled y-axis.

Participants learned the task effectively, as revealed by two patterns in the data. First, participants’ choices were systematically influenced by the chest labels, reflecting attention to cues about potential rewards. Specifically, participants showed a clear preference for selecting the higher-valued chest when the difference between the labels was larger (Fig. 2A; GLMM, main effect *β* = 0.39, 95% CI [0.33, 0.46], *χ*^2^(1) = 32300.0, *P* < 0.001). The magnitude of this effect was higher for lower stochasticity levels (GLMM, interaction *β* = − 0.38, 95% CI [−0.40, −0.37], *χ*^2^(1) = 3030.0, *P* < 0.001). Second, participants earned significantly more points in more reliable environments (Fig. 2D; LMM, *β* = −1.16, 95% CI [−1.27, −1.04], *t*_99_ = −19.8, *P* < 0.001), indicating that they strategically leveraged environmental reliability rather than making arbitrary choices. These results confirm that participants’ decision-making was driven by differences in reward value and that they shaped their strategies based on the level of reliability.

To assess planning effort behaviorally, we conducted exploratory analyses of response times as an empirical proxy for planning effort. This measure has been validated in other domains, such as chess and navigation, where longer thinking times are associated with more strategic processing and reflect a greater value of computation^50,51^. In our analysis, we focus primarily on the thinking time preceding the first choice in each game, when people spend the most time planning their upcoming choices.

Our analysis shows that first-choice response time decreases monotonically as environmental stochasticity increases (Fig. 2G; LMM, *β* = −0.63, 95% CI [−0.79, −0.47], *t*_98_ = − 7.76, *P* < 0.001). In other words, as the reliability decreases, participants invest less time in planning. This supports the interpretation that under reduced reliability, the expected benefit of extensive planning diminishes, leading participants to spend less effort.

### Volatility and uncontrollability decrease planning effort

So far, we have shown that in less reliable environments, participants spend less effort planning. However, reliability is only one example of the forms of stochasticity people encounter. Many real-world scenarios, such as planning a trip with shifting weather conditions or investing in volatile markets, feature a different kind of stochasticity: temporal fluctuation. In these scenarios, stochasticity diminishes as the future approaches and it is often possible to change course as the future becomes more certain. For example, when crafting an itinerary for a trip, one might choose to plan specific outdoor activities only once the weather forecast becomes less volatile. In our next task variant, we examined how this temporal fluctuation, or volatility, influences planning effort.

In the volatility condition, we preserved the overall structure of the task but introduced temporal fluctuations. In this condition, a chest’s label always corresponded to its point value. To introduce volatility, we introduced a probability *q*_*v*_ that any chest below the participant’s current row would be resampled at each step (Fig. 1C). This meant that chests further away were more likely to change before being reached, modeling the intuition that distant outcomes are less stable than immediate ones. We varied volatility across five levels, *q*_*v*_ = 0%, 25%, 50%, 75%, and 100%.

As before, we first confirmed that participants effectively learned the task. Indeed, participants’ decisions were influenced by the value difference in labels (Fig. 2B; GLMM, main effect *β* = 0.48, 95% CI [0.38, 0.58], *χ*^2^(1) = 35700.0, *P* < 0.001), and this effect was modulated by stochasticity level (GLMM, interaction *β* = −0.42, 95% CI [−0.44, −0.41], *χ*^2^(1) = 3610.0, *P* < 0.001). Furthermore, they leveraged low-volatility conditions to maximize their rewards, consistently earning more points in the low volatility conditions compared to the high volatility ones (Fig. 2E; LMM, *β* = − 1.26, 95% CI [−1.37, −1.15], *t*_99_ = −23.2, *P* < 0.001).

Consistent with the reliability condition, we found that as volatility increased, participants’ planning effort, as measured by first-choice response times, monotonically decreased (Fig. 2H; LMM, *β* = −0.49, 95% CI [−0.60, −0.38], *t*_99_ = −8.83, *P* < 0.001). This supports the idea that participants invested less cognitive effort when planning in highly volatile environments.

We have shown so far that stochasticity in rewards, through either unreliable or volatile environments, can drive decreases in planning effort. However, in many real-world scenarios, stochasticity can also affect the transition structure of the environment. This type of stochasticity is familiar to anyone who has learned a new skill, whether it’s playing a new instrument or driving a car. Sometimes, the result of an action is far from what was intended, adding an extra layer of unpredictability to the decision-making process. In the final variant of our task, we investigated how transition noise, or controllability, impacts planning effort.

In the controllability condition, the labels accurately reflected the point value of each chest and did not fluctuate with time. However, participants could not always rely on their choices being executed as intended. With probability *q*_*c*_, their chosen direction was reversed: pressing left would result in a rightward action, and vice versa. We varied *q*_*c*_ across five conditions: 0%, 12.5%, 25%, 37.5%, and 50%.

As in the other two conditions, participants’ choices were shaped by the interaction between stochasticity level and the label differences between the immediate following options (Fig. 2C; GLMM, main effect *β* = 0.41, 95% CI [0.34, 0.49], *χ*^2^(1) = 33400.0, *P* < 0.001), which was modulated by stochasticity level (GLMM, interaction *β* = −0.86, 95% CI [−0.89, −0.83], *χ*^2^(1) = 3700.0, *P* < 0.001). As before, participants earned significantly more points when the environment was more controllable (Fig. 2F; LMM, *β* = −2.54, 95% CI [−2.76, −2.32], *t*_98_ = −22.9, *P* < 0.001). Similarly, we found a negative relationship between uncontrollability and first-choice response times (Fig. 2I; LMM, *β* = −0.94, 95% CI [−1.14, −0.74], *t*_98_ = −9.44, *P* < 0.001).

Together, these findings show a consistent pattern: as stochasticity increases-whether through unreliability, volatility, or uncontrollability-people reduce their planning effort.

### Computational models of planning effort

**Fig. 3:**
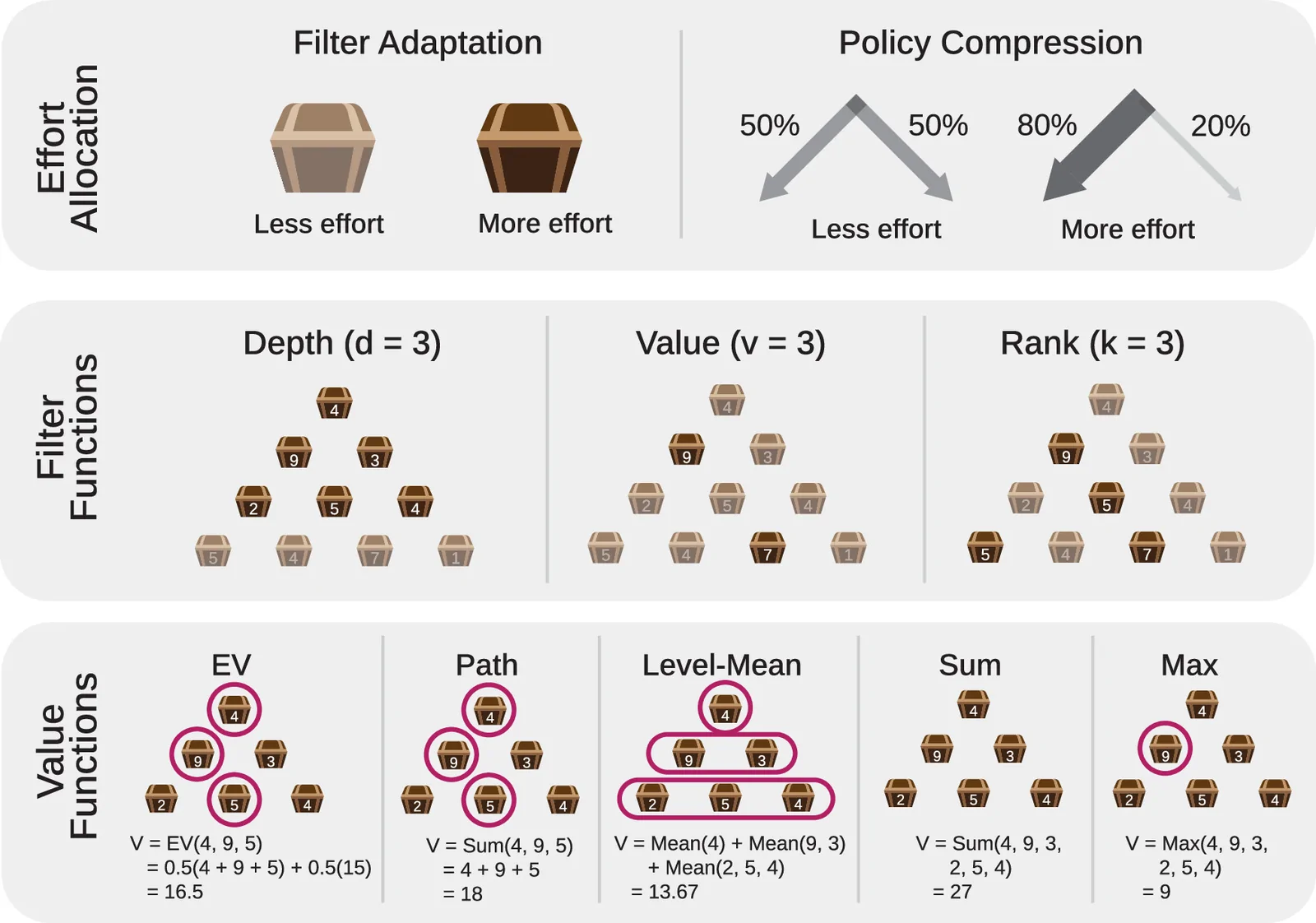
Model illustrations. Each model consists of a filter function and a value function to evaluate the left and right subtrees (only one subtree is shown here). The *depth filter* keeps only the top *d* rows. The *value filter* keeps only values ≥10 − *v*. The *rank filter* keeps only the top *k*-ranked values. For each filter function, several possible value functions are available. We show the value associated with each subtree under the reliability manipulation with *q*_*r*_ = 0.5. EV denotes expected value.

We used computational cognitive modeling to infer the algorithms that people use to make decisions across the three tasks (Fig. 3), allowing us to test how planning effort adapts to environmental uncertainty. Our models predicted participants’ choices at each decision point, assuming that each choice was guided by the subjective values assigned to the left and right subtrees of the decision tree. The greater the difference in these values, the more likely participants were to select the higher-valued option. We assumed conditional independence across choices, such that each decision depended only on the values of the immediately available subtrees.

Given the environment’s complexity, it is unlikely that participants evaluated all available information. Instead, we hypothesized that they employed *filtering* to focus on specific aspects of the tree. We hypothesized three main filtering approaches, grounded in task demands and cognitive plausibility: (1) **Depth filtering:** participants plan ahead only a limited number of steps, truncating their evaluation at a depth *d*; (2) **Value filtering:** participants attend only to treasure chests whose values are at least 10 − *v*; and (3) **Rank filtering:** participants attend only to the top *k* valued treasure chests, regardless of their absolute value.

After filtering, participants must assign scalar values to the left and right subtrees. We considered several *value functions* that vary in computational sophistication and cognitive effort: (1) **Expected Value (EV):** participants compute the expected value of all paths in the subtree, fully accounting for stochasticity, an optimal but computationally demanding approach. In the volatility condition, we excluded the EV model because simulating all possible states produced an exponential explosion of outcomes, making the computation both intractable and psychologically implausible. (2) **Path:** participants treat the environment as if it were deterministic, evaluating all possible paths and selecting the one with the highest reward; (3) **Level-Mean:** a coarser deterministic heuristic that averages rewards at each level of the tree and sums these level-wise means, approximating subtree value without tracking specific trajectories; (4) **Sum:** participants sum all treasure chest values within a subtree, treating it holistically; and (5) **Max:** participants consider only the maximum-valued chest within each subtree.

Only the EV model is fully optimal; the other models are *determinizing* because they treat the stochastic environment as if it were certain, replacing potentially intractable expected-value calculations with deterministic surrogates. Determinization has been used in the machine learning literature to simplify otherwise intractable problems^52–54^ but rarely examined in human cognition.

Finally, we modeled the participant’s choice to move left or right as a noisy action based on the difference in the values of the filtered subtrees, with the inverse temperature (or sensitivity) of the decision parameterized by *β*. We also included a generic lapse rate *λ* to capture purely random choices.

Having defined how participants filtered and evaluated information, we next modeled *effort allocation* as a function of stochasticity level in two distinct ways, each grounded in the decision-making literature: (1) **Filter adaptation (FA):** effort corresponds to considering more information. The exact form that this takes depends on the type of filtering in the model. For example, in the depth filtering case, increasing the depth threshold requires greater cognitive load. Thus, the filter threshold can vary across stochasticity levels while the policy sensitivity *β* remains constant. (2) **Policy compression (PC):** effort corresponds to deploying more precise control over the decision process. Here, filtering thresholds remain constant across stochasticity levels, but the sensitivity parameter *β* varies. This view aligns with theories of rational inattention, where *β* parameterizes the amount of resources (in information units, e.g., bits) available to represent a specific policy; a greater *β* corresponds to a more complex policy and therefore a greater cognitive cost^55–59^. We operationalize policy compression as adaptive changes in policy sensitivity (inverse temperature), abstracting away additional effects of explicit policy priors (e.g., perseveration or a default reference policy). These two formulations capture complementary notions of planning effort: one emphasizes how much of the environment is represented (filter adaptation), and the other emphasizes how sensitive the agent is to value differences (policy compression).

We fitted all models to participant behavior by minimizing the negative log likelihood (NLL). For model comparison, we used 5-fold cross-validated negative log likelihood. Our choice to use 5-fold cross-validated negative log likelihood reflects the fact that we are primarily interested in out-of-sample predictive accuracy and that some parameters have asymmetric impacts on particular model classes.

**Fig. 4:**
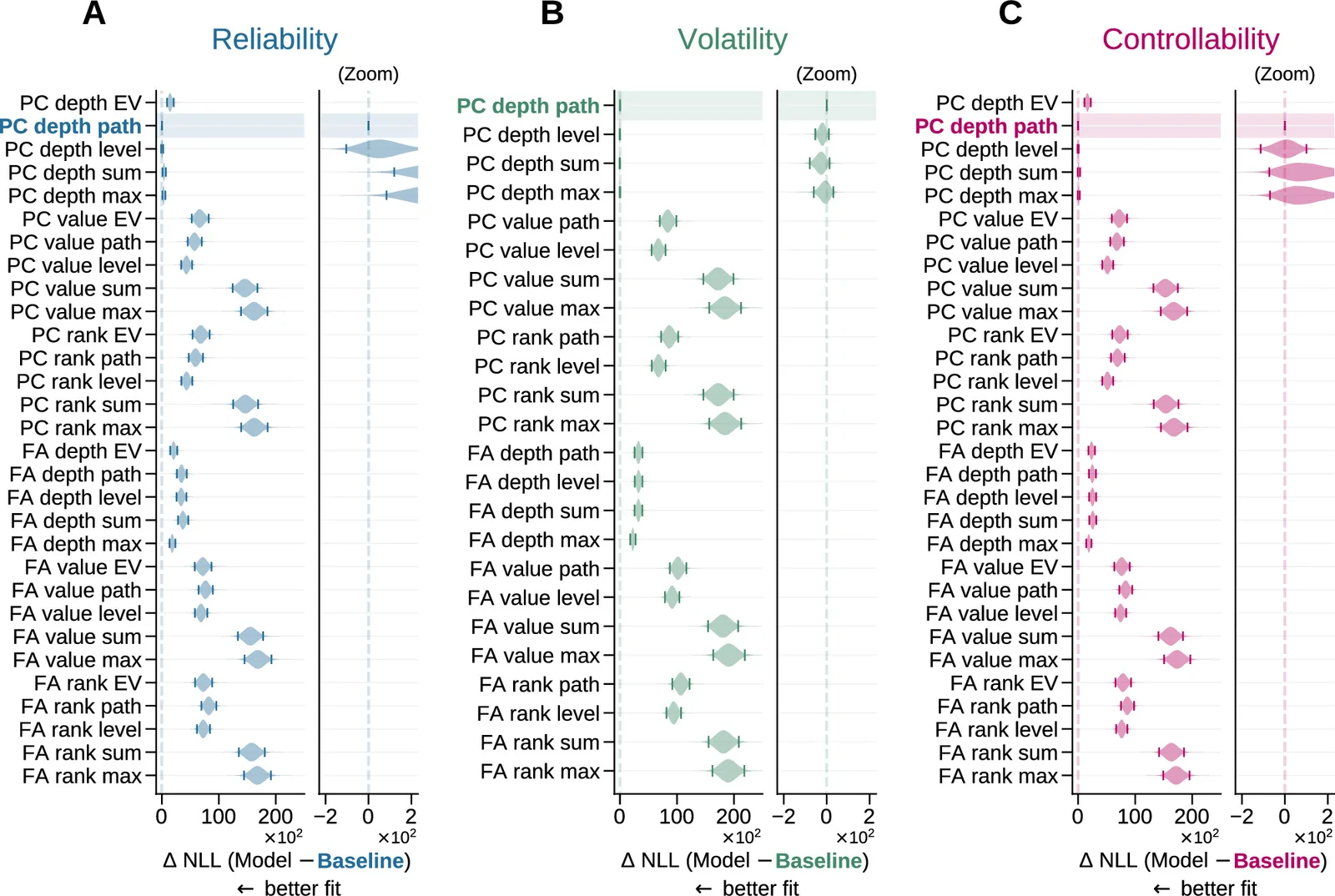
Model comparison (baseline: PC Depth-filtering Path). Bootstrapped differences (*n* = 100 participants per condition, 10^6^ resamples) between the 5-fold cross-validated negative log likelihoods of alternative models and the baseline model. Bars represent mean ± 95% CI of the bootstrap distribution. Each panel includes a zoomed-in inset to the right. **A** Reliability. **B** Volatility. **C** Controllability.

**Fig. 5:**
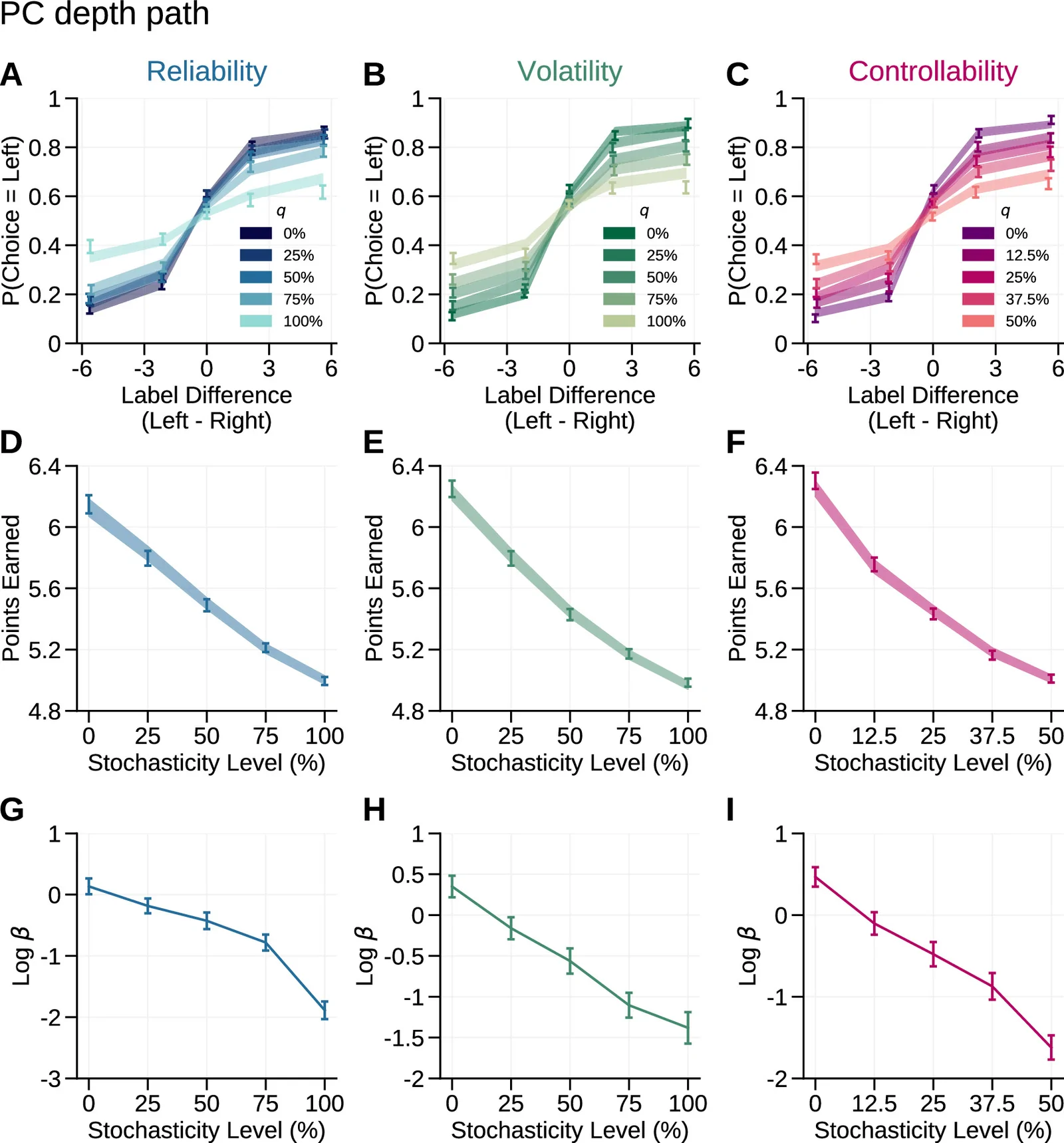
PC Depth-filtering Path model results (*n* = 100 participants per condition). **A–C** Probability of leftward choice based on label difference for different stochasticity levels *q*. Error bars (empirical) and shaded regions (model) represent mean ± SEM across participants. **D–F** Earnings per trial across different stochasticity levels. Error bars (empirical) and shaded regions (model) represent mean ± SEM across participants. **G–I** Stochasticity level versus model inverse temperature (log *β*). Error bars (model) represent mean ± SEM across participants.

In all conditions, the PC Depth-filtering Level-Mean and PC Depth-filtering Path models best fit participant behavior, with the PC Depth-filtering Sum and Max models also competitive in the volatility condition (Fig. 4). Importantly, the best-fitting models shared three key features: they all incorporated policy compression as a mechanism to modulate planning effort, relied on depth filtering to constrain the representational search space, and used determinizing value functions. The best-fitting models also provided quantitatively good fits to the behavioral trends reported earlier, specifically (1) the probability of choice as a function of label difference and stochasticity level, and (2) the points earned as a function of stochasticity level (Fig. 5A–F and Supplementary Fig. S2–S4). By contrast, the EV models predict that participants should be indifferent to value differences in fully stochastic environments, a prediction that is not supported by our data (Supplementary Fig. S5).

Next, we examined how planning effort varied across conditions in the winning models, as indexed by the model’s inverse-temperature parameter, *β*. We found that *β* decreased with environmental stochasticity across all three conditions for the PC Depth-filtering Path model (Fig. 5 G–I; reliability: LMM, *β* = −1.86, 95% CI [−2.19, −1.52], *t*_98_ = −11.1, *P* < 0.001; volatility: LMM, *β* = −1.76, 95% CI [−2.18, −1.34], *t*_98_ = −8.34, *P* < 0.001; controllability: LMM, *β* = −3.96, 95% CI [−4.62, −3.29], *t*_98_ = −11.8, *P* < 0.001), as well as the other competitive models (see Supplementary Note 5: Model comparison).

To assess participant-level heterogeneity in strategy selection, we conducted an exploratory random-effects Bayesian model comparison (RFX-BMC; Supplementary Note 8: Model heterogeneity). Results were not robust enough to support strong quantitative claims about individual differences in strategy use. We note that RFX-BMC captures heterogeneity across but not within individuals, and therefore does not speak to the possibility of hybrid or mixed strategies within a single participant.

Finally, we ran resource-rational simulations to test whether optimizing reward under effort costs could reproduce the observed adaptation in planning sensitivity (Supplementary Note 3: Resource-rational simulations). The simulated optimal $\log (\beta )$ decreased monotonically with stochasticity, demonstrating that our main effect can emerge from a simple reward-effort tradeoff.

Together, these results indicate that, regardless of the type of stochasticity, participants adopt simpler, more tractable planning strategies in stochastic environments. As stochasticity level increased, participants reduced cognitive effort via policy compression.

## Discussion

In this study, we investigated how stochasticity shapes both planning strategy and planning effort. We examined how people adjust their planning depth in response to three distinct forms of stochasticity: reliability, volatility, and controllability.

Across all three forms of stochasticity, participants’ behavior was best explained by models that incorporated policy compression, filtered by depth, and used determinizing value functions. Our results show that in all best-fitting models, the policy parameter *β* decreased monotonically as environmental stochasticity increased. This pattern suggests that participants strategically reduced their cognitive investment by adopting simpler policies when precise value comparisons were unlikely to pay off. This strategic simplification is a core feature of policy compression, a mechanism that allows agents to balance policy complexity and reward maximization^57,58^. This tradeoff is often framed as an information bottleneck, where a greater *β* corresponds to a larger information budget for implementing more complex and cognitively expensive policies^55–59^. Policy compression has been demonstrated in humans performing complex planning tasks^57^, and emerging evidence suggests that midbrain dopamine signals are responsive to policy complexity^60^. Additionally, the principle of policy compression has been used to design more robust and resource-efficient artificial agents^61^. Our work adds to this rich domain by identifying environmental stochasticity as a key factor that drives policy simplification, a signature of policy compression.

Our findings are qualitatively consistent with predictions of resource rationality, suggesting that people weigh the cognitive costs of planning against the potential benefits when deciding whether and how far to plan ahead. Existing work using simulated planning suggests that features like stochastic transitions and reward functions might diminish the utility of model-based planning relative to habit^62^. Additionally, policy compression has been directly linked to resource-rational concepts of attention allocation^55^. Consistent with this account, our resource-rational simulations yielded the same qualitative pattern as our behavioral data: optimal planning sensitivity (log *β*) decreased as stochasticity increased (Supplementary Note 3: Resource-rational simulations). An additional level of resource rationality may also operate in the selection of the model itself. In principle, an unconstrained optimal agent could compute expected future rewards directly. Under realistic computational constraints, however, resource-aware agents may instead favor simpler good-enough approximations that achieve favorable reward-effort tradeoffs. From this perspective, participants may first select a tractable planning strategy and then adapt the parameters of that strategy to different levels of stochasticity. Consistent with this interpretation, the best-fitting models in our study relied on simpler determinizing heuristics that treated the environment as effectively deterministic, even in settings where explicit expected-value computations remained computationally tractable in principle.

The technique of simplifying stochastic optimization by using a deterministic surrogate, known as determinization, is widely used in artificial agents and robotics as a good enough approximation of optimal actions^52–54^. A notable example is the FF-Replan algorithm^52^, which solves probabilistic planning tasks by assuming all nondeterministic outcomes follow the most likely result, a method called single-outcome determinization. Despite ignoring stochasticity, FF-Replan outperformed competitors in an international probabilistic planning competition. Since then, determinization principles have been applied to machine learning in a range of probabilistic planning tasks, with a focus on designing intelligent agents rather than explaining human behavior^53,54^. Our findings suggest that people also use determinization-like simplification when adapting to stochastic environments. This idea is broadly aligned with work suggesting that people use simplifying heuristics to adapt to the needs of their environment^35,63–68^. We do not intend to suggest that participants universally ignore expected value considerations when simplifying their planning. In our task, these determinizing heuristics were both simple and well-matched to the problem structure, likely because the branching factor at each decision point was small and the overall state space was tractable. In more complex environments, where each choice may open many possible continuations or the state space grows very large, we would expect participants to adopt alternative strategies, such as pruning^28,33,45^, to manage their computational load.

In a similar vein, our work underscores the limitations of traditional lottery tasks in behavioral economics, which typically focus on single-shot decisions where computing the expected value is straightforward and optimal^69–73^. However, as our findings suggest, this assumption may break down when individuals face an exponentially expanding decision space. The notion that people might adopt simpler, heuristic strategies in the face of complexity-whether due to branching possibilities or unpredictable outcomes–opens up a promising direction for future research.

Our research opens several avenues for future work. One open direction involves the study of individual differences in strategy selection and adaptation to stochasticity. The connection to resource rationality suggests that participants may themselves engage in multiple levels of strategy simplification, a phenomenon that may be better characterized at the individual level with richer datasets or adaptive designs. Our current experimental and computational methods do not capture within-individual mixing of strategies; although hybrid strategy use remains plausible, distinguishing such models would likely require task designs and computational frameworks explicitly constructed to dissociate dynamic strategy switching at an individual level.

A distinction is often made between stochasticity (also known as first-order uncertainty, risk, or aleatoric uncertainty) and second-order uncertainty (or epistemic uncertainty)^16,74,75^. Our study primarily focuses on aleatoric uncertainty, where the randomness in the environment is irreducible. By contrast, in epistemic uncertainty, learning more about the environment through exploration or simulation can reduce the overall uncertainty of the environment. For example, paradigms like the MouseLab MDP^42,76^ introduce costs to gathering new information about the environment. We would expect our findings to generalize to cases where it is possible in principle to obtain information but doing so is difficult or infeasible; in the example in our discussion of outdated recommendations, it may be possible to obtain more recent recommendations but doing so might be difficult in the moment or prohibitively costly. However, in other epistemically uncertain environments, increasing planning effort or policy sensitivity may become more valuable as the act of planning may reduce uncertainty over outcomes^33,77^. In these scenarios, we might expect people to expend more cognitive effort as the epistemic uncertainty increases, so long as the marginal value of uncertainty reduction outweighs the marginal cost^34^. This may explain the discrepancy in findings between studies that show that certain kinds of uncertainty motivate more myopic behavior, while others promote greater effort^41,78^. It is possible that first-order and second-order uncertainty have competing influences on people’s preferred depth of planning, leading to an optimal effort level that balances between planning in totally uncertain environments (where the world is too stochastic to be predictable) and totally certain ones (where the world is so stable that less effort needs to be exerted to plan ahead)^34^.

Another future direction is studying the developmental effects of unpredictable environments on planning effort. There is a growing body of evidence that stochastic environments can have long-lasting effects on cognitive development; studies have shown that growing up in unpredictable environments, particularly with family instability, can lead to reduced response inhibition and attentional control in children^79–81^. In these studies, stochasticity is often confounded with valence, making it difficult to tell whether these effects are a result of stochasticity in general or specifically the risk of adverse outcomes. Our work suggests that higher levels of stochasticity, independent of valence, during development may lead to enduring changes in how people approach future-oriented thinking and decision-making. Further research is needed to fully validate this hypothesis and understand the long-term impact of early exposure to stochastic environments.

## Methods

Our research complies with all relevant ethical regulations. The study protocol was approved by the Institutional Review Board of New York University, and all participants provided informed consent prior to participation.

### Experiment

Our experiment was preregistered on the Open Science Framework (OSF; https://osf.io/u6nfj), where we documented our hypotheses, experimental design, and planned analyses prior to data collection.

#### Participants

The Institutional Review Board of New York University approved the experimental protocol. Participants were recruited via Prolific, an online platform that connects eligible individuals with paid psychological studies. We recruited 100 participants per experiment across three experiments, resulting in a total of 300 unique participants. The sample size was specified in advance in our pre-registration and was determined on the basis of an experimental pilot, whose data were not included in the analyses reported here; no statistical method was used to predetermine sample size. The experiments were not explicitly randomized: each was run as a separate Prolific study, and participants were not assigned to experiments on the basis of any characteristic. Participants were restricted from enrolling more than once in the study. The investigators were not blinded to allocation during experiments and outcome assessment.

Eligible participants needed to be at least 18 years old, fluent in English, and based in the United States. To maintain data quality, participants were automatically excluded if they failed two out of three attention checks, refreshed or restarted the experiment, or exited before completing the study. Prolific automatically timed out sessions exceeding 140 minutes. The analyzed dataset comprises all complete sessions that passed the attention checks and timeout conditions from Prolific. Each participant faced only one form of stochasticity within an experiment. Within each experiment, the order in which stochasticity levels were presented was randomized within each session and mirrored across its two halves to control for order effects.

All participants provided informed consent prior to participation. We collected self-reported age and gender but did not separately collect information about biological sex. In the reliability experiment (*n* = 100), the mean age was 36.9 years (SD = 11.2), with 50 male, 48 female, and 2 nonbinary participants. In the volatility experiment (*n* = 100), the mean age was 37.8 years (SD = 10.6), with 50 male, 48 female, and 2 nonbinary participants. In the controllability experiment (*n* = 100), the mean age was 38.0 years (SD = 10.6), with 50 male, 48 female, and 2 nonbinary participants. No sex or gender-based analysis was performed because the study was not designed to test for sex, gender, or individual differences in planning behavior.

#### Compensation

Participants received a flat payment of $8 for completing the task, with the potential to earn additional performance-based bonuses. Before starting, they were informed that bonuses would be determined based on their performance in a randomly selected subset of 15 games. For each selected game, the participant’s earned reward was compared to the average expected reward of a randomly acting agent. The difference in points was multiplied by 10 to determine the bonus amount in dollars. If the resulting bonus was negative, no bonus was given.

#### Procedure

Participants first completed a brief demographic survey before proceeding to the main experiment.

Participants were provided with detailed step-by-step instructions, including diagrams illustrating the task setup. They also completed practice trials at each stochasticity level, which were excluded from the final analysis. Comprehension checks were added to verify their understanding of the task design and basic understanding of probability.

Each session consisted of 10 blocks, two for each stochasticity level. Each block contained 15 games. The stochasticity level remained constant within each block and was explicitly communicated to the participants. At the beginning of each block, participants confirmed the stochasticity level by pressing the corresponding number (1 for the lowest stochasticity and 5 for the highest). To control for order effects, the sequence of stochasticity levels was randomized and mirrored in the second half of the session. The true values of the treasure chests were randomly generated from a uniform distribution ranging from 1 to 9, inclusive. The stochasticity level was presented in multiple formats, as both a proportion and percentage (e.g., *In condition 4 there is a 3 in 4 (75%) chance that a given treasure chest is a mystery chest*).

#### Manipulations

For reliability, we introduced two types of treasure chests: normal chests, where the label accurately reflected the number of the points the participant would earn when visiting the chest, and mystery chests, where the label was irrelevant and the points were resampled uniformly at random from 1 to 9. The type of chest was revealed only after it was chosen, at which point mystery chests would turn blue, shake, and display their true value (Fig. 1B). Participants were not told in advance which treasure chests were normal or mystery chests. Instead, they were only given the probability that any given treasure chest would be a mystery chest (*q*_*r*_). We varied this probability across five conditions, *q*_*r*_ = 0%, 25%, 50%, 75%, and 100%. The 100% unreliability condition corresponds to an environment where every chest is a mystery chest, and the label is completely unreliable.

For volatility, we retained the same basic task structure but eliminated the concept of mystery chests. Unlike the reliability condition, however, the labels always accurately reflected reward values in the volatility condition. Instead, we introduced stochastic changes over time: at each turn, every chest below the current row had a probability *q*_*v*_ of being resampled, with its new label (and point value) drawn uniformly from 1 to 9 (Fig. 1C). Resampled chests were visually indicated by shaking and turning blue, providing real-time feedback about the changing labels. Although participants could not predict which specific chests would change, they were informed of the overall volatility level, *q*_*v*_, which varied across five conditions: 0%, 25%, 50%, 75%, and 100%. In the 100% volatile condition, every chest below the current row was resampled at each step.

To introduce controllability into the task, we modified the basic setup from the reliability condition. Here, the values of the treasure chests are fixed at the start, with no mystery chests, and the labels always reflect the true point value. Instead of changing rewards, stochasticity was introduced in the player’s movement after each choice. With probability *q*_*c*_, the player’s choice would be flipped-pressing left could result in moving right, and vice versa (Fig. 1D). A regular move was indicated by a temporary gray line pointing to the intended chest, followed by a move to that chest. A flipped move was indicated by an animation where a line pointing to the intended chest would turn red and rotate to the flipped chest. We varied *q*_*c*_ across five conditions: 0%, 12.5%, 25%, 37.5%, and 50%. The 50% condition corresponds to completely random actions, regardless of choice.

Each of these manipulations incorporated a temporary visual component that indicated to the participant whether the treasure chest was being resampled (in the case of reliability or volatility) or whether the player’s choice was flipped (in the case of controllability). Importantly, participants were not required to wait until the animation completed before making their next choice, allowing them to continue acting without waiting for the visual feedback to act if they chose to do so. This ensured that the manipulations introduced stochasticity without constraining choice timing.

### Mixed-effects models

All reported statistics in the results section were derived from either Generalized Linear Mixed-Effects Models (GLMMs) or Linear Mixed-Effects Models (LMMs). Our choice to use GLMMs and LMMs in place of ANOVA (as suggested in our preregistration) reflects our interest in treating stochasticity as a continuous variable varying along a spectrum, rather than solely as a set of discrete experimental conditions. These models allowed us to assess the significance of effects while accounting for individual differences through participant-specific random intercepts and random slopes, which capture variation in how strongly each participant is influenced by the predictors. Mixed effects models were fit using pymer4^82^, which provides a Python wrapper around the functions lmer and glmer from R library lme4^83^. Unless otherwise mentioned, all GLMMs were estimated using Laplace approximation and all LMMs were estimated using restricted maximum likelihood estimation (REML).

All statistical tests reported here are two-tailed. Fixed effects in the LMMs were assessed with two-sided *t*-tests using Satterthwaite-approximated degrees of freedom. Effects in the GLMMs were assessed with two-sided likelihood-ratio *χ*^2^ tests, computed as twice the difference in log-likelihood between the model including the effect and the nested model without it (${\chi }^{2}=2\,{{\Delta }}\,\log L$, d*f* = 1).

In line with best practices for mixed-effects modeling^84^, we adopted a principled approach to specifying random-effects structures. All models were first fit with the maximal random-effects structure justified by the design, including by-participant random intercepts and random slopes for all predictors of interest. If the maximal model failed to converge or yielded a singular fit, we simplified the random-effects structure stepwise. First, we removed correlations among random intercepts and slopes while retaining all random slopes. If this uncorrelated slopes model also failed, we further reduced the model to include only random intercepts. Finally, if this also failed to fit, we reduced the model to only include fixed effects. All reported results are based on the most complex model that converged reliably, adhering to best-practice recommendations for mixed-effects analyses.

The LMMs assume normally distributed residuals with constant variance. We did not test these assumptions formally, as such tests reject on departures far too small to affect inference at our number of observations. Response times (in seconds) were log-transformed (natural logarithm) before entry into the LMMs to reduce right skew (see *Response-time analysis* below). We found modest departures from normality that did not change the direction or the statistical significance of the stated results in the main analysis (see Supplementary Note 6.2: LMM Robustness). The GLMMs are binomial and assume no residual normality.

#### GLMM: Label difference vs. probability of leftward choice

We modeled trial-level binary choices using a binomial generalized linear mixed-effects model with a logit link function:

1 $$\,{{\mbox{logit}}}\,(P({y}_{ij}=1))={\beta }_{0}+{\beta }_{1}{x}_{ij}+{\beta }_{2}{q}_{ij}+{\beta }_{3}({x}_{ij}\cdot {q}_{ij})+{u}_{0j}+{u}_{1j}{x}_{ij}+{u}_{2j}{q}_{ij}$$

where:

- *y*_*ij*_ ∈ {0, 1} indicates whether participant *j* chose the left option on trial *i* (1 = left, 0 = right),
- *x*_*ij*_ is the label difference on that trial,
- *q*_*ij*_ represents the condition (e.g., stochasticity level),
- ${u}_{0j} \sim {{{\mathcal{N}}}}(0,{\sigma }_{{u}_{0}}^{2})$ is a participant-specific random intercept,
- ${u}_{1j} \sim {{{\mathcal{N}}}}(0,{\sigma }_{{u}_{1}}^{2})$ is a participant-specific random slope for *x*_*ij*_,
- ${u}_{2j} \sim {{{\mathcal{N}}}}(0,{\sigma }_{{u}_{2}}^{2})$ is a participant-specific random slope for *q*_*ij*_,
- *β*_3_ captures the interaction between label difference and condition.

To assess the effect of label difference on choice behavior, we conducted likelihood ratio tests by comparing nested models.

We first tested whether label difference influenced choice by comparing a main-effects model to a reduced model excluding *x*:

Main effects model:

2 $$\,{{\mbox{logit}}}\,(P({y}_{ij}=1))={\beta }_{0}+{\beta }_{1}{x}_{ij}+{\beta }_{2}{q}_{ij}+{u}_{0j}+{u}_{1j}{x}_{ij}+{u}_{2j}{q}_{ij}$$

Reduced model:

3 $$\,{{\mbox{logit}}}\,(P({y}_{ij}=1))={\beta }_{0}+{\beta }_{2}{q}_{ij}+{u}_{0j}+{u}_{2j}{q}_{ij}$$

A likelihood ratio test assessed whether including *x* improved model fit:

4 $${\chi }^{2}(df)=2\left(\log {{{{\mathcal{L}}}}}_{{{{\rm{main}}}}}-\log {{{{\mathcal{L}}}}}_{{{{\rm{reduced}}}}}\right)$$

We report the main effects coefficient *β*_1_ from the main-effects model.

Next, we tested whether the influence of label difference varied across conditions by comparing the main-effects model to a full model with an interaction term:

Full model:

5 $$\,{{\mbox{logit}}}\,(P({y}_{ij}=1))={\beta }_{0}+{\beta }_{1}{x}_{ij}+{\beta }_{2}{q}_{ij}+{\beta }_{3}({x}_{ij}\cdot {q}_{ij})+{u}_{0j}+{u}_{1j}{x}_{ij}+{u}_{2j}{q}_{ij}$$

Main effects model:

6 $$\,{{\mbox{logit}}}\,(P({y}_{ij}=1))={\beta }_{0}+{\beta }_{1}{x}_{ij}+{\beta }_{2}{q}_{ij}+{u}_{0j}+{u}_{1j}{x}_{ij}+{u}_{2j}{q}_{ij}$$

The interaction effect was tested using:

7 $${\chi }^{2}(df)=2\left(\log {{{{\mathcal{L}}}}}_{{{{\rm{full}}}}}-\log {{{{\mathcal{L}}}}}_{{{{\rm{main}}}}}\right)$$

We report the estimated interaction coefficient *β*_3_.

#### LMM: stochasticity vs. points earned

We modeled points earned per trial using a linear mixed-effects model with a fixed effect of stochasticity level and both a random intercept and a random slope for stochasticity for each participant:

8 $${y}_{ij}={\beta }_{0}+{\beta }_{1}{q}_{ij}+{u}_{0j}+{u}_{1j}{q}_{ij}+{\varepsilon }_{ij}$$

where:

- *y*_*ij*_ is the number of points earned by participant *j* on trial *i*,
- *q*_*ij*_ is the stochasticity level on trial *i*,
- ${u}_{0j} \sim {{{\mathcal{N}}}}(0,{\sigma }_{{u}_{0}}^{2})$ is a participant-specific random intercept,
- ${u}_{1j} \sim {{{\mathcal{N}}}}(0,{\sigma }_{{u}_{1}}^{2})$ is a participant-specific random slope for *q*_*ij*_,
- ${\varepsilon }_{ij} \sim {{{\mathcal{N}}}}(0,{\sigma }_{\varepsilon }^{2})$ is residual error.

The significance of the fixed effect was assessed using a *t*-test with Satterthwaite-approximated degrees of freedom, and we report the coefficient *β*_1_.

#### LMM: stochasticity vs log first-choice response time

We modeled response time preceding the first choice of each game using a linear mixed-effects model with stochasticity level as a fixed effect and both random intercepts and random slopes for stochasticity by participant. To account for skewness in the response time distribution, response times were log-transformed prior to analysis:

9 $$\log ({{\mbox{T}}}_{ij})={\beta }_{0}+{\beta }_{1}{q}_{ij}+{u}_{0j}+{u}_{1j}{q}_{ij}+{\varepsilon }_{ij}$$

where:

- T_*ij*_ is the first-choice response time for participant *j* in game *i*,
- *q*_*ij*_ is the stochasticity level in that game,
- ${u}_{0j} \sim {{{\mathcal{N}}}}(0,{\sigma }_{{u}_{0}}^{2})$ is a participant-specific random intercept,
- ${u}_{1j} \sim {{{\mathcal{N}}}}(0,{\sigma }_{{u}_{1}}^{2})$ is a participant-specific random slope for *q*_*ij*_,
- ${\varepsilon }_{ij} \sim {{{\mathcal{N}}}}(0,{\sigma }_{\varepsilon }^{2})$ is residual error.

The fixed effect of stochasticity was evaluated using a *t*-test with Satterthwaite-approximated degrees of freedom, and we report the coefficient *β*_1_.

#### LMM: stochasticity vs log inverse temperature

To assess whether log inverse temperature varied across levels of stochasticity, we fit a linear mixed-effects model to condition-level log inverse temperature estimates, including random intercepts and slopes for stochasticity:

10 $${y}_{jk}={\beta }_{0}+{\beta }_{1}{q}_{k}+{u}_{0j}+{u}_{1j}{q}_{k}+{\varepsilon }_{jk}$$

where:

- *y*_*jk*_ is the model-inferred log inverse temperature for participant *j* in condition *k*,
- *q*_*k*_ is the stochasticity level for condition *k*,
- ${u}_{0j} \sim {{{\mathcal{N}}}}(0,{\sigma }_{{u}_{0}}^{2})$ is a participant-specific random intercept,
- ${u}_{1j} \sim {{{\mathcal{N}}}}(0,{\sigma }_{{u}_{1}}^{2})$ is a participant-specific random slope for *q*_*k*_,
- ${\varepsilon }_{jk} \sim {{{\mathcal{N}}}}(0,{\sigma }_{\varepsilon }^{2})$ is residual error.

The effect of stochasticity on log inverse temperature was evaluated with a *t*-test using Satterthwaite-approximated degrees of freedom, and we report the coefficient *β*_1_.

### Computational models of planning effort

In our task, for every trial, the participant has two possible actions: choose left or choose right. We modeled planning as a function that takes the game state *s* and the stochasticity level *q* and returns the probability that the agent will choose left.

Since the agent cannot move back up the tree, the utility of choosing left must be some value function *V* over the left subtree *s*_*L*_, excluding the rightmost path (similarly, *s*_*R*_ for choosing right). The probability that the agent chooses left is the softmax over the difference between the value of a leftward choice and the value of a rightward choice. For a binary choice, the softmax function simplifies to a logistic function applied to the utility difference between left and right. Here, *β* is an inverse temperature parameter that controls decision stochasticity: higher values of *β* lead to more deterministic choices, whereas lower values produce more random decision-making. Additionally, we include a lapse parameter *λ* that is a scalar 0 ≤ *λ* ≤ 1, which corresponds to the probability that a participant chooses randomly between leftward and rightward actions.

11 $$P(a=L| s,q)=0.5\lambda+(1-\lambda )\,{{\mbox{Logistic}}}\,\left[\beta (V({s}_{L},q)-V({s}_{R},q))\right]$$

#### Filter functions

It is unreasonable to assume that participants consider the entire tree of possible paths from the root. Instead, participants may focus on a subset of nodes, performing a kind of *filtering* to select only the parts of the board they consider in their plans. This raises a key question: how do participants choose which nodes to attend to?

We consider three broad classes of filtering strategies-*depth-filtered*, *value-filtered*, and *rank-filtered* models-each capturing a distinct way participants might limit their planning horizon or attentional scope. In all cases, a filter function *F* selects a subset of the state *s* based on a parameter *f* (which may correspond to depth *d*, value threshold *v*, or rank cutoff *k*) and sets the remaining values to zero. Given the filtered state, the probability of choosing the left action is:

12 $$P(a=L| s,q)=0.5\uplambda+(1-\uplambda )\,\,{{\mbox{Logistic}}}\,\left[\beta \left(V(F({s}_{L},f),q)-V(F({s}_{R},f),q)\right)\right],$$

where *λ* is the lapse rate and *β* is the inverse temperature. The filter parameter *f* determines how much information is included in the computation of *V*(*s*, *q*).

- **Depth-filtering**. Participants may plan top-down, considering only a subset of the tree up to depth *d* from the root. This *planning depth* reflects how many future steps a participant evaluates. For instance, a participant with *d* = 3 considers only the next three possible moves. We define *d* ∈ {1, 2, 3, 4, 5, 6, 7}, where *d* = 1 corresponds to greedy choice behavior.
- **Value-filtering**. Alternatively, participants may focus on high-value regions of the board, filtering out all nodes below a value threshold *θ* = 10 − *v*. Values below *θ* are set to zero, leaving only the highest-valued regions visible for planning. Here, *v* ∈ {1, 2,…, 9} parameterizes the strictness of this threshold.
- **Rank-filtering**. Finally, participants may attend only to the top *k* values in the board, ignoring the rest. This rank-based filtering uses relative rather than absolute thresholding, with *k* ∈ {1, 2,…, 9}.

#### Value functions

After filtering, a value function *V*(*s*, *q*) maps the filtered state to a scalar estimate of expected reward. We consider several possible value functions, which differ in how they approximate the utility of the filtered subtree.

- **Expected value (EV)**. The optimal model computes the maximum expected reward along any path in the filtered subtree, taking into account transition stochasticity *q*. In the reliability and controllability conditions, this can be computed using dynamic programming, but becomes intractable in the volatility condition where the state space grows exponentially (up to 9^35^ possible states). Because such computation is infeasible for both participants and modeling, we omit the EV model from that condition.
- **Path**. In this model, participants behave as if outcomes were deterministic, ignoring stochasticity and evaluating the sum of values along the single best path in the filtered subtree. This differs from the expected value calculation because it does not consider stochasticity when estimating value. Note that this does not return a specific path but rather the sum of the values along the highest-valued path in the subtree.
- **Level-mean**. Here, participants approximate value by averaging rewards at each depth of the tree and summing these level-wise means. This provides a coarse, low-cost summary of tree structure without enumerating paths, serving as a simpler heuristic to the Path model.
- **Sum**. Participants may instead sum all node values within the filtered subtree, using the total as a surrogate for its value. This is a determinizing heuristic.
- **Max**. Finally, participants may focus only on the single most rewarding node in the subtree, taking *V*(*s*, *q*) to be the maximum value of any node. Like Sum, this provides a computationally simple but myopic estimate.

#### Effort allocation

Beyond the structure of filtering and valuation, we also model how participants allocate cognitive effort across conditions. We consider two mechanisms from the planning literature:

- **Filter adaptation (FA)**. Participants may modulate how much of the state they process by adjusting the filter parameter *f* (e.g., *d*, *v*, or *k*). FA models therefore include one global lapse rate *λ*, one global inverse temperature *β*, and one filter parameter *f*_*q*_ per stochasticity level (5 total), for 7 parameters in total.
- **Policy compression (PC)**. Alternatively, participants may hold the filtering constant but vary their decision sensitivity *β* across conditions, capturing effort allocation through the sharpness of their policy. PC models include one global lapse rate *λ*, one global filter parameter *f*, and one *β*_*q*_ per stochasticity level, again yielding 7 parameters in total.

### Model fitting

We fitted all models by maximizing the likelihood of observed participant choices. Each model contained:

- **Discrete filter parameters:***d* for depth-filtering, *v* for value-filtering, or *k* for rank-filtering models.
- **Continuous parameters:** the inverse temperature *β* (optimized in log-space, $-3\le \log \beta \le 3$) and lapse rate *λ* (0 ≤ *λ* ≤ 1).

Model fitting proceeded in a nested fashion, executing an exhaustive search over discrete parameters within the optimization of continuous model parameters:

1. **Inner optimization:** We exhaustively evaluated all possible discrete filter parameters. For FA models, we identified the combination of *f*_*q*_ values across the five stochasticity levels minimizing negative log-likelihood (NLL). For PC models, we identified the single global *f* minimizing NLL.
2. **Outer optimization:** Using scipy.optimize.minimize^85^ with L-BFGS-B^86,87^, we optimized $\log (\beta )$ and *λ* to minimize NLL given the best discrete parameters. We imposed hard bounds for numerical stability and interpretability (0 ≤ *λ* ≤ 1, $-3\le \log (\beta )\le 3$). FA models used a single *β* across all stochasticity levels; PC models allowed separate *β*_*q*_ values for each level.

To avoid local minima in our parameter space, we employed a multi-start optimization strategy. The policy compression models each have 6 continuous parameters (*β*_*q*_ for each stochasticity level *q* and a lapse parameter *λ*), while the filter adaptation models each have 2 continuous parameters (a global *β* and lapse *λ*). For each model, we randomly sampled 50 starting points in the continuous parameter space, such that $-3\le \log (\beta )\le 3$ and 10^−3^ ≤ *λ* ≤ 1. For each start, we tracked the best-performing parameter set based on the negative log-likelihood. This approach helped ensure robustness against local optima and provided a more comprehensive understanding of the parameter landscape.

We ran this optimization procedure for each of our models using 5-fold cross-validation to capture predictive accuracy. Our choice of 5-fold cross-validation over AIC/BIC as suggested in our pre-registration reflects the fact that (1) some parameters disproportionately affect the behavior of particular model subclasses more than others, and (2) we care about the out-of-sample predictive accuracy of our models when inspecting model fit. For our across-model analysis of goodness-of-fit and model comparison, we used 5-fold CV-NLL, consistent with best practices. For our within-model analysis (i.e., extracting winning parameter values for a given model), we re-ran the full fitting procedure for the model on the entire dataset to extract the parameter set that achieved the lowest negative-log-likelihood.

### Software

All analyses were performed in Python 3.12. Linear and generalized linear mixed-effects models were fitted with pymer4 0.8.2^82^, which calls the R 4.4.3 packages lme4 1.1-37^83^ and lmerTest 3.2-1 through rpy2 3.5.17. Numerical computation, optimization, and data handling used NumPy 2.2.6, SciPy 1.14.1^85^, pandas 2.2.3, and scikit-learn 1.8.0; figures were generated with Matplotlib 3.10.8. Exact versions of all packages are recorded in the environment.yml and requirements.txt files in the code repository.

### Reporting summary

Further information on research design is available in the Nature Portfolio Reporting Summary linked to this article.

## Supplementary information

Supplementary Information

Reporting Summary

Transparent Peer Review file

## Data Availability

The anonymized, raw trial-level behavioural data generated in this study—comprising participant choices, response times, points earned, and stochasticity condition for all 300 participants across the three experiments—have been deposited in the Open Science Framework (OSF) repository under https://doi.org/10.17605/OSF.IO/VYH8U^88^, and are publicly available without restriction. The deposit contains the raw trial-level data (data.tar.gz) together with the derived cross-validation splits (data_split.tar.gz) and model fits (fit.tar.gz) needed to reproduce the analyses. The deposit does not include participants’ individual-level demographic survey responses, which were collected only to describe the sample and are not used in any analysis, figure, or reported statistic; the aggregate demographics in the Methods are the only use made of them. They are withheld because participants did not consent to the release of individual-level demographic data. Every figure and table in this article and its Supplementary Information can be regenerated from these data using the analysis code (see Code availability).

## Code availability

All code, including experiment, analysis, and modeling code, is available on GitHub at https://github.com/WeiJiMaLab/stochasticity_planning_effort and archived on Zenodo under https://doi.org/10.5281/zenodo.22035888^89^.
