# Supplementary material for: Environmental stochasticity reduces human planning effort
Source: Nat Commun. 2026 Sep 28;17:11199. doi: 10.1038/s41467-026-78023-9 (PMC13714268; doi:10.1038/s41467-026-78023-9)
Supplement: Supplementary file 1 [file 41467_2026_78023_MOESM1_ESM.pdf]

Supplementary Information

Contents

Supplementary Note 1: Experiment design 2
1.1 General task instructions . . . 3
1.2 Manipulation-specific instructions and checks . . . 4
1.3 Attention Checks . . . 6

Supplementary Note 2: Algorithms 8
2.1 Filter functions . . . 9
2.2 Value functions . . . 11
2.3 Computing choice probabilities . . . 14

Supplementary Note 3: Resource-rational simulations 14
3.1 Estimating rewards under effort . . . 14
3.2 Cost of planning . . . 15
3.3 Simulation results . . . 17
3.4 Discussion . . . 17

Supplementary Note 4: Response times 18

Supplementary Note 5: Model comparison 19
5.1 PC Depth-filtering Level-Mean model . . . 20
5.2 PC Depth-filtering Sum model . . . 21
5.3 PC Depth-filtering Max model . . . 22
5.4 PC Depth-filtering EV model . . . 23

Supplementary Note 6: Mixed-effects models 24
6.1 G/LMM Convergence . . . 24
6.2 LMM Robustness . . . 25

Supplementary Note 7: Model and parameter recovery 27
7.1 Simulation procedure . . . 27
7.2 Model recovery . . . 27
7.3 Parameter recovery . . . 58

Supplementary Note 8: Model heterogeneity 63
8.1 Robustness of heterogeneity analysis . . . 63
8.2 Heterogeneity results . . . 63

Supplementary Note 1: Experiment design

Each participant was given a set of general instructions (Fig S1) and a set of manipulation-specific instructions based on the version of the task they were playing (Reliability, Volatility, or Controllability). Participants played the task in *blocks*, and were told at the start of each block what stochasticity level was associated with that block (Fig S1F). Each participant completed 10 blocks of 15 games each, for a total of 150 games. Blocks were presented in random order in the first half of the experiment and then the order was reversed in the second half to mitigate order effects on attention and gameplay. Prior to each game, participants were instructed to place their hands in a *ready* position to begin (Fig S1E). All experiment procedures, code, and instructions are openly available on GitHub at: https://github.com/WeiJiMaLab/human_planning_in_stochastic_environments.git.

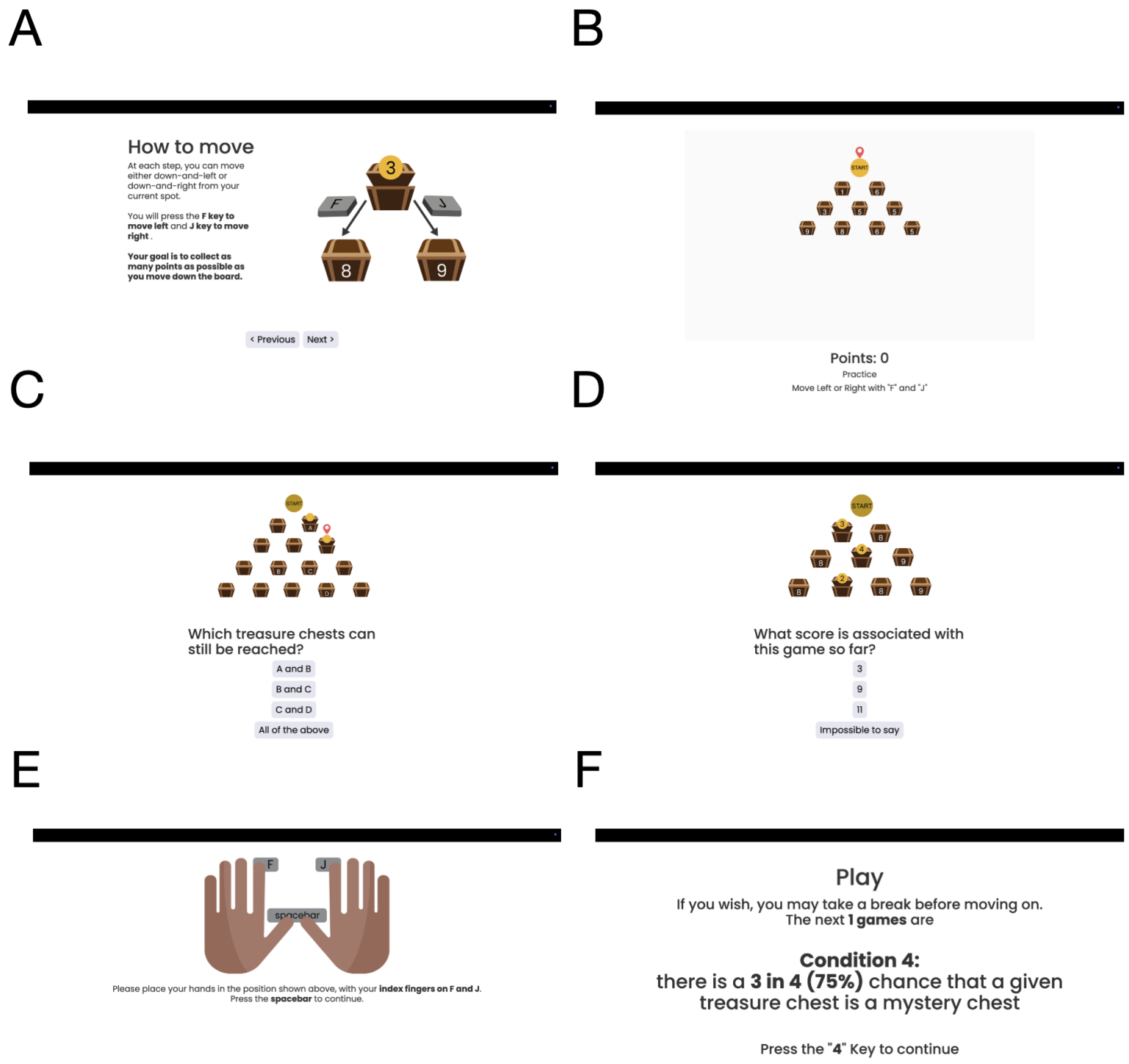

Fig. S1: Screenshots of participant instructions and stimuli. **A.** Example instruction slide. **B.** Example practice trial. **C–D.** Example comprehension checks. **E.** Hand placement instructions prior to the start of the game. **F.** Example block instructions; the number of blocks per game is 1 in the demonstration above but 15 in the real experiment.

Interactive demonstrations of the task are also available online for each manipulation: Reliability (https://triangle-treasure-demo.web.app/demoR), Volatility (https://triangle-treasure-demo.web.app/demoV), and Controllability (https://triangle-treasure-demo.web.app/demoC).

1.1 General task instructions

Participants were guided through the task via a series of interactive instruction screens (Fig S1A). They were first informed that the experiment would last approximately one hour, with a base pay of $8, and that bonus payments would be based on performance: *You will receive a bonus payment based on your performance on these games. The bonus will be calculated from a random sample of the games you play.* Participants were also warned that dropping out early or ending the experiment prematurely would result in no payment, and that attention checks were included; failing two checks would end the experiment with no compensation.

For the game mechanics, participants were told they were explorers seeking treasure, with the goal of earning as many points as possible. Each treasure chest was labeled with a number from 1 to 9, representing the points it contained, with higher numbers yielding more points. At each step, participants could move down-and-left or down-and-right from their current position, pressing the F key to move left and the J key to move right. Finally, participants completed a mini-version of the game as a practice round, which was not scored, allowing them to familiarize themselves with the movement mechanics and the overall task.

Participants completed one practice game (Fig S1B) using a simplified 4-row version of the task with no stochastic manipulations. This allowed them to experience movement mechanics, collect points, and see visual feedback. Practice trials were excluded from the main analysis.

Comprehension checks

After practice, participants completed four multiple-choice comprehension questions. If the participant failed to answer all comprehension checks correctly, they would be taken back to the beginning of the general instruction block.

1. **Goal of the game:** *What is the goal of the game?*
   - Collect the most points **(correct)**
   - Collect the least points
   - Finish the game as fast as possible
2. **Movement keys:** *Which of the following statements is correct?*
   - The J key is for moving left and the F key is for moving right
   - The arrow keys are used to navigate the board
   - The F key is for moving left and the J key is for moving right **(correct)**
3. **Reachable treasure chests:** (See Fig. S1C) *Which treasure chests can still be reached?*
   - A and B

- B and C
- C and D **(correct)**
- All of the above

4. **Score calculation:** (See Fig S1D) *What score is associated with this game so far?*

   - 3
   - 9 **(correct)**
   - 11
   - Impossible to say

1.2 Manipulation-specific instructions and checks

Participants were randomly assigned to one of three manipulations: **Reliability**, **Volatility**, or **Controllability**. Within their assigned manipulation, participants first received instructions describing the relevant task mechanics and stochasticity. They then completed a practice round at each stochasticity level on a full-sized board, allowing them to experience the manipulation-specific mechanics. After practice, participants completed multiple-choice comprehension questions to ensure understanding. If any question was answered incorrectly, participants repeated the entire manipulation-specific instruction block, including the practice rounds, until all comprehension questions were answered correctly.

Reliability

Participants were informed that treasure chests could be either **normal** or **mystery**. Normal chests contain the displayed number; mystery chests contain a **random number between 1 and 9**. Chest type is **completely random** and independent of location, value, or past choices. The five stochasticity conditions were presented along with their corresponding levels. Participants then completed the following comprehension checks:

1. In condition 3 there is a 50% chance that a given chest will be a mystery chest. If there are 10 treasure chests in total ...

   - Exactly 5 chests will be mystery chests
   - Less than 5 chests will be mystery chests
   - More than 5 chests will be mystery chests
   - It is impossible to know **(correct)**

2. In condition 1 there is a 0% chance that a given chest will be a mystery chest. If there are 10 treasure chests in total ...

   - 10 chests will be mystery chests
   - 0 chests will be mystery chests **(correct)**
   - 5 chests will be mystery chests
   - It is impossible to know

3. A treasure chest is more likely to be a mystery chest if ...

   - It has a large number written on the front

- Its neighbors are also mystery treasure chests
- The last treasure chest you visited wasn't a mystery treasure chest
- None of the above **(correct)**

4. You see an unopened treasure chest with a 5 written on the front. When you visit it, you discover it is a mystery chest. The number of points you get ...

   - must be equal to 5
   - must not be equal to 5
   - Can be any number between 1 and 9 **(correct)**
   - None of the above

Volatility

Participants were informed that some treasure chests lie in an **earthquake zone** where they might shake and receive a new value between 1 and 9. Shaking is **completely random** and independent of past shakes, chest value, or location. Participants were shown the five stochasticity conditions and their corresponding levels, followed by comprehension checks:

1. In condition 3 there is a 50% chance that a given chest in the earthquake zone will shake. If there are 10 treasure chests in the earthquake zone ...

   - Exactly 5 chests will shake
   - Less than 5 chests will shake
   - More than 5 chests will shake
   - It is impossible to know **(correct)**

2. In condition 1 there is a 0% chance that a given chest in the earthquake zone will shake. If there are 10 treasure chests in the earthquake zone ...

   - Exactly 10 chests will shake
   - Exactly 0 treasure chests will shake **(correct)**
   - Around 5 treasure chests will shake
   - It is impossible to know

3. On a given move, a treasure chest is more likely to shake if ...

   - It has a large number written on the front
   - Its neighbors also shake
   - It did not shake in the previous move
   - None of the above **(correct)**

4. One of the treasure chests in the earthquake zone is shaking. If it had a 3 written on the front before it started shaking, the value of the treasure chest after shaking is ...

   - Not equal to 3
   - Smaller than 3
   - A random value between 1 and 9 **(correct)**
   - None of the above

5. Which treasure chests are in the earthquake zone?
    - A only
    - B and C
    - B, C, and D **(correct)**
    - D only
    - All of the above

Controllability

Participants were told that occasionally their moves might be **flipped** (pressing left could move them right). Flipped moves are **completely random**, independent of past moves or chest values. Participants were presented with the five stochasticity conditions and their corresponding levels, followed by comprehension checks:

1. In a flipped move, if you press the F (left) key ...
    - You will move left
    - You will move right **(correct)**
    - You will move up
    - It is impossible to know
2. In condition 3 there is a 25% chance that a given move will be flipped. For every 4 moves ...
    - Exactly 1 move will be flipped
    - Less than 1 move will be flipped
    - More than 1 move will be flipped
    - It is impossible to know **(correct)**
3. In condition 1 there is a 0% chance that a given move will be flipped. For every 4 moves ...
    - No moves will be flipped **(correct)**
    - All moves will be flipped
    - Half of the moves will be flipped
    - It is impossible to know
4. A move is more likely to be flipped if ...
    - The previous move was flipped
    - The treasure chests in the future have high values
    - You move left
    - None of the above **(correct)**

1.3 Attention Checks

To ensure participants remained engaged and followed instructions, attention checks were embedded within the task. Each participant encountered 3 total attention checks throughout the experiment. Failure of two attention checks resulted in termination of the experiment.

During these trials, participants were explicitly told to press only the spacebar when prompted. Responses were recorded, and any deviations from the instructed key press were used to assess attentiveness. This procedure ensured data quality by identifying participants who were not adequately attending to the task.

Supplementary Note 2: Algorithms

We represent each state s from an egocentric perspective using an (i, j) coordinate system, where i represents the number of rows from the current position and j represents the number of rightward steps. For example, the player's immediate leftward choice would be to the treasure chest at $(1, 0)$, and the immediate rightward choice would be to the treasure chest at $(1, 1)$. For all unreachable board states (including undefined positions beyond the last row of the game), the value of $s_{i,j}$ is set to 0.

The left subtree of the current state corresponds to the lower-left portion of the matrix, specifically $s_{i+1:\text{end},j:\text{end}}$ with all entries above the diagonal set to zero. Similarly, the right subtree corresponds to the lower-right portion, $s_{i+1:\text{end},j+1:\text{end}}$. These subtrees effectively capture all future potential rewards contingent on the player's initial directional choice.

2.1 Filter functions

```
Input: s: state, array of shape (n, m)
Input: d: depth threshold
s′ ← s
for i = 0 to n − 1 do
    for j = 0 to m − 1 do
        if i > d then
            s′_{i,j} ← 0                ▷ zero out rows deeper than d
        end if
    end for
end for
return s′
```

Algorithm 1: Depth Filter

```
Input: s: state, array of shape (n, m)
Input: v: value threshold
s′ ← s
for i = 0 to n − 1 do
    for j = 0 to m − 1 do
        if s′_{i,j} < 10 − v then
            s′_{i,j} ← 0                ▷ zero out values below 10 − v
        end if
    end for
end for
return s′
```

Algorithm 2: Value Filter

Input: s: state, array of shape (n, m)
Input: k: rank threshold ($k \in \{1, \ldots, 9\}$)

1: $L \leftarrow \{s_{i,j} : 0 \leq j \leq i < n\}$ ▷ Lower-triangular board entries
2: $U \leftarrow$ sorted distinct values of L in descending order
3: **if** $k > |U|$ **then**
4:     **return** s ▷ No further masking when k exceeds the number of score levels on the board
5: **end if**
6: Let $t \leftarrow$ the k-th largest distinct value in U ▷ Top k treasure score levels
7: $s' \leftarrow$ `where`$(s \geq t,\ s,\ 0)$ ▷ Mask out elements below the k-th score level
8: **return** s'

Algorithm 3: Rank Filter

2.2 Value functions

function LEFT_SUBTREE(t)
  $l \leftarrow$ `zeros_like`(t) ▷ Initialize empty subtree
  $l_{i,j} \leftarrow t_{i+1,j}$ for all i, j where $i \geq j$ ▷ Copy left subtree elements where row $\geq$ column
  return l
end function
function RIGHT_SUBTREE(t)
  $r \leftarrow$ `zeros_like`(t) ▷ Initialize empty subtree
  $r_{i,j} \leftarrow t_{i+1,j+1}$ for all i, j where $i \geq j$ ▷ Copy right subtree elements where row $\geq$ column
  return r
end function

Algorithm 4: Helper Functions for Tree Operations

Input: s': filtered state, array of shape (n, m)
$t \leftarrow$ `zeros_like`(s') ▷ Table for dynamic programming
$t_{n-1,*} \leftarrow s'_{n-1,*}$ ▷ Initialize last row with values from s'
for $i = n - 2$ down to 0 **do**
  for $j = 0$ to i **do**
    $t_{i,j} \leftarrow s'_{i,j} + \max(t_{i+1,j}, t_{i+1,j+1})$ ▷ Best path value
  end for
end for
return $t_{1,0} - t_{1,1}$ ▷ Decision value: left vs right subtree

Algorithm 5: Path Value Algorithm

Input: s': filtered state, array of shape (n, m)
$l \leftarrow$ LEFT_SUBTREE(s')
$r \leftarrow$ RIGHT_SUBTREE(s')
$v_{left} \leftarrow \sum l$ ▷ Sum all values in left subtree
$v_{right} \leftarrow \sum r$ ▷ Sum all values in right subtree
return $v_{left} - v_{right}$ ▷ Decision value: left vs right subtree

Algorithm 6: Sum Value Algorithm

Input: s': filtered state, array of shape (n, m)

$l \leftarrow$ LEFT_SUBTREE(s')
$r \leftarrow$ RIGHT_SUBTREE(s')
$v_{left} \leftarrow \max(l)$ ▷ Maximum value in left subtree
$v_{right} \leftarrow \max(r)$ ▷ Maximum value in right subtree
return $v_{left} - v_{right}$ ▷ Decision value: left vs right subtree

Algorithm 7: Max Value Algorithm

Input: s': filtered state, array of shape (n, m)

$l \leftarrow$ LEFT_SUBTREE(s')
$r \leftarrow$ RIGHT_SUBTREE(s')
$v_{left} \leftarrow 0, \quad v_{right} \leftarrow 0$
for $i = 0$ to $n - 2$ **do**
    $v_{left} \leftarrow v_{left} + \frac{1}{i+1} \sum_{j=0}^{i} l_{i,j}$ ▷ Row-wise mean over valid subtree entries ($j \leq i$)
    $v_{right} \leftarrow v_{right} + \frac{1}{i+1} \sum_{j=0}^{i} r_{i,j}$
end for
return $v_{left} - v_{right}$ ▷ Decision value: left vs right subtree

Algorithm 8: Level-Mean Value Algorithm

Input: s': filtered state, array of shape (n, m)
Input: q: stochasticity level of state s
Input: E: expected value parameter

$t \leftarrow$ `zeros_like`(s') ▷ Table for dynamic programming
$t_{n-1,*} \leftarrow q \cdot s'_{n-1,*} + (1 - q) \cdot E$ ▷ Initialize last row
for $i = n - 2$ down to 0 **do**
    for $j = 0$ to i **do**
        $t_{i,j} \leftarrow q \cdot s'_{i,j} + (1 - q) \cdot E + \max(t_{i+1,j}, t_{i+1,j+1})$
    end for
end for
return $t_{1,0} - t_{1,1}$ ▷ Decision value: left vs right path

Algorithm 9: EV Value Algorithm (Reliability)

Input: s': filtered state, array of shape (n, m)
Input: q: stochasticity level of state s

$t \leftarrow$ `zeros_like`(s') ▷ Table for dynamic programming
$t_{n-1,*} \leftarrow s'_{n-1,*}$ ▷ Initialize last row
for $i = n - 2$ down to 0 **do**
    for $j = 0$ to i **do**
        $v_{left} \leftarrow q \cdot t_{i+1,j} + (1 - q) \cdot t_{i+1,j+1}$ ▷ Left choice expected value
        $v_{right} \leftarrow (1 - q) \cdot t_{i+1,j} + q \cdot t_{i+1,j+1}$ ▷ Right choice expected value
        $t_{i,j} \leftarrow s'_{i,j} + \max(v_{left}, v_{right})$
    end for
end for
$v_{left} \leftarrow q \cdot t_{1,0} + (1 - q) \cdot t_{1,1}$ ▷ Left choice at root
$v_{right} \leftarrow (1 - q) \cdot t_{1,0} + q \cdot t_{1,1}$ ▷ Right choice at root
return $v_{left} - v_{right}$ ▷ Decision value: left vs right choice

Algorithm 10: EV Value Algorithm (Controllability)

2.3 Computing choice probabilities

Input: s: state array s of shape n, m
Input: FILTER_FN: filtering function to apply
Input: VALUE_FN: value function to evaluate options
Input: $\log(\beta_q)$: (log) inverse temperature parameter
Input: λ: lapse rate

```
1: s' ← FILTER_FN(s)                      ▷ Apply filter to get filtered state
2: Δ ← VALUE_FN(s')   ▷ Compute value difference using appropriate value function
3: p_left ← 0.5λ+(1−λ)σ(Δ, β_q) ▷ Apply sigmoid and lapse (take exponent of log(β_q))
4: return p_left
```

Algorithm 11: Probability of $a = L$

Supplementary Note 3: Resource-rational simulations

While we did not explicitly fit a resource-rational model in our main analyses, it is helpful to consider how the framework relates to our work. Resource rationality predicts that people choose planning effort to maximize the trade-off between its benefits (higher expected reward) and costs (greater cognitive effort) [1, 2]. Here we use simulations to show how one of our winning mechanisms, policy compression with Depth-filtering Path planning, arises naturally from an optimization process which considers the tradeoff between getting higher expected rewards and the cost of increasing policy sensitivity. We demonstrate that this trade-off naturally yields our main result, that optimal planning effort (inverse temperature β) decreases as stochasticity increases.

3.1 Estimating rewards under effort

Let β denote the inverse temperature, which we interpret as a measure of planning effort, and let $q \in [0, 1]$ denote the stochasticity level.

For each stochasticity level q, with planning depth fixed at $d = 3$, we simulated the winning PC Depth-filtering Path algorithm on $N = 10{,}000$ randomly generated boards per experimental condition. Because β spans several orders of magnitude, we varied it in log-space: we sampled 20 values of $\log(\beta)$ linearly between -2 and 2 (i.e., $\log(\beta) \in [-2, 2]$), corresponding to $\beta \in [e^{-2}, e^{2}]$, and interpreted β as the variable reflecting the sensitivity or effort invested in planning. We define:

$$R(\beta, q; d{=}3) = \text{expected reward from Depth-filtering Path planning under stochasticity } q. \quad (1)$$

Two general patterns emerged (Fig. S2A–C). First, for a fixed β, as stochasticity q increases, the expected reward $R(\beta, q)$ decreases. Higher stochasticity diminishes the

benefit of effort. Second, for a fixed q, increasing β improves performance but saturates eventually, reflecting diminishing marginal returns of additional planning effort. The rate of saturation differs somewhat across conditions: in the volatility condition, rewards saturate earlier than in the reliability condition, reflecting compounding stochasticity across planning steps.

3.2 Cost of planning

We modeled the cognitive cost of planning as proportional to effort:

$$C(\beta) = \alpha\beta \tag{2}$$

where $\alpha > 0$ scales the cost of additional effort. We allow for the value of α to vary across individuals.

The *net utility* of planning with sensitivity β under stochasticity q is then:

$$U(\beta, q) = R(\beta, q) - C(\beta) = R(\beta, q) - \alpha\beta. \tag{3}$$

Without any cost term, the optimal solution would be to exert as much effort as possible (except when stochasticity is maximal, where all β yield equal expected reward). Introducing the cost term makes the optimal level of effort dependent on q (Fig. S2D–F).

To capture variability in cognitive cost across participants, we assumed:

$$\alpha = \max[0, a], \quad a \sim \mathcal{N}(\mu_\alpha, \sigma_\alpha), \quad \mu_\alpha = 5, \quad \sigma_\alpha = 3 \tag{4}$$

We chose these parameter values for illustrative purposes; the choice of the specific parameters does not affect the qualitative conclusions.

We simulated $N_{\text{part}} = 100$ participants by drawing one α per participant. For each simulated participant and stochasticity level q, we computed:

$$U(\beta, q) = R(\beta, q) - \alpha\beta, \quad \log(\beta) \in [-2, 2], \tag{5}$$

and converted them to choice probabilities using a softmax:

$$P(\beta \mid q, \alpha) = \frac{\exp\left(U(\beta, q)\right)}{\sum_{\beta'} \exp\left(U(\beta', q)\right)} \tag{6}$$

We then simulated $N_{\text{trials}} = 100$ trials for each $(\text{participant}, q)$ pair by sampling β^* from $P(\beta \mid q, \alpha)$. This produced a distribution of planning effort choices across participants and stochasticity levels, which we aggregated for analysis.

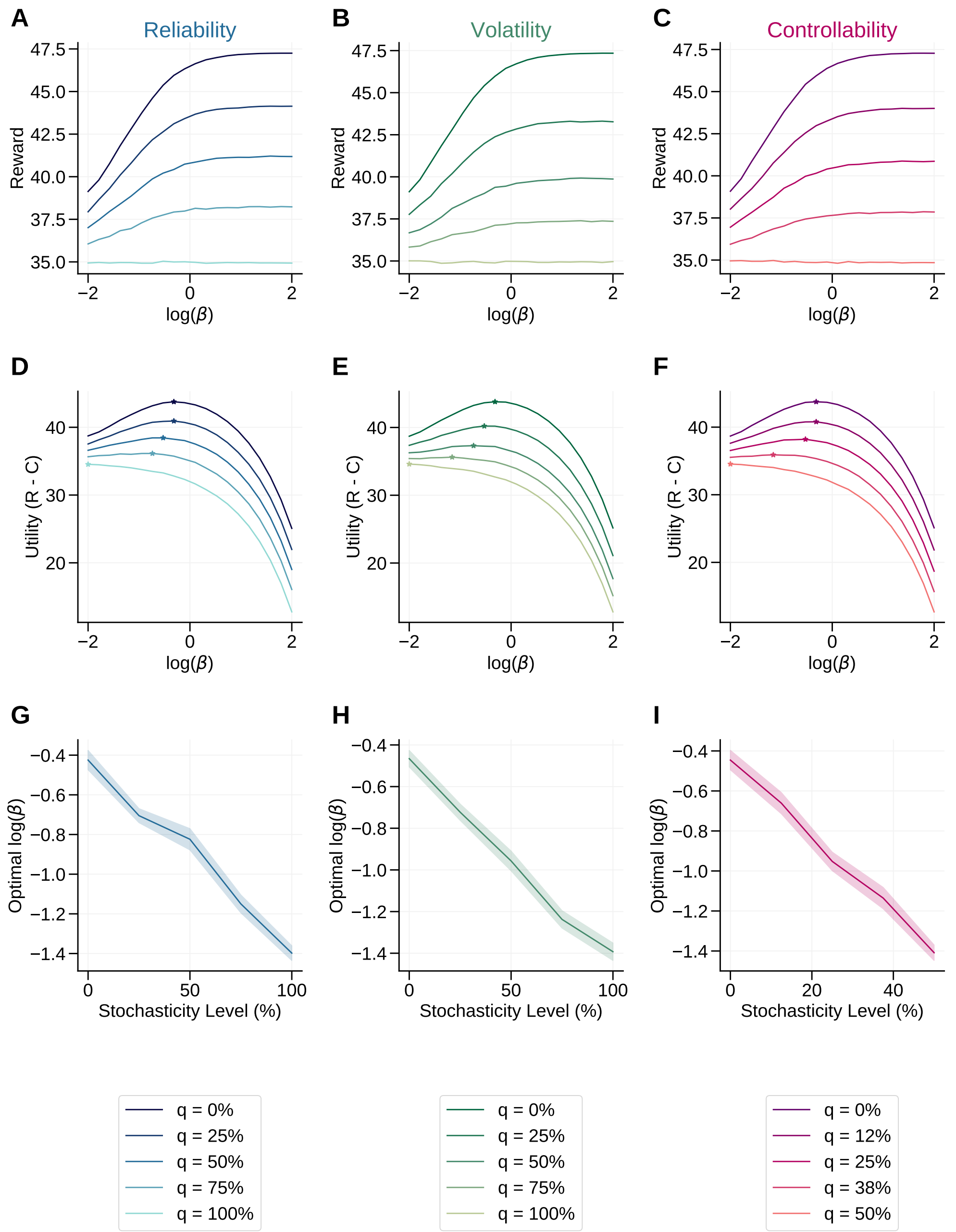

Fig. S2: Resource rationality simulated results. **(A - C)** Simulated expected rewards as a function of $\log(\beta)$, for each stochasticity level q, averaged over 10,000 board examples. **(D - F)** Example utility functions (here $\alpha = 3$) and their corresponding optimal $\log(\beta)$ value (denoted by stars). **(G - I)** Optimal $\log(\beta)$ as a function of stochasticity level, shaded region represents mean ± SEM, for 100 simulated participants with 100 samples each.

3.3 Simulation results

The simulated chosen effort $\beta^*(q)$ decreased monotonically with stochasticity q, consistent with the behavioral data (Fig. S2G–I). Across all three task variants, increasing stochasticity reduced the marginal benefit of investing in more precise value discrimination, leading resource-rational agents to select lower $\log(\beta)$ when uncertainty was greater. These simulations demonstrate that a simple reward–cost trade-off is sufficient to recover the key empirical pattern: when higher planning effort yields diminishing returns due to environmental stochasticity, agents optimally reduce planning sensitivity. This analysis illustrates that the policy compression mechanism observed in behavior is qualitatively consistent with a normative account in which planning effort is allocated adaptively.

3.4 Discussion

In these simulations, we approximated the expected reward $R(\beta, q)$ for different levels of planning effort β and stochasticity q by sampling from the task space. Participants are unlikely to explicitly compute such a function; instead, this exercise serves an illustrative purpose. It shows that an agent trading off reward and cognitive cost would rationally reduce planning sensitivity as stochasticity increases.

We modeled cost as linear in β,

$$C(\beta) = \alpha\beta, \tag{7}$$

reflecting that maintaining a sharper policy requires greater precision and cognitive effort. Other cost functions could be considered, but our aim here is simply to demonstrate that the qualitative pattern observed in data can arise naturally from a reward-effort tradeoff.

Taken together, these simulations indicate that the observed decrease in policy sensitivity under greater uncertainty is consistent with a resource-rational perspective: when the environment becomes less predictable, it is adaptive to expend less computational effort.

Supplementary Note 4: Response times

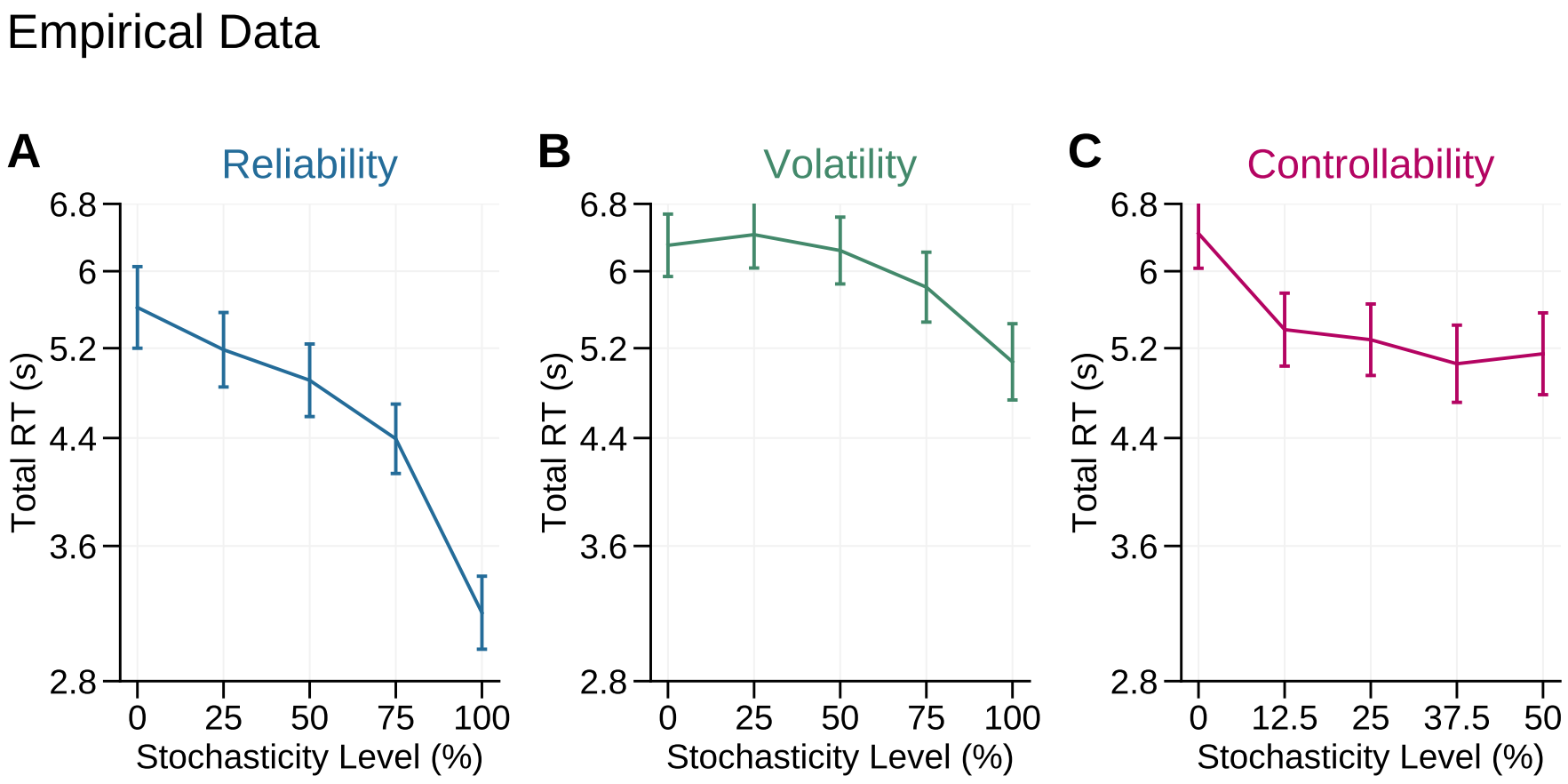

Fig. S3: Total response time results ($n = 100$ participants per condition). Error bars represent mean $\pm$ SEM across participants. **A–C.** Total response times as a function of stochasticity level, plotting on a log-scaled y-axis.

In the main text, we focused on first-move response times as a measure of cognitive effort, because participants spend the bulk of their planning before making their first move. However, because it is also possible for participants to deliberate mid-game, we also examined log total response times. Consistent with the first-move analysis, stochasticity level had a significant negative effect on total reaction time across all three conditions (Fig S3; Reliability: LMM, $\beta = -0.52$, 95% CI $[-0.65, -0.39]$, $t_{98} = -7.97$, $P < 0.001$; Volatility: LMM, $\beta = -0.21$, 95% CI $[-0.32, -0.11]$, $t_{99} = -3.95$, $P < 0.001$; Controllability: LMM, $\beta = -0.41$, 95% CI $[-0.60, -0.21]$, $t_{98} = -4.17$, $P < 0.001$). These results reinforce our main conclusion that higher stochasticity reduces planning-related response times.

Supplementary Note 5: Model comparison

In the main text, we reported results for the Policy Compression (PC) Depth-filtering Path model, which best captured participant behavior across all three conditions. We also found that the PC Depth-filtering Level-Mean model performed comparably to the PC Depth-filtering Path model in all stochasticity conditions, and that the PC Depth-filtering Sum and PC Depth-filtering Max models were competitive specifically in the volatility condition. We demonstrate that for each competitive alternative model, the same qualitative conclusions hold as in the main text. Finally, we illustrate why the PC Depth-filtering EV model fails to capture participant behavior in the cases where the expected-value computation is tractable (reliability and controllability).

5.1 PC Depth-filtering Level-Mean model

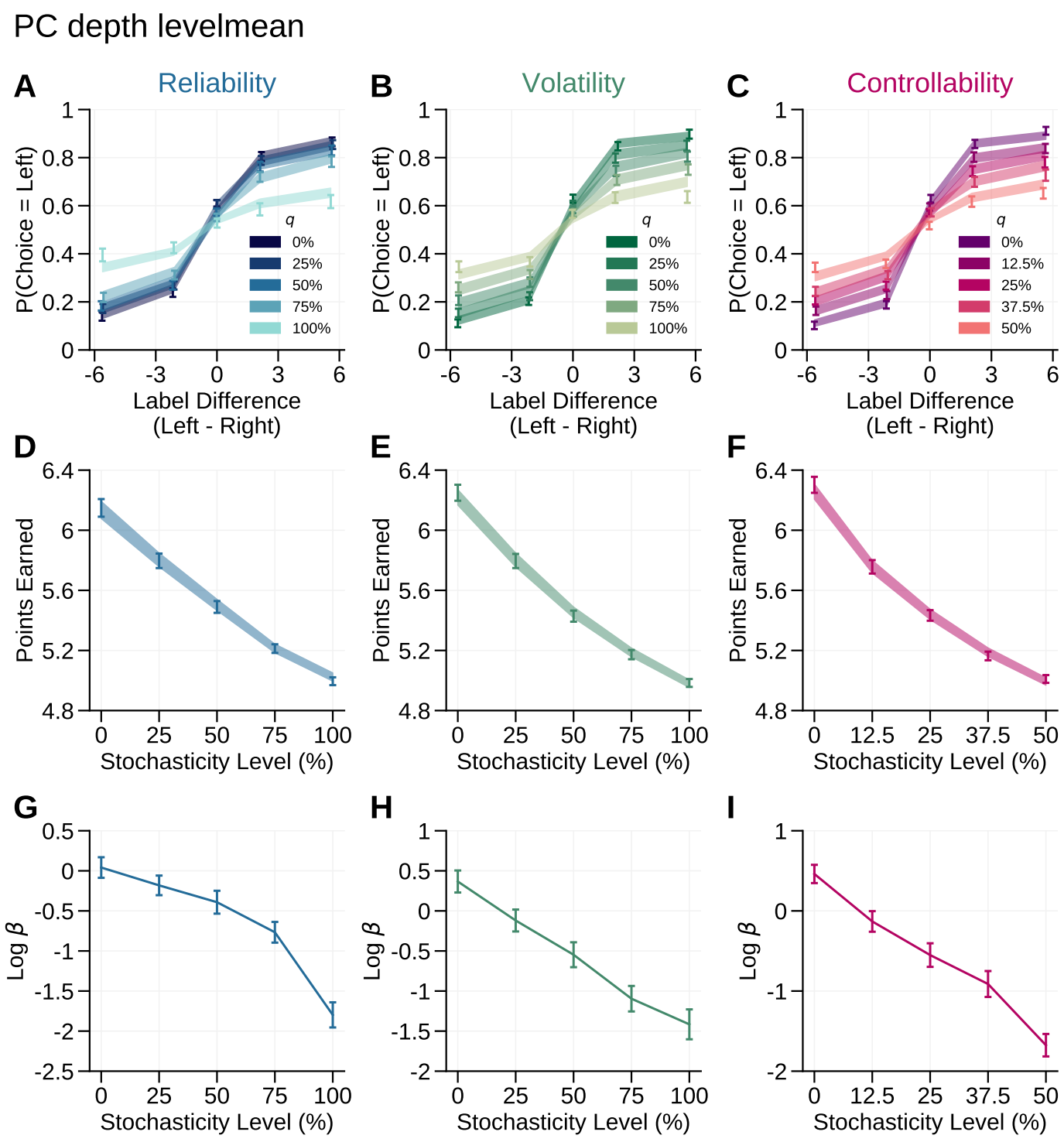

Fig. S4: PC Depth-filtering Level-Mean model results ($n = 100$ participants per condition). **A–C.** Probability of choosing left based on the difference between the immediate next values, segmented by stochasticity level q. Error bars (empirical) and shaded regions (model) represent mean ± SEM across participants. **D–F.** Earnings per trial across different stochasticity levels. Error bars (empirical) and shaded regions (model) represent mean ± SEM across participants. **G–I.** Stochasticity level versus model inverse temperature (log β). Error bars (model) represent mean ± SEM across participants.

The PC Depth-filtering Level-Mean model also outperformed other models in the model comparison. Similar to the PC Depth-filtering Path model that we highlight in the main text, this model effectively captures the trends in participant data (Fig S4A–F). Also similar to the PC Depth-filtering Path model, we found that increasing stochasticity level decreased $\log(\beta)$ across all three stochasticity types (Fig. S4G–I; Reliability: LMM, $\beta = -1.71$, 95% CI $[-2.08, -1.33]$, $t_{98} = -9.1$, $P < 0.001$; Volatility: LMM, $\beta = -1.82$, 95% CI $[-2.23, -1.40]$, $t_{98} = -8.7$, $P < 0.001$; Controllability: LMM, $\beta = -4.04$, 95% CI $[-4.63, -3.46]$, $t_{99} = -13.7$, $P < 0.001$).

5.2 PC Depth-filtering Sum model

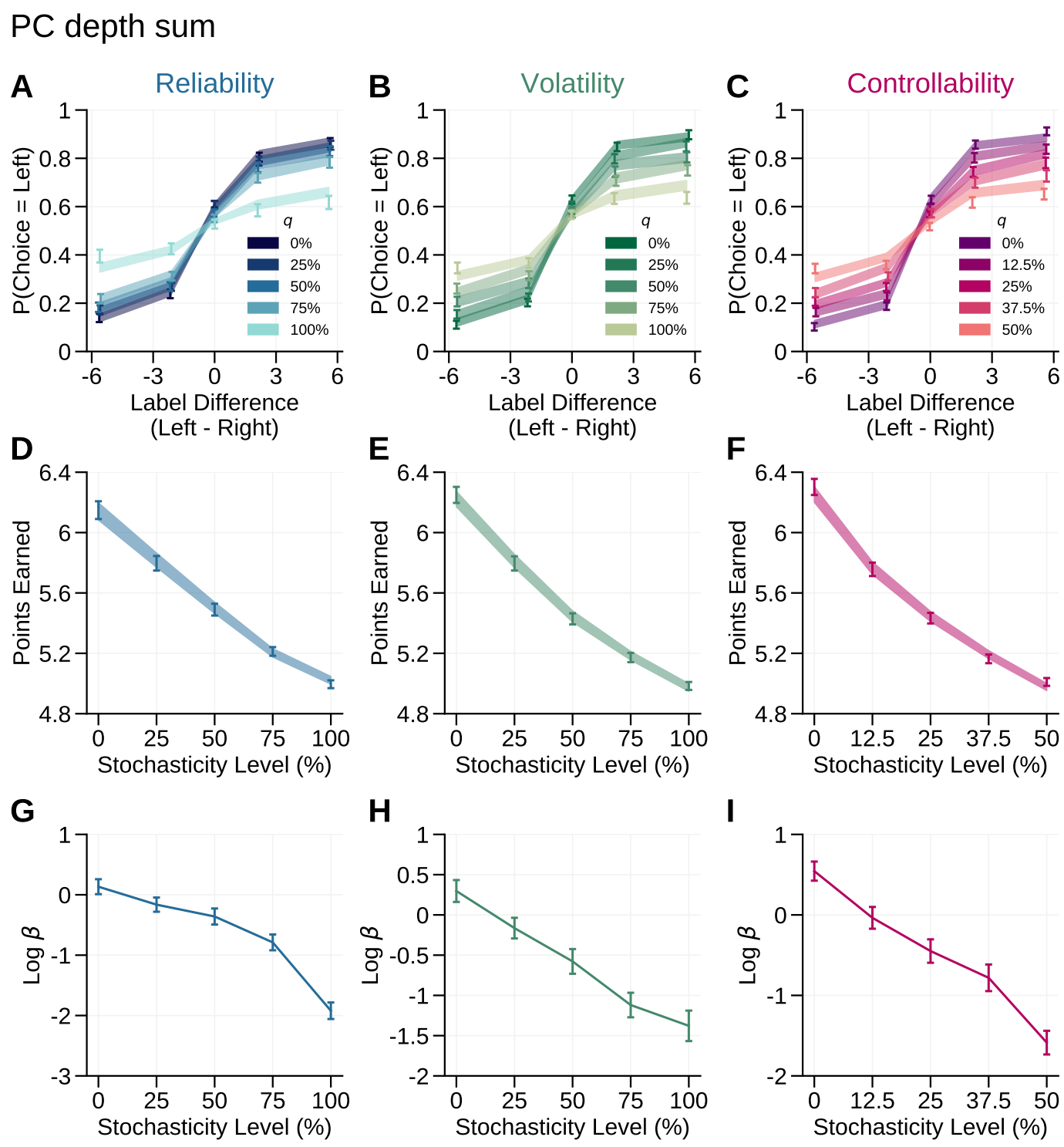

Fig. S5: PC Depth-filtering Sum model results ($n = 100$ participants per condition). **A**–**C.** Probability of choosing left based on the difference between the immediate next values, segmented by stochasticity level q. Error bars (empirical) and shaded regions (model) represent mean ± SEM across participants. **D**–**F.** Earnings per trial across different stochasticity levels. Error bars (empirical) and shaded regions (model) represent mean ± SEM across participants. **G**–**I.** Stochasticity level versus model inverse temperature (log β). Error bars (model) represent mean ± SEM across participants.

The PC Depth-filtering Sum model was competitive in the volatility condition. We found that in the volatility condition, increasing stochasticity level decreased $\log(\beta)$ (Fig S5H; LMM, $\beta = -1.72$, 95% CI $[-2.13, -1.31]$, $t_{98} = -8.35$, $P < 0.001$).

5.3 PC Depth-filtering Max model

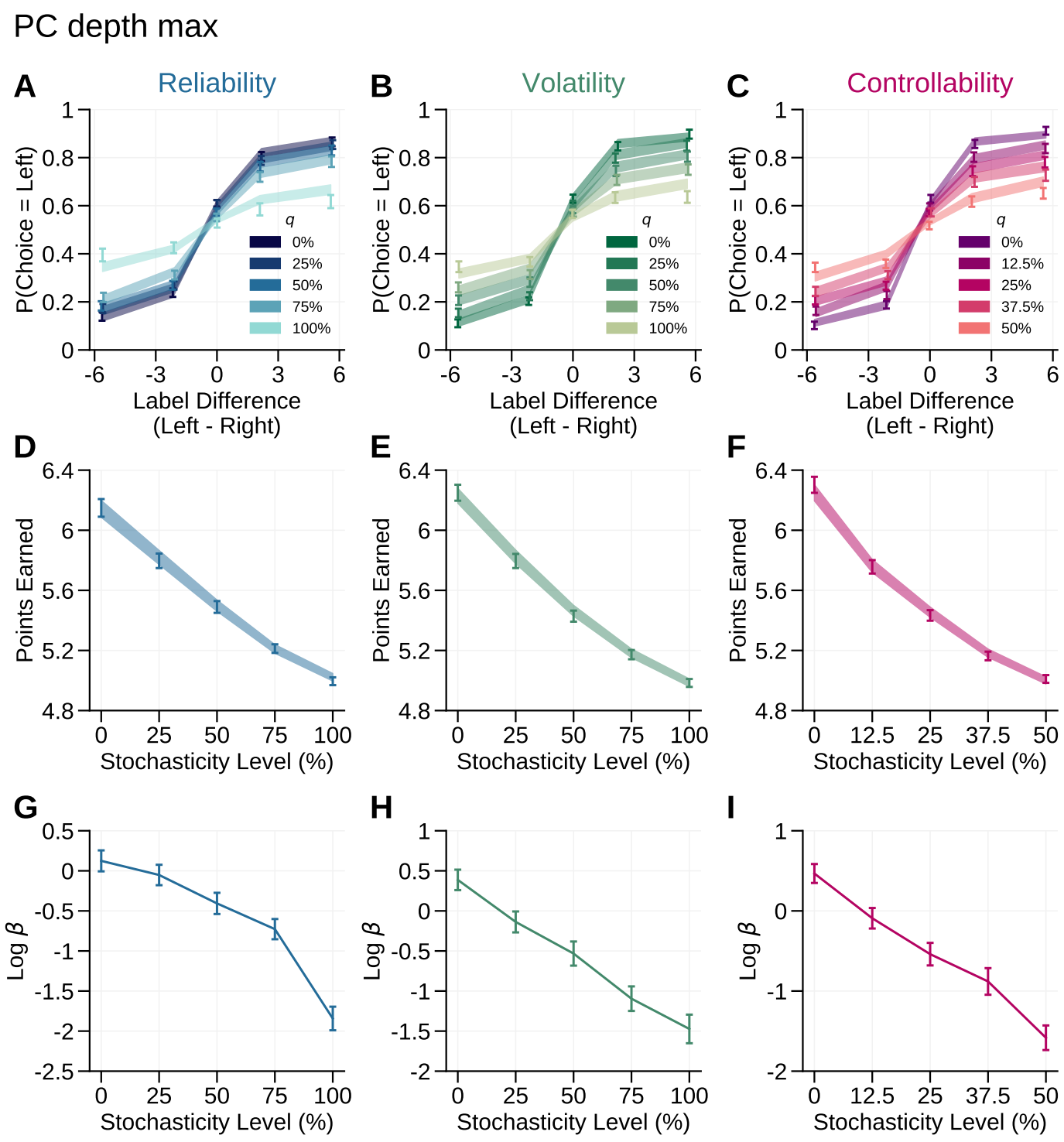

Fig. S6: PC Depth-filtering Max model results ($n = 100$ participants per condition). **A–C.** Probability of choosing left based on the difference between the immediate next values, segmented by stochasticity level q. Error bars (empirical) and shaded regions (model) represent mean ± SEM across participants. **D–F.** Earnings per trial across different stochasticity levels. Error bars (empirical) and shaded regions (model) represent mean ± SEM across participants. **G–I.** Stochasticity level versus model inverse temperature (log β). Error bars (model) represent mean ± SEM across participants.

The PC Depth-filtering Max model was competitive in the volatility condition. We found that in the volatility condition, increasing stochasticity level decreased $\log(\beta)$ (Fig S6H; LMM, $\beta = -1.87$, 95% CI $[-2.25, -1.49]$, $t_{98} = -9.77$, $P < 0.001$).

5.4 PC Depth-filtering EV model

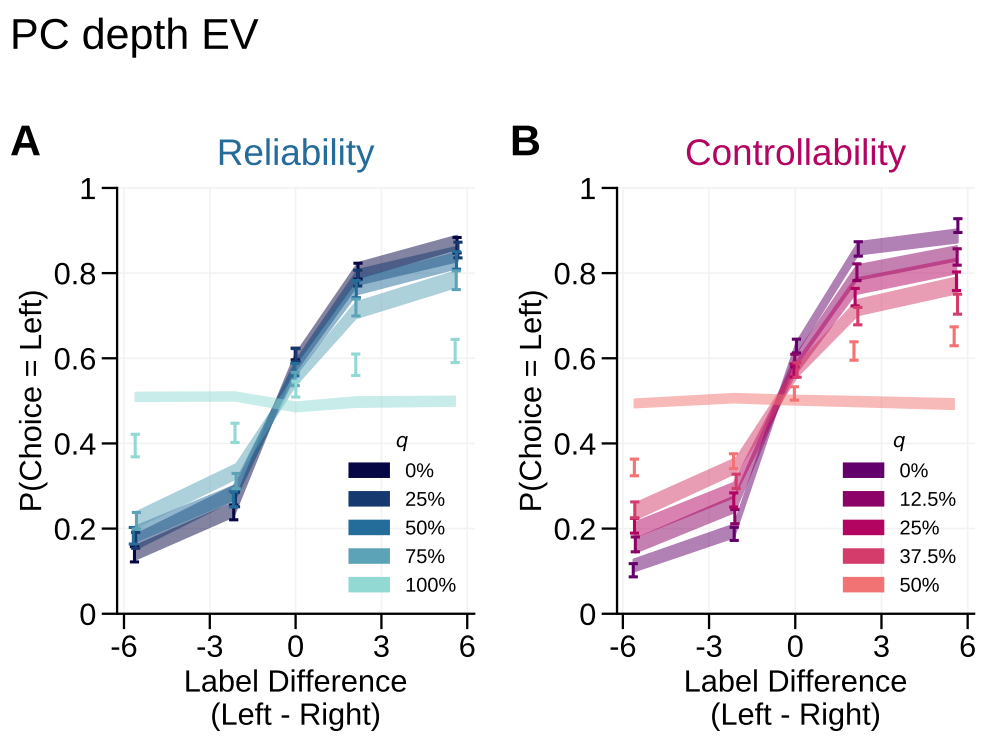

Fig. S7: PC Depth-filtering EV model checking ($n = 100$ participants per condition). Error bars (empirical) and shaded regions (model) represent mean ± SEM across participants. Probability of choosing left based on the difference between the immediate next values, for different stochasticity levels q. **A.** Reliability. **B.** Controllability.

Here we illustrate why the PC Depth-filtering EV model falls short relative to the determinizing alternatives. Recall that EV value function is only tractable in the reliability and controllability conditions; in these conditions it is informative to look at how the model describes the relationship between (greedy) label difference and probability of moving left. Looking at the model checking plots (Fig S7), we can see that in the highest stochasticity level (100% in reliability and 50% in controllability), the model predicts that participants would be indifferent to the label differences, having roughly chance level of choosing left for all label differences. Instead, what we find is that participants consistently continue to factor label difference into their decisions despite the fact that it is suboptimal, suggesting that they are determinizing rather than implicitly calculating the expected value of each path.

Supplementary Note 6: Mixed-effects models

6.1 G/LMM Convergence

Model Name	Stochasticity Type	Variable	Formula / Status
Empirical	Controllability	y = P(left) x = label diff	`y ~ x * conditions + (1 + x + conditions|participants)`:Converged
		y = log(firstRT)	`y ~ conditions + (1 + conditions|participants)`:Converged
		y = log(totalRT)	`y ~ conditions + (1 + conditions|participants)`:Converged
		y = points	`y ~ conditions + (1 + conditions|participants)`:Converged
	Reliability	y = P(left) x = label diff	`y ~ x * conditions + (1 + x + conditions|participants)`:Converged
		y = log(firstRT)	`y ~ conditions + (1 + conditions|participants)`:Converged
		y = log(totalRT)	`y ~ conditions + (1 + conditions|participants)`:Converged
		y = points	`y ~ conditions + (1 + conditions|participants)`:Converged
	Volatility	y = P(left) x = label diff	`y ~ x * conditions + (1 + x + conditions|participants)`:Converged
		y = log(firstRT)	`y ~ conditions + (1 + conditions|participants)`:Converged
		y = log(totalRT)	`y ~ conditions + (1 + conditions|participants)`:Converged
		y = points	`y ~ conditions + (1 + conditions|participants)`:Converged
PC depth levelmean	Controllability	y = log(beta)	`y ~ conditions + (1 + conditions|participants)`:Converged
	Reliability	y = log(beta)	`y ~ conditions + (1 + conditions|participants)`:Converged
	Volatility	y = log(beta)	`y ~ conditions + (1 + conditions|participants)`:Converged
PC depth max	Controllability	y = log(beta)	`y ~ conditions + (1 + conditions|participants)`:Converged
	Reliability	y = log(beta)	`y ~ conditions + (1 + conditions|participants)`:Converged
	Volatility	y = log(beta)	`y ~ conditions + (1 + conditions|participants)`:Converged
PC depth path	Controllability	y = log(beta)	`y ~ conditions + (1 + conditions|participants)`:Converged
	Reliability	y = log(beta)	`y ~ conditions + (1 + conditions|participants)`:Converged
	Volatility	y = log(beta)	`y ~ conditions + (1 + conditions|participants)`:Converged
PC depth sum	Controllability	y = log(beta)	`y ~ conditions + (1 + conditions|participants)`:Converged
	Reliability	y = log(beta)	`y ~ conditions + (1 + conditions|participants)`:Converged
	Volatility	y = log(beta)	`y ~ conditions + (1 + conditions|participants)`:Converged

Table S1: Mixed-effects model convergence diagnostics. In all cases, conditions refers to the stochasticity level. Other variables are defined in the column 'Variable'.

All models converged without singular fits. As detailed in the Methods, we used maximal random-effects structures and only simplified if necessary; here, all maximal models converged successfully (Table S1).

6.2 LMM Robustness

Stochasticity	Model	Variable	β (LMM)	β (slope)	P (Wilcoxon)	P (sign)	Same sign
Reliability	Empirical	y = points	−1.16	−1.16	< 0.001	< 0.001	96/100
Reliability	Empirical	y = log(firstRT)	−0.63	−0.63	< 0.001	< 0.001	82/100
Reliability	Empirical	y = log(totalRT)	−0.52	−0.52	< 0.001	< 0.001	74/100
Reliability	PC depth path	$y = \log(\beta)$	−1.86	−1.86	< 0.001	< 0.001	79/100
Reliability	PC depth levelmean	$y = \log(\beta)$	−1.71	−1.71	< 0.001	< 0.001	80/100
Reliability	PC depth max	$y = \log(\beta)$	−1.84	−1.84	< 0.001	< 0.001	81/100
Reliability	PC depth sum	$y = \log(\beta)$	−1.89	−1.89	< 0.001	< 0.001	82/100
Volatility	Empirical	y = points	−1.26	−1.26	< 0.001	< 0.001	97/100
Volatility	Empirical	y = log(firstRT)	−0.49	−0.49	< 0.001	< 0.001	83/100
Volatility	Empirical	y = log(totalRT)	−0.21	−0.21	0.009	0.368	55/100
Volatility	PC depth path	$y = \log(\beta)$	−1.76	−1.76	< 0.001	< 0.001	80/100
Volatility	PC depth levelmean	$y = \log(\beta)$	−1.82	−1.82	< 0.001	< 0.001	81/100
Volatility	PC depth max	$y = \log(\beta)$	−1.87	−1.87	< 0.001	< 0.001	81/100
Volatility	PC depth sum	$y = \log(\beta)$	−1.72	−1.72	< 0.001	< 0.001	79/100
Controllability	Empirical	y = points	−2.54	−2.54	< 0.001	< 0.001	94/100
Controllability	Empirical	y = log(firstRT)	−0.94	−0.94	< 0.001	< 0.001	85/100
Controllability	Empirical	y = log(totalRT)	−0.41	−0.41	0.001	0.007	64/100
Controllability	PC depth path	$y = \log(\beta)$	−3.96	−3.96	< 0.001	< 0.001	91/100
Controllability	PC depth levelmean	$y = \log(\beta)$	−4.04	−4.04	< 0.001	< 0.001	92/100
Controllability	PC depth max	$y = \log(\beta)$	−3.91	−3.91	< 0.001	< 0.001	92/100
Controllability	PC depth sum	$y = \log(\beta)$	−4.01	−4.01	< 0.001	< 0.001	92/100

Table S2: Distribution-agnostic verification of each linear mixed-effects result. β (LMM) is the fitted fixed effect of stochasticity level; β (slope) is the mean per-participant OLS slope of the same outcome on stochasticity level. P values are two-tailed Wilcoxon signed-rank and sign tests on those slopes ($n = 100$ participants per condition; ties conservatively broken). 'Same sign' gives the number of participants whose individual slope matched the direction of the group effect.

Here we examine, where applicable, the normality and equal-variance assumptions underlying our models. Specifically, linear mixed models typically assume a normal distribution of residuals with constant variance. At our scale, with 15,000–105,000 residuals per trial-level model, normality or equal-variance tests would reject departures that are too small to directly affect inference. Rather than directly test for normality, we show that relaxing these normality assumptions does not change our findings.

The residuals depart moderately from normality in expected ways. Points earned per trial is mildly left-skewed and platykurtic, consistent with the fact that the number of points earned is bounded at a maximum and minimum level by the range of possible point values (1 to 9, inclusive). Response time in seconds is highly right-skewed, which justifies our choice of using the log-transform; even after transforming, it retains a modest rightward tail but much improved skew. Conditional log-β, our recovered model-fitted parameter, is mildly right-skewed. Residual variance is close to constant across stochasticity levels for every model, the largest ratio between highest- and lowest-variance level being 1.47.

To test robustness to normality, we recomputed each effect without invoking the normality assumption. For each participant and each dependent variable, we regressed the mean value of the dependent variable on each stochasticity level, giving one slope per participant, and tested each of the slopes with a Wilcoxon signed-rank test and a

sign test. Both make the participant the unit of analysis and neither assumes a normal distribution of residuals; we conservatively broke ties in favor of non-significance. For each effect, we reproduced the same direction and approximate magnitude (Table S2): the participant slopes recover the fitted coefficients to the precision reported, and both tests are significant for the main analysis. One exception occurs in the supplementary analyses: total RT versus stochasticity level, under volatility only, was not significant under the sign test, though the Wilcoxon test remained significant. This is unsurprising given that the total RT aggregates over many moves that may be reflective of motor delay rather than planning time, thereby reducing the significance of the effect on thinking time. The generalized linear mixed models are binomial and don't require the normality assumption, so they are not included here.

Supplementary Note 7: Model and parameter recovery

7.1 Simulation procedure

We conducted simulations using synthetic data to test whether our fitting procedures could accurately recover both the true model and the underlying parameters.

Synthetic participant parameters were drawn randomly: depth $d \in \{1, \dots, 7\}$, rank/value $k, v \in \{1, \dots, 9\}$; lapse $\lambda \sim U[10^{-3}, 0.5]$; and $\log(\beta) \sim U[-2, 0.5]$ (Policy Compression models: one $\log(\beta_q)$ per stochasticity level; Filter Adaptation models: a single global $\log(\beta)$), spanning the range observed in the empirical data. To ensure a realistic recovery regime, we generated exactly 100 participants for each generating model and stochasticity variant, with the same per-variant trial structure as in the experiment: 7 decisions per game, 30 games per stochasticity level, across 5 stochasticity levels.

Fitted continuous parameters used the same bounds and multi-start procedure as in Methods ($-3 \leq \log\beta \leq 3$, $10^{-3} \leq \lambda \leq 1$; 50 random restarts per continuous-parameter fit).

7.2 Model recovery

Model recovery is a common technique used to test model identifiability. In our case, several models provided comparably good fits across reliability, volatility, and controllability. For instance, the PC Depth-filtering Path and Level-Mean models were best fitting in all three forms of stochasticity, while the PC Depth-filtering Sum and Max models were also competitive under volatility.

We then fitted the synthetic data using our model fitting procedure to see whether the ground truth models were recoverable. Consistent with our model fitting procedure for our real data, we conducted five-fold cross-validation to establish the best-fitting model for each synthetic dataset, using the cross-validated negative log likelihood (sum of held-out fold NLLs) as our measure of goodness-of-fit. A model was said to be recoverable when bootstrap distributions of ΔCV NLL (each alternative minus the generating baseline, resampling the 100 simulated participants 10^6 times) showed that the generating model was substantially favored (using 95% confidence intervals) relative to all alternatives.

We find that under all conditions, for all models, the best-fitting model corresponded with the model that actually generated the synthetic dataset, demonstrating that our models are both distinguishable and recoverable from simulated data (Fig S8 - S37).

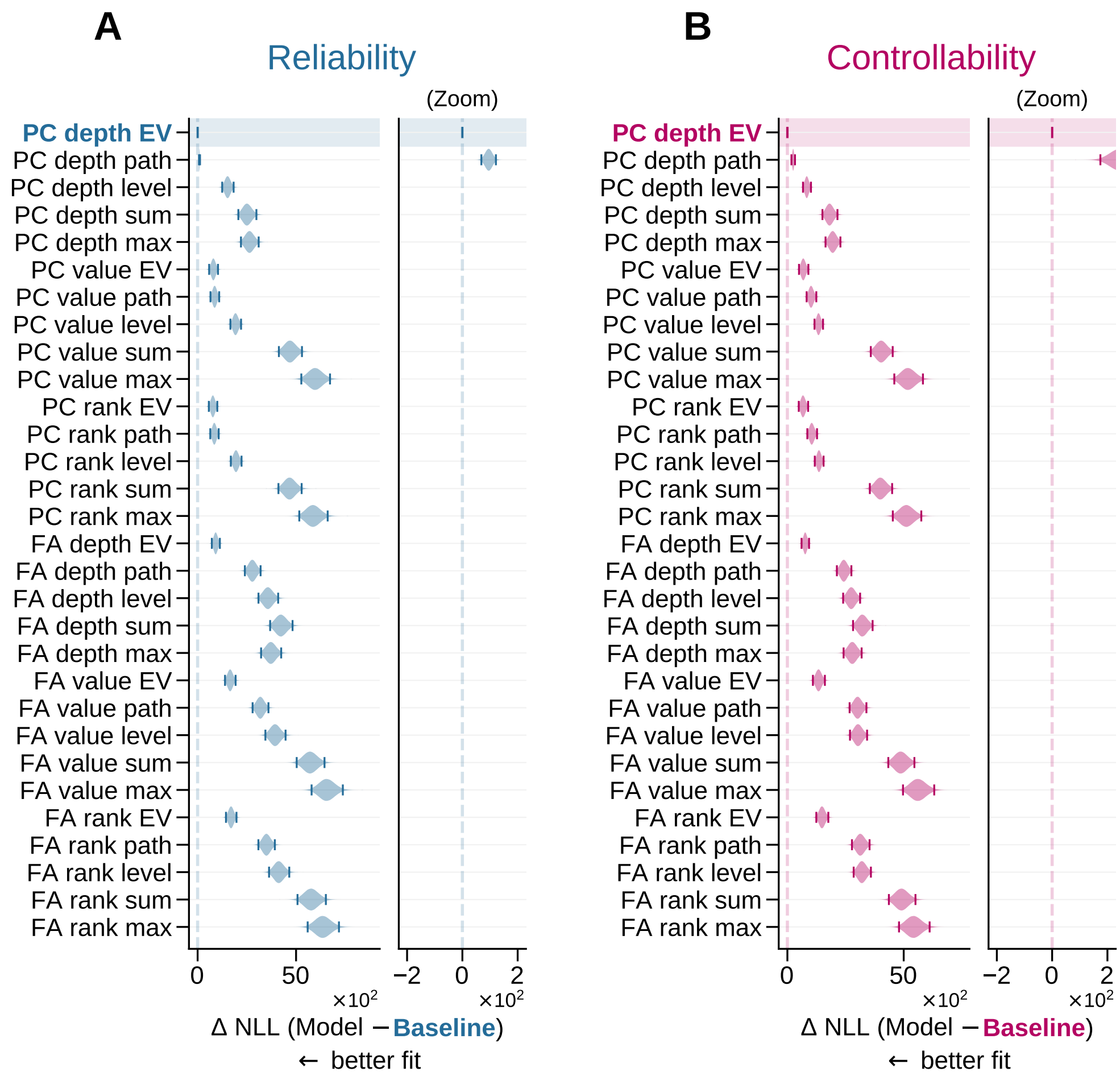

Fig. S8: Model Recovery based on Simulated Data from the PC Depth-filtering EV (baseline) model. Bootstrapped differences (n = 100 simulated participants per condition, 10^6 resamples) between the 5-fold cross-validated negative log likelihoods of alternative models and the baseline model. Bars represent mean ± 95% CI of the bootstrap distribution. Each panel includes a zoomed-in inset to the right. **A.** Reliability. **B.** Controllability.

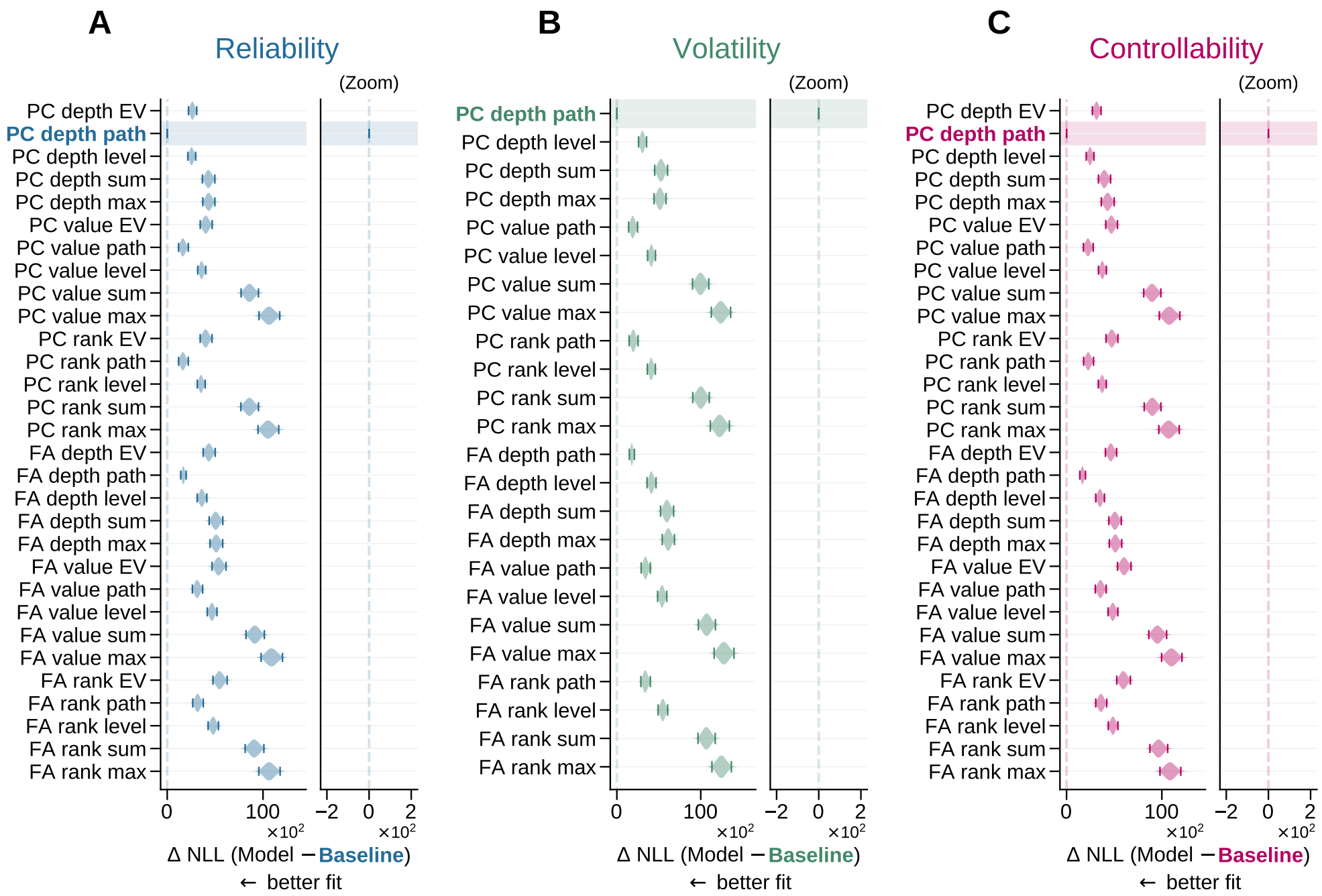

Fig. S9: Model Recovery based on Simulated Data from the PC Depth-filtering Path (baseline) model. Bootstrapped differences ($n = 100$ simulated participants per condition, 10^6 resamples) between the 5-fold cross-validated negative log likelihoods of alternative models and the baseline model. Bars represent mean ± 95% CI of the bootstrap distribution. Each panel includes a zoomed-in inset to the right. **A.** Reliability. **B.** Volatility. **C.** Controllability.

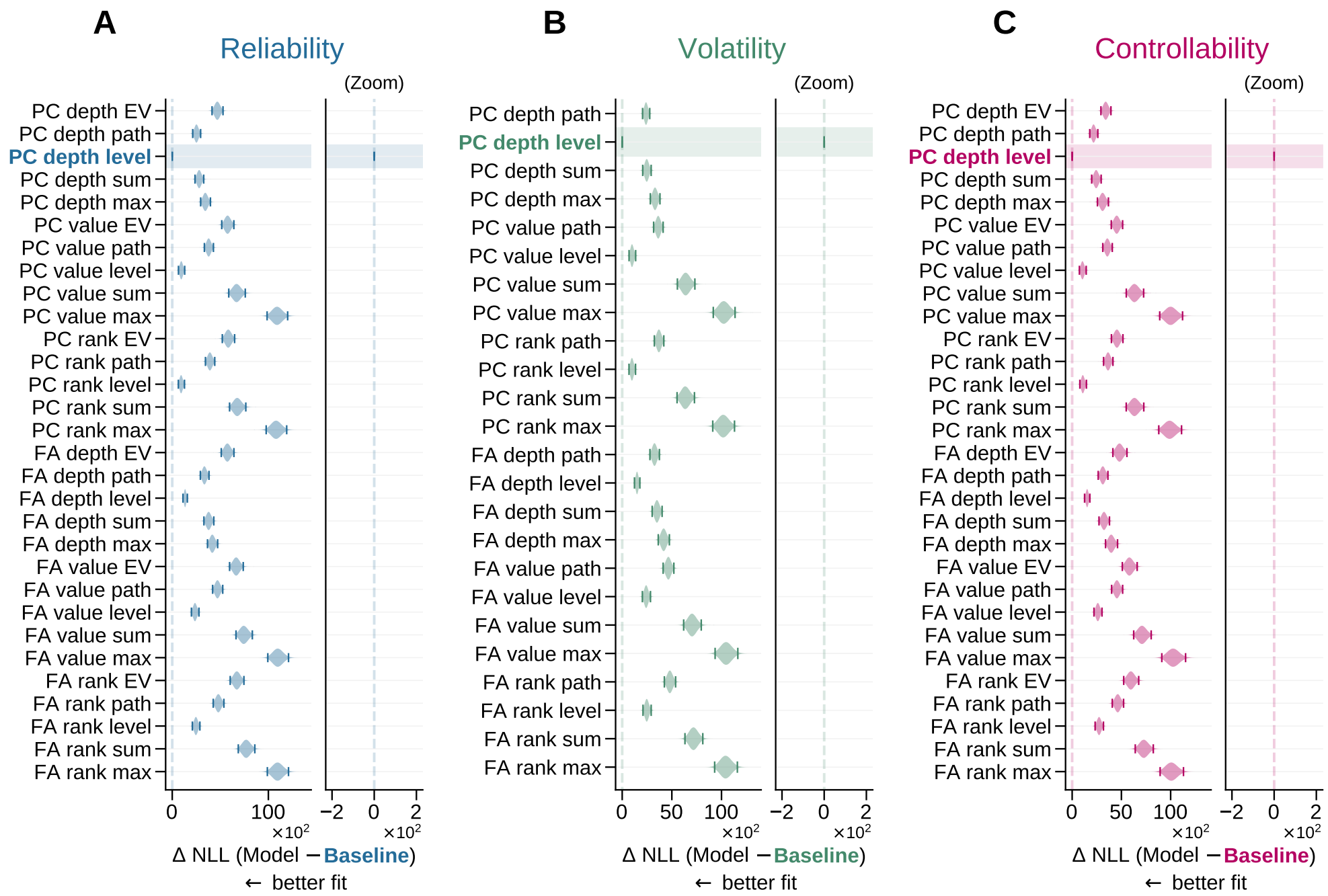

Fig. S10: Model Recovery based on Simulated Data from the PC Depth-filtering Level-Mean (baseline) model. Bootstrapped differences ($n = 100$ simulated participants per condition, 10^6 resamples) between the 5-fold cross-validated negative log likelihoods of alternative models and the baseline model. Bars represent mean ± 95% CI of the bootstrap distribution. Each panel includes a zoomed-in inset to the right. **A.** Reliability. **B.** Volatility. **C.** Controllability.

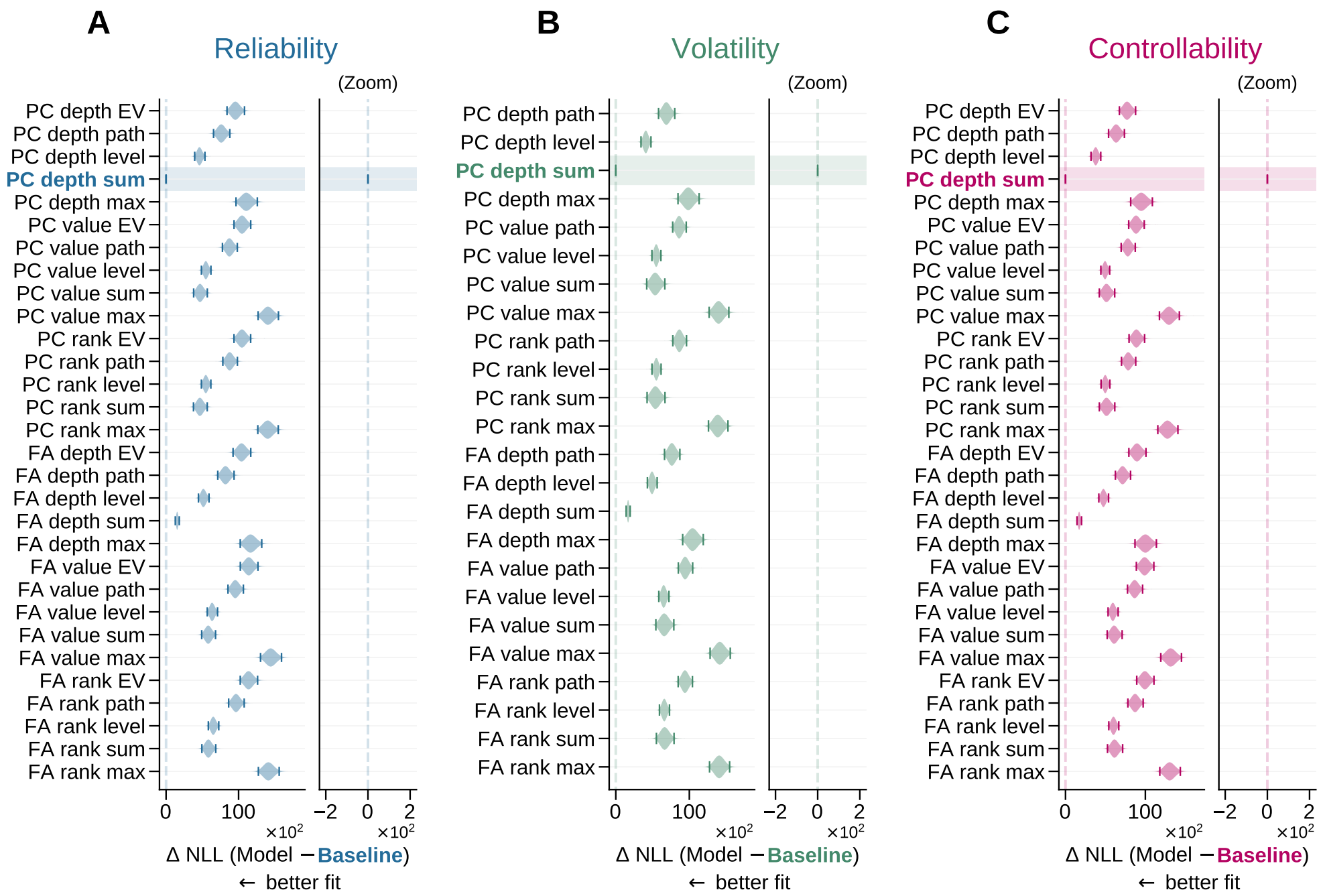

Fig. S11: Model Recovery based on Simulated Data from the PC Depth-filtering Sum (baseline) model. Bootstrapped differences (n = 100 simulated participants per condition, 10^6 resamples) between the 5-fold cross-validated negative log likelihoods of alternative models and the baseline model. Bars represent mean ± 95% CI of the bootstrap distribution. Each panel includes a zoomed-in inset to the right. **A.** Reliability. **B.** Volatility. **C.** Controllability.

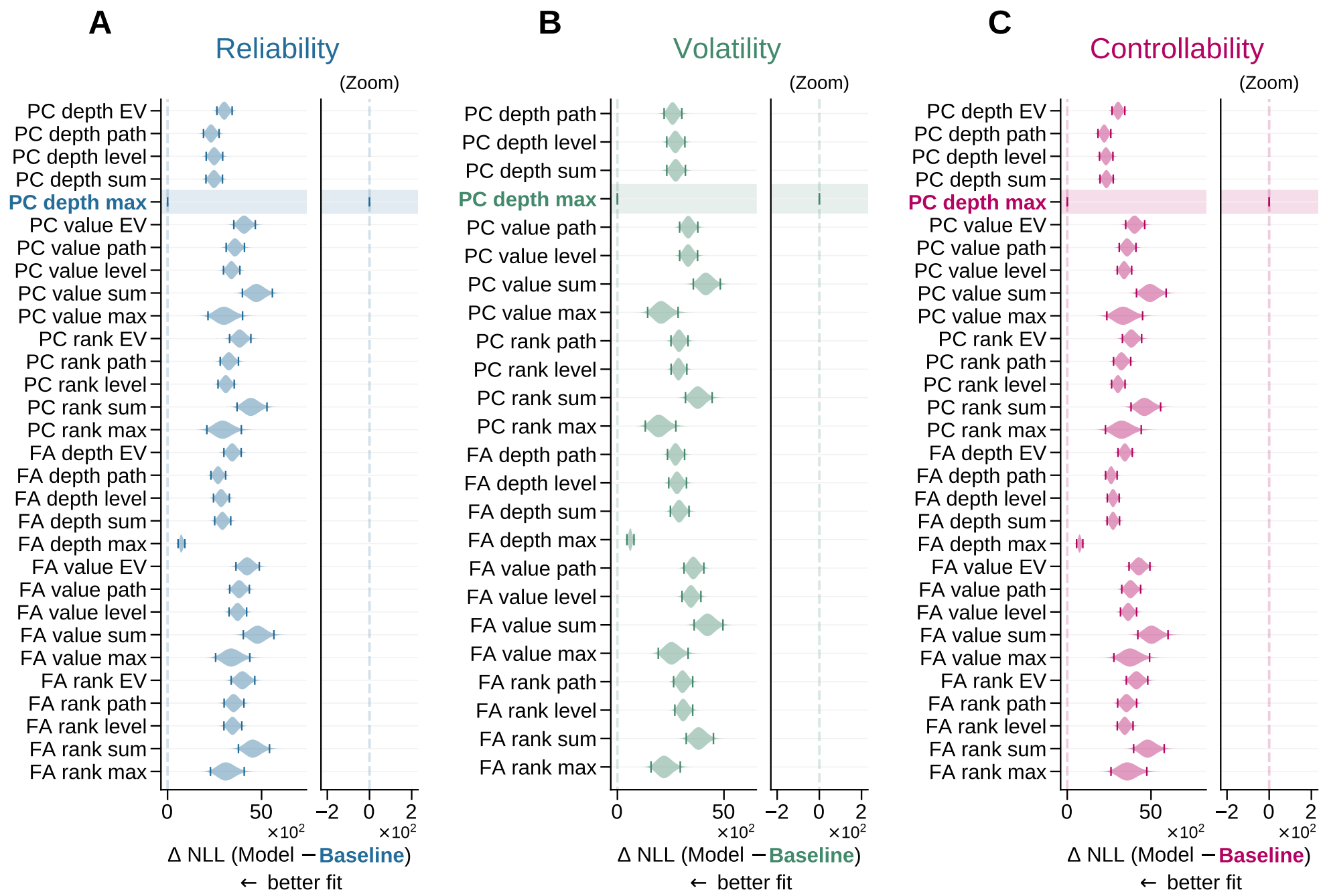

Fig. S12: Model Recovery based on Simulated Data from the PC Depth-filtering Max (baseline) model. Bootstrapped differences (n = 100 simulated participants per condition, 10^6 resamples) between the 5-fold cross-validated negative log likelihoods of alternative models and the baseline model. Bars represent mean ± 95% CI of the bootstrap distribution. Each panel includes a zoomed-in inset to the right. **A.** Reliability. **B.** Volatility. **C.** Controllability.

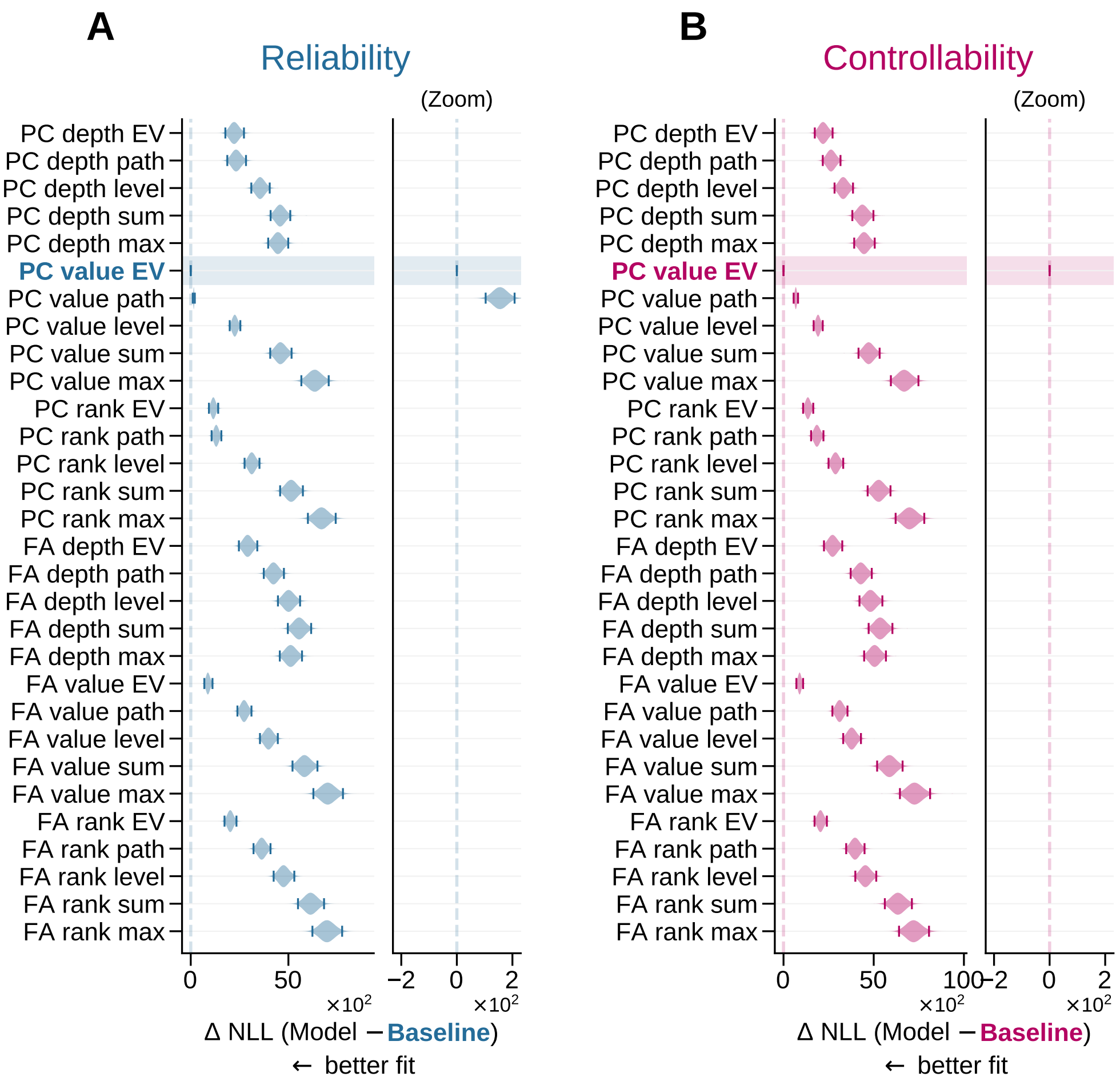

Fig. S13: Model Recovery based on Simulated Data from the PC Value-filtering EV (baseline) model. Bootstrapped differences (n = 100 simulated participants per condition, 10^6 resamples) between the 5-fold cross-validated negative log likelihoods of alternative models and the baseline model. Bars represent mean ± 95% CI of the bootstrap distribution. Each panel includes a zoomed-in inset to the right. **A.** Reliability. **B.** Controllability.

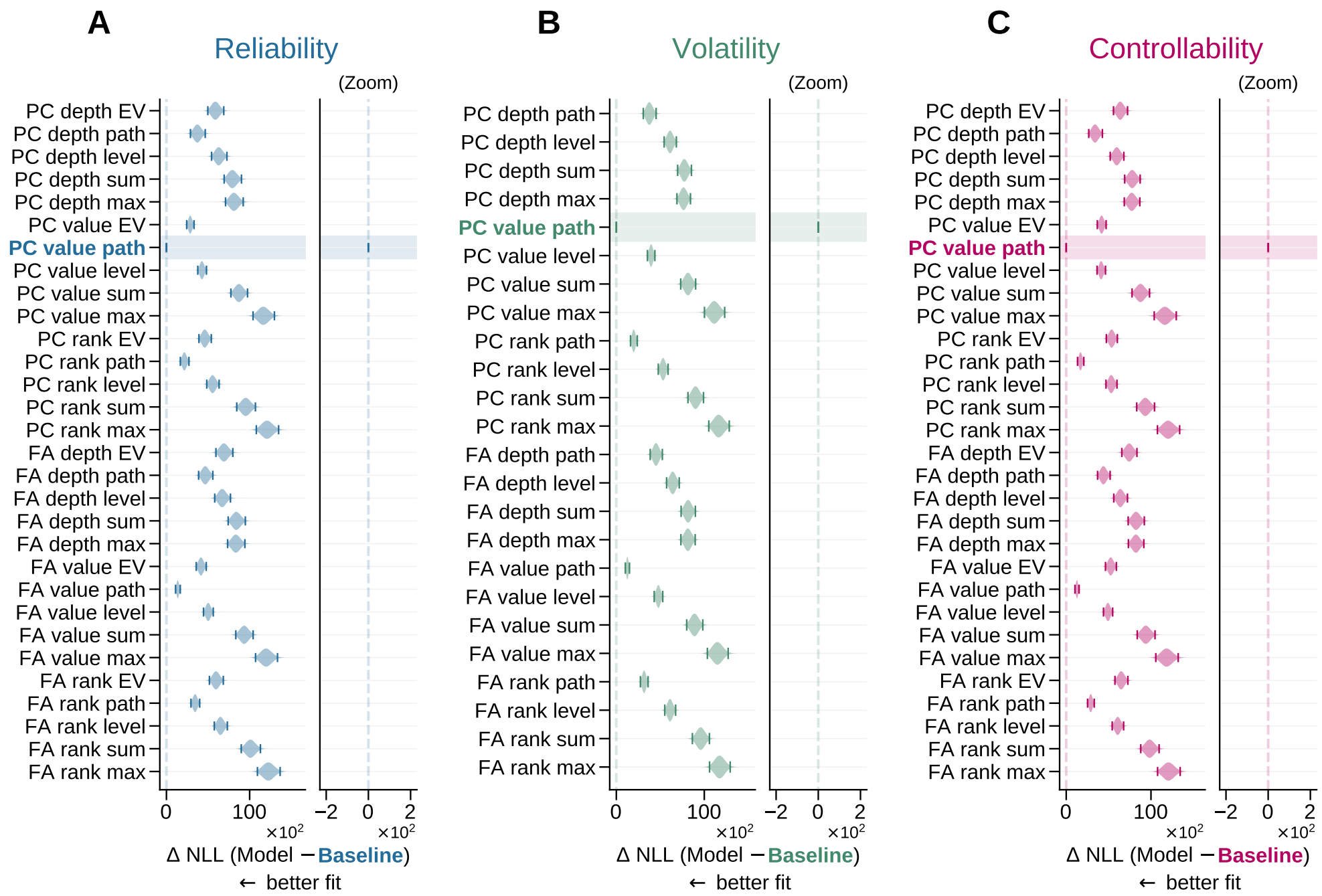

Fig. S14: Model Recovery based on Simulated Data from the PC Value-filtering Path (baseline) model. Bootstrapped differences ($n = 100$ simulated participants per condition, 10^6 resamples) between the 5-fold cross-validated negative log likelihoods of alternative models and the baseline model. Bars represent mean ± 95% CI of the bootstrap distribution. Each panel includes a zoomed-in inset to the right. **A.** Reliability. **B.** Volatility. **C.** Controllability.

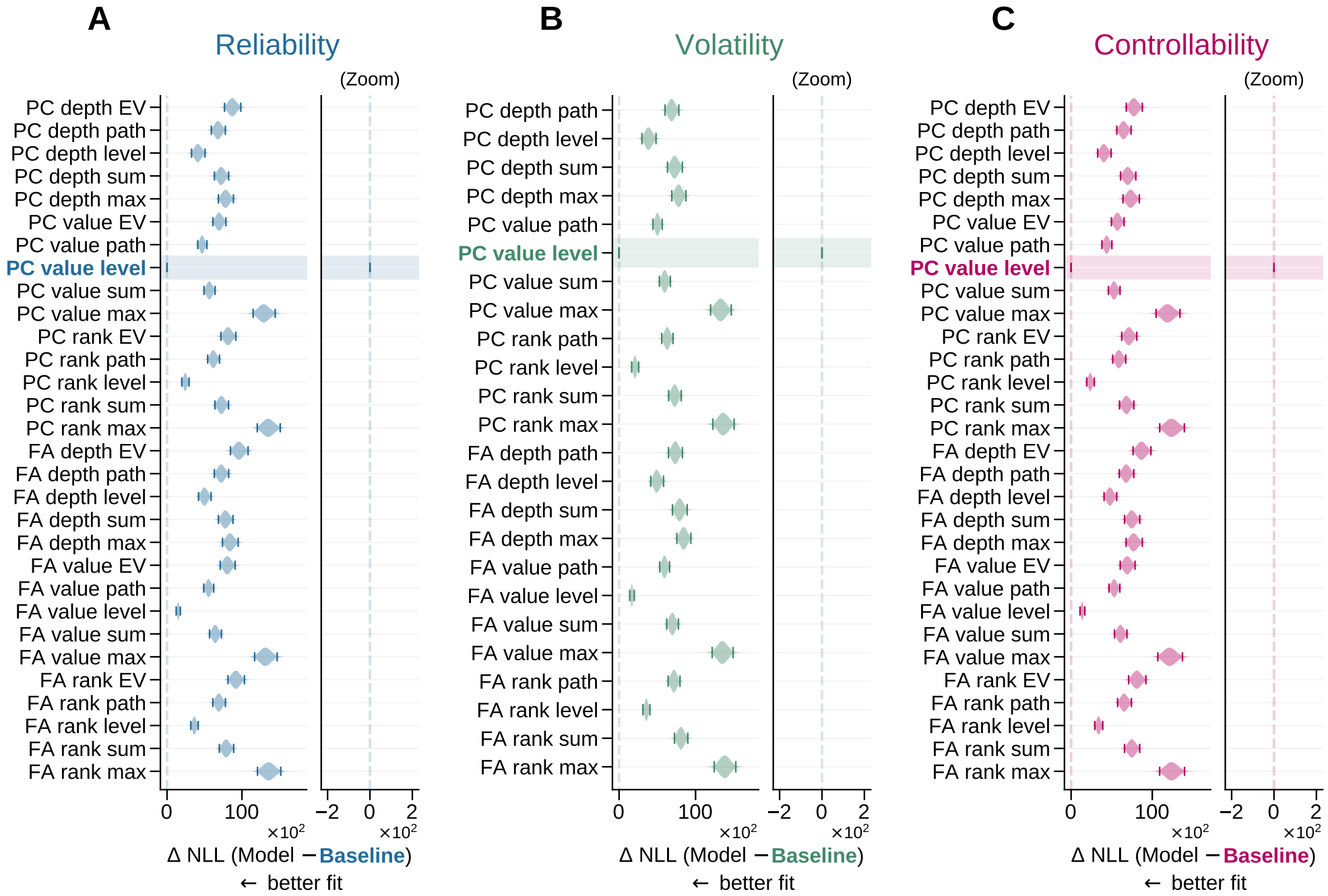

Fig. S15: Model Recovery based on Simulated Data from the PC Value-filtering Level-Mean (baseline) model. Bootstrapped differences ($n = 100$ simulated participants per condition, 10^6 resamples) between the 5-fold cross-validated negative log likelihoods of alternative models and the baseline model. Bars represent mean ± 95% CI of the bootstrap distribution. Each panel includes a zoomed-in inset to the right. **A.** Reliability. **B.** Volatility. **C.** Controllability.

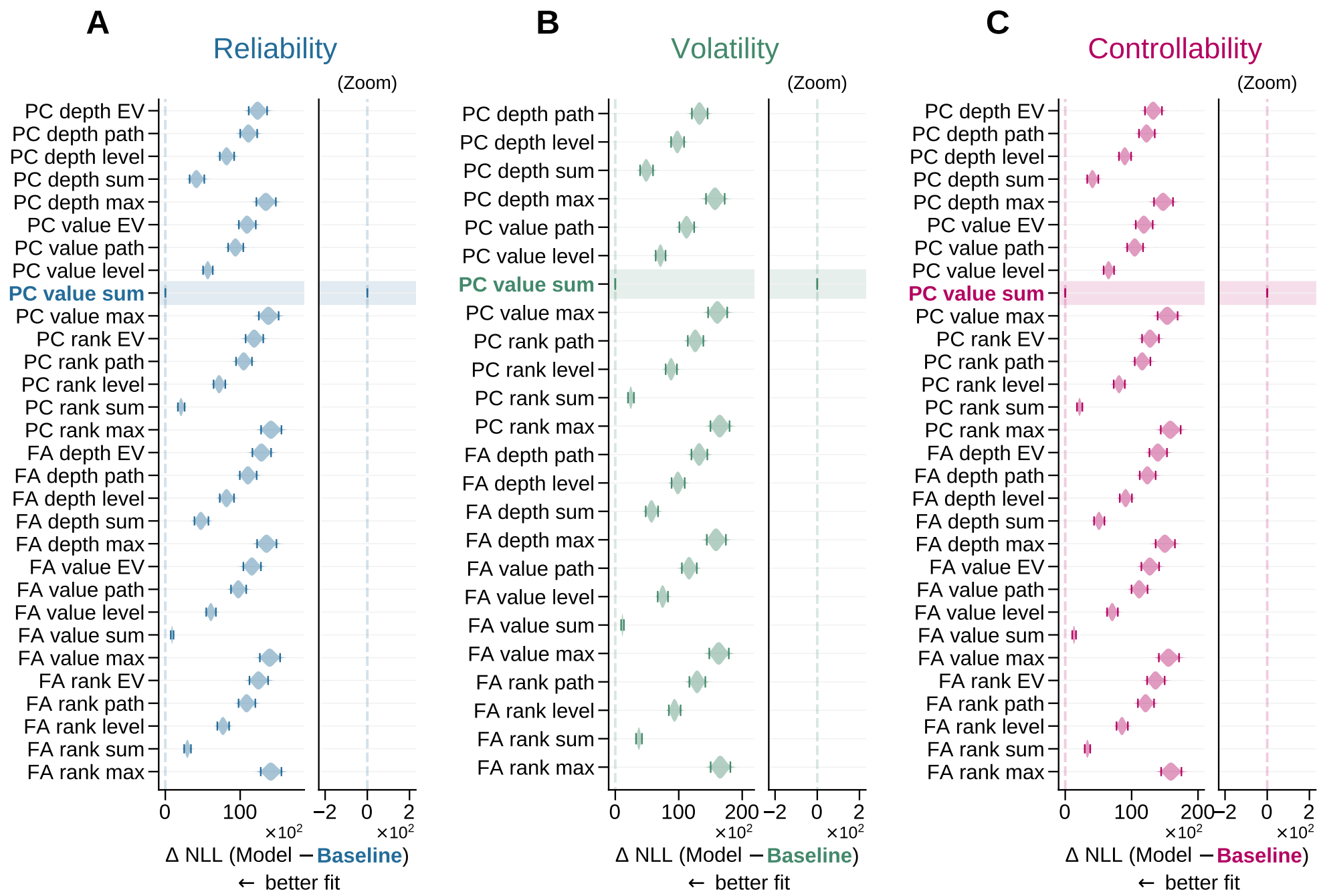

Fig. S16: Model Recovery based on Simulated Data from the PC Value-filtering Sum (baseline) model. Bootstrapped differences ($n = 100$ simulated participants per condition, 10^6 resamples) between the 5-fold cross-validated negative log likelihoods of alternative models and the baseline model. Bars represent mean ± 95% CI of the bootstrap distribution. Each panel includes a zoomed-in inset to the right. **A.** Reliability. **B.** Volatility. **C.** Controllability.

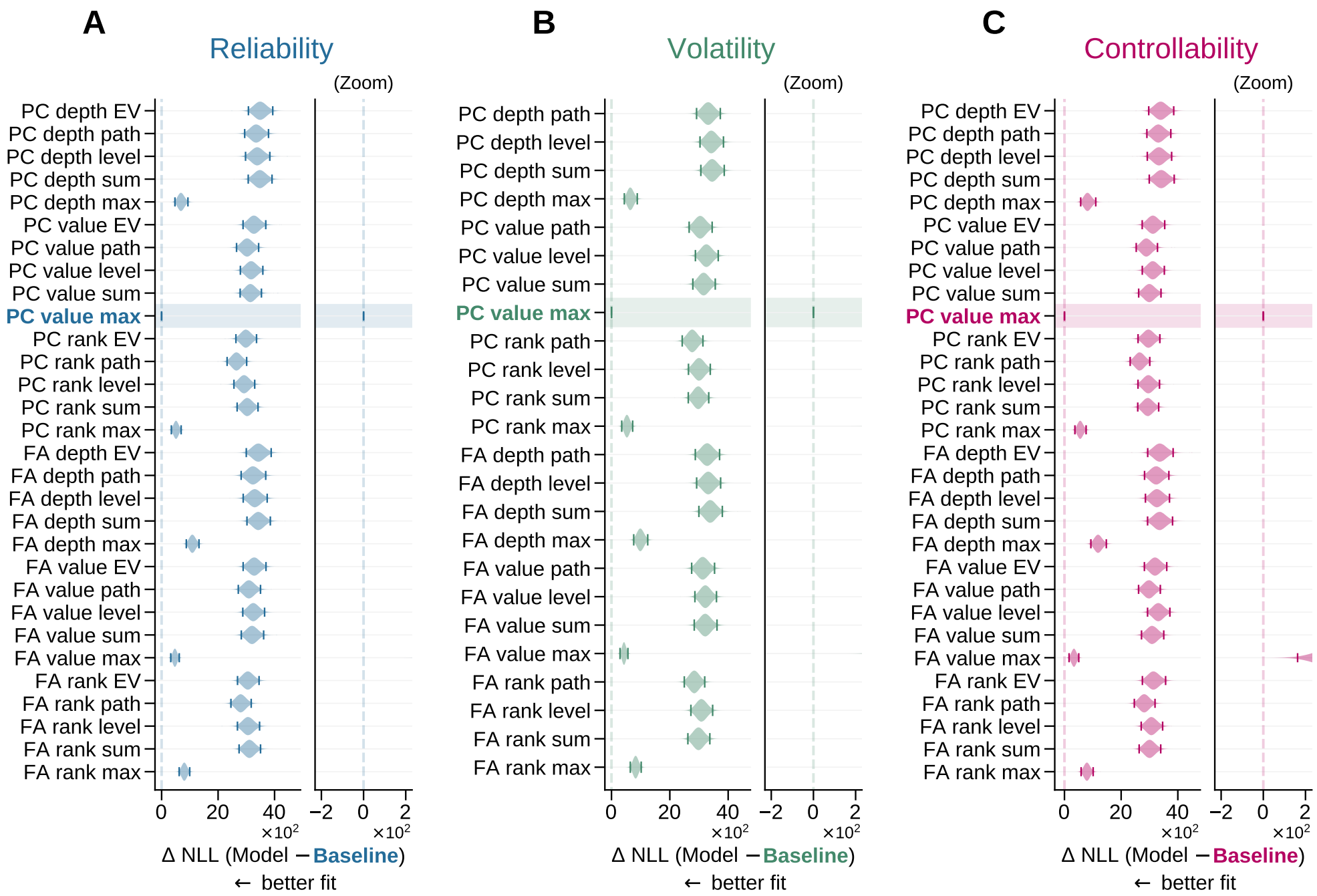

Fig. S17: Model Recovery based on Simulated Data from the PC Value-filtering Max (baseline) model. Bootstrapped differences ($n = 100$ simulated participants per condition, 10^6 resamples) between the 5-fold cross-validated negative log likelihoods of alternative models and the baseline model. Bars represent mean ± 95% CI of the bootstrap distribution. Each panel includes a zoomed-in inset to the right. **A.** Reliability. **B.** Volatility. **C.** Controllability.

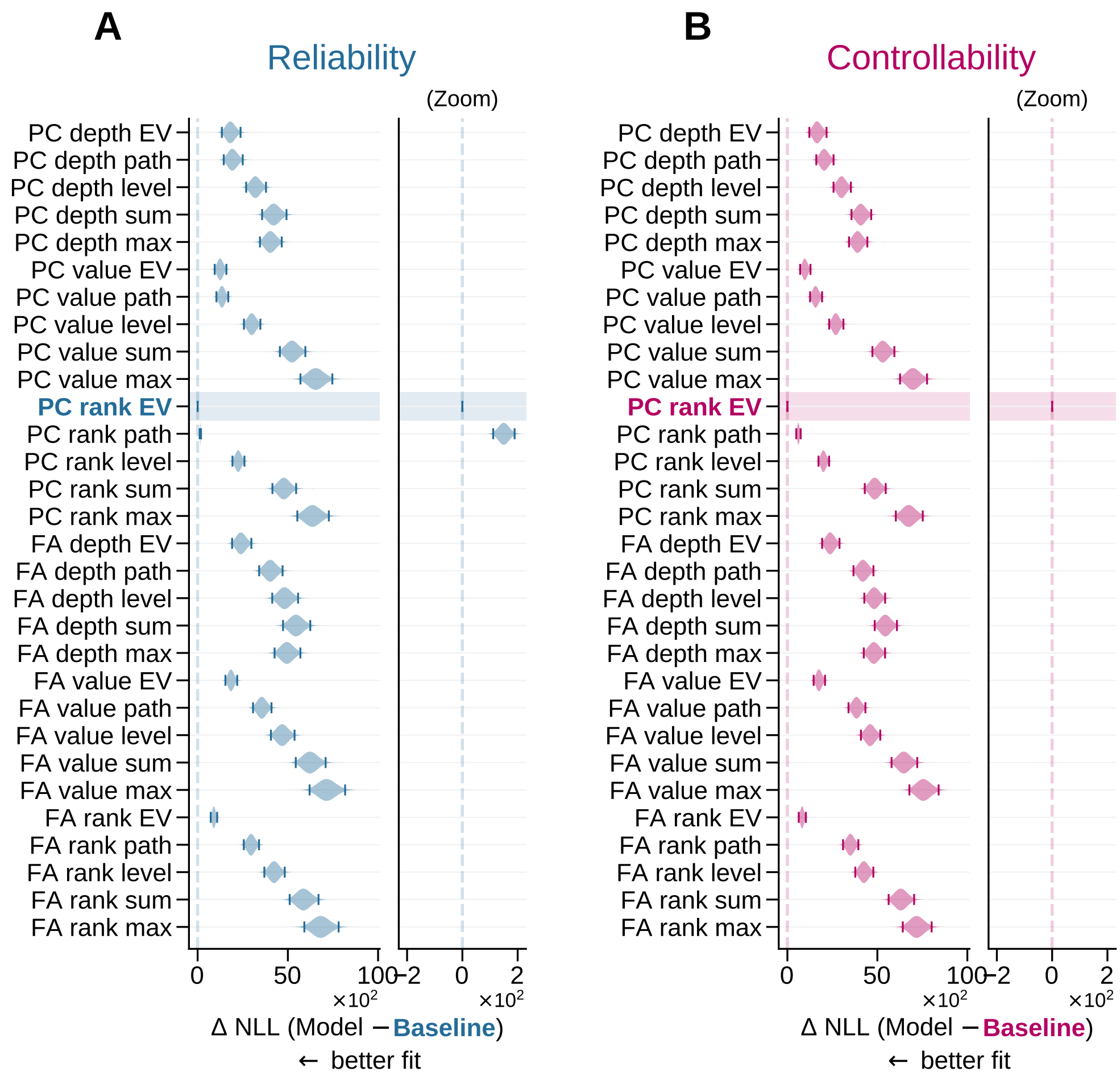

Fig. S18: Model Recovery based on Simulated Data from the PC Rank-filtering EV (baseline) model. Bootstrapped differences (n = 100 simulated participants per condition, 10^6 resamples) between the 5-fold cross-validated negative log likelihoods of alternative models and the baseline model. Bars represent mean ± 95% CI of the bootstrap distribution. Each panel includes a zoomed-in inset to the right. **A.** Reliability. **B.** Controllability.

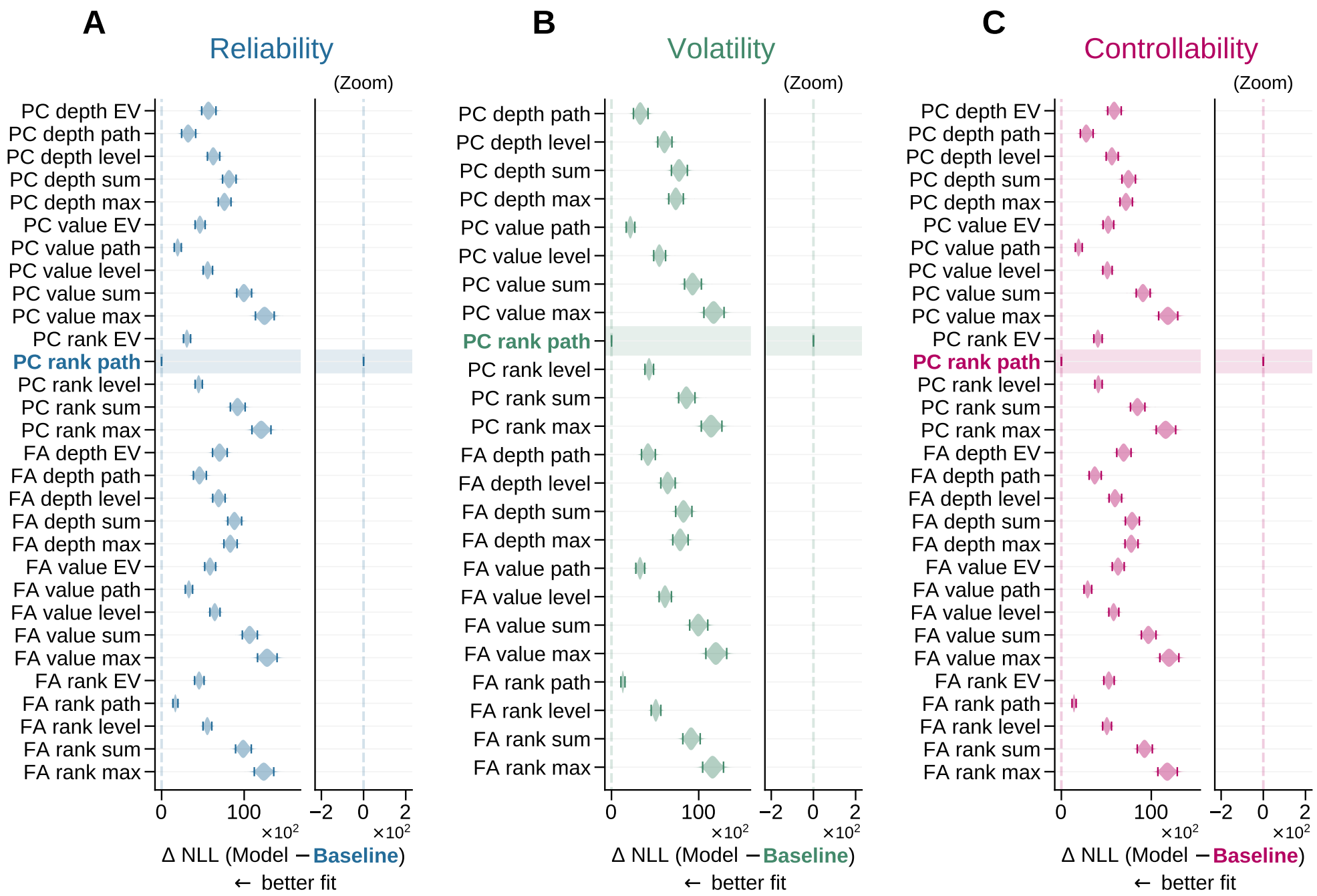

Fig. S19: Model Recovery based on Simulated Data from the PC Rank-filtering Path (baseline) model. Bootstrapped differences (n = 100 simulated participants per condition, 10^6 resamples) between the 5-fold cross-validated negative log likelihoods of alternative models and the baseline model. Bars represent mean ± 95% CI of the bootstrap distribution. Each panel includes a zoomed-in inset to the right. **A.** Reliability. **B.** Volatility. **C.** Controllability.

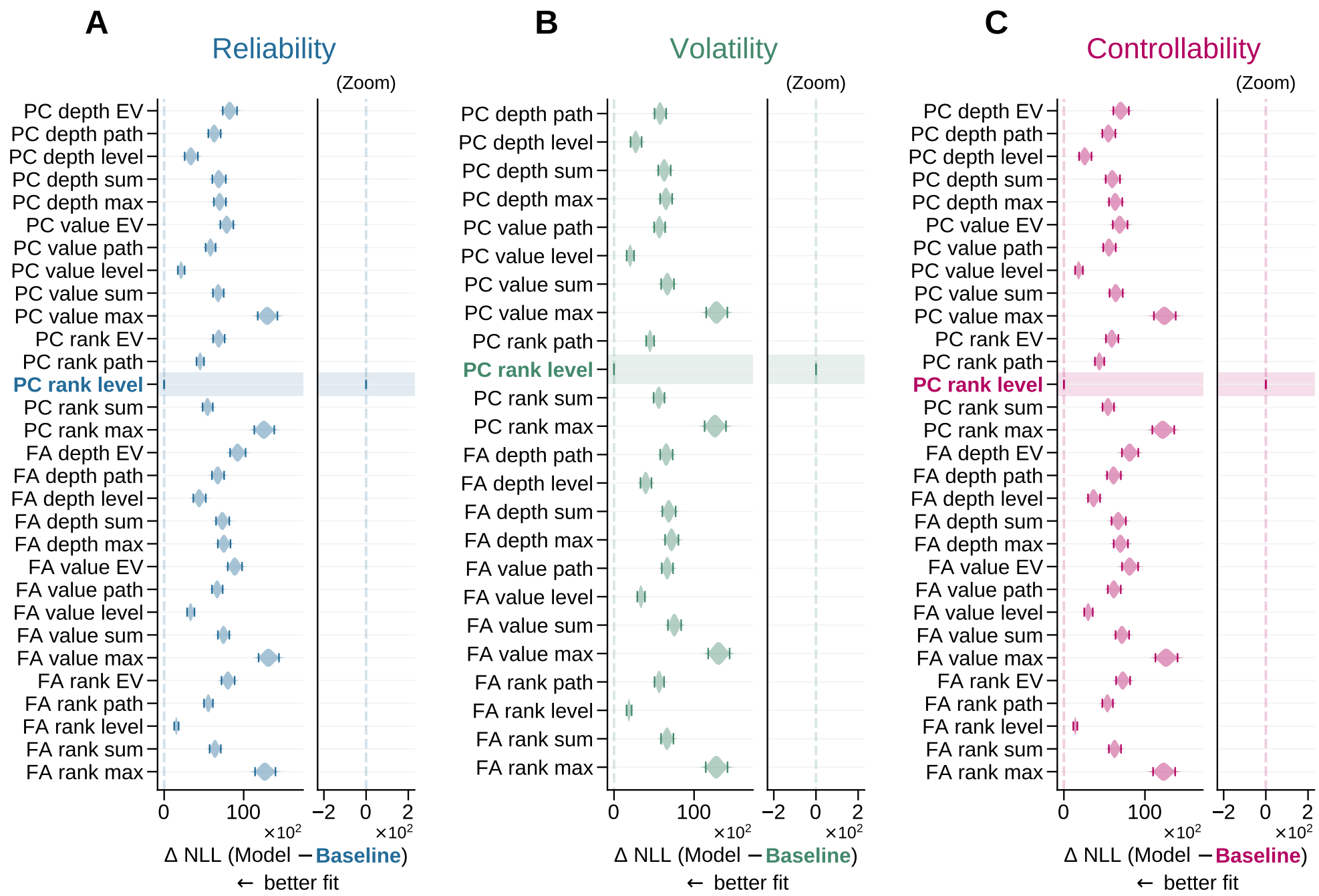

Fig. S20: Model Recovery based on Simulated Data from the PC Rank-filtering Level-Mean (baseline) model. Bootstrapped differences ($n = 100$ simulated participants per condition, 10^6 resamples) between the 5-fold cross-validated negative log likelihoods of alternative models and the baseline model. Bars represent mean ± 95% CI of the bootstrap distribution. Each panel includes a zoomed-in inset to the right. **A.** Reliability. **B.** Volatility. **C.** Controllability.

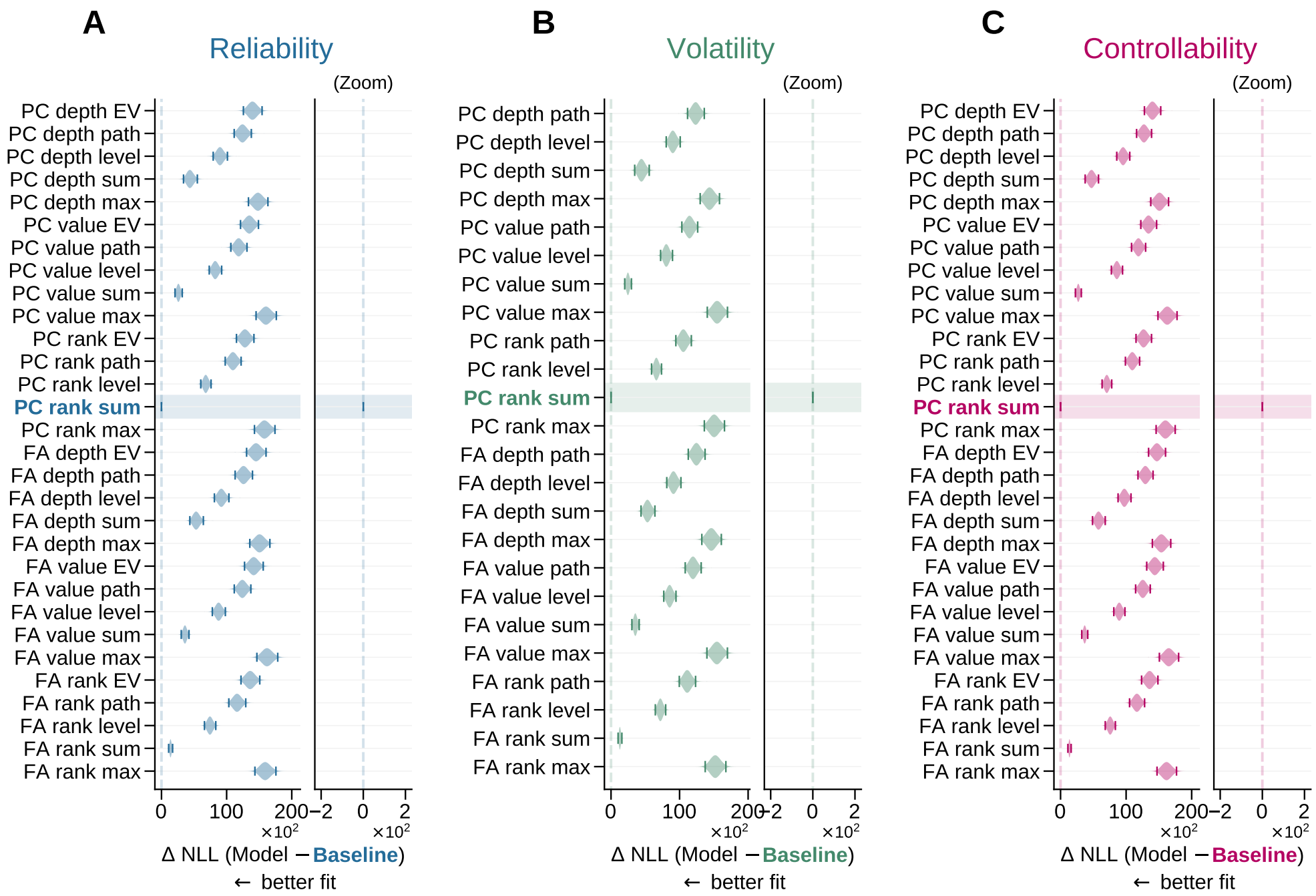

Fig. S21: Model Recovery based on Simulated Data from the PC Rank-filtering Sum (baseline) model. Bootstrapped differences ($n = 100$ simulated participants per condition, 10^6 resamples) between the 5-fold cross-validated negative log likelihoods of alternative models and the baseline model. Bars represent mean ± 95% CI of the bootstrap distribution. Each panel includes a zoomed-in inset to the right. **A.** Reliability. **B.** Volatility. **C.** Controllability.

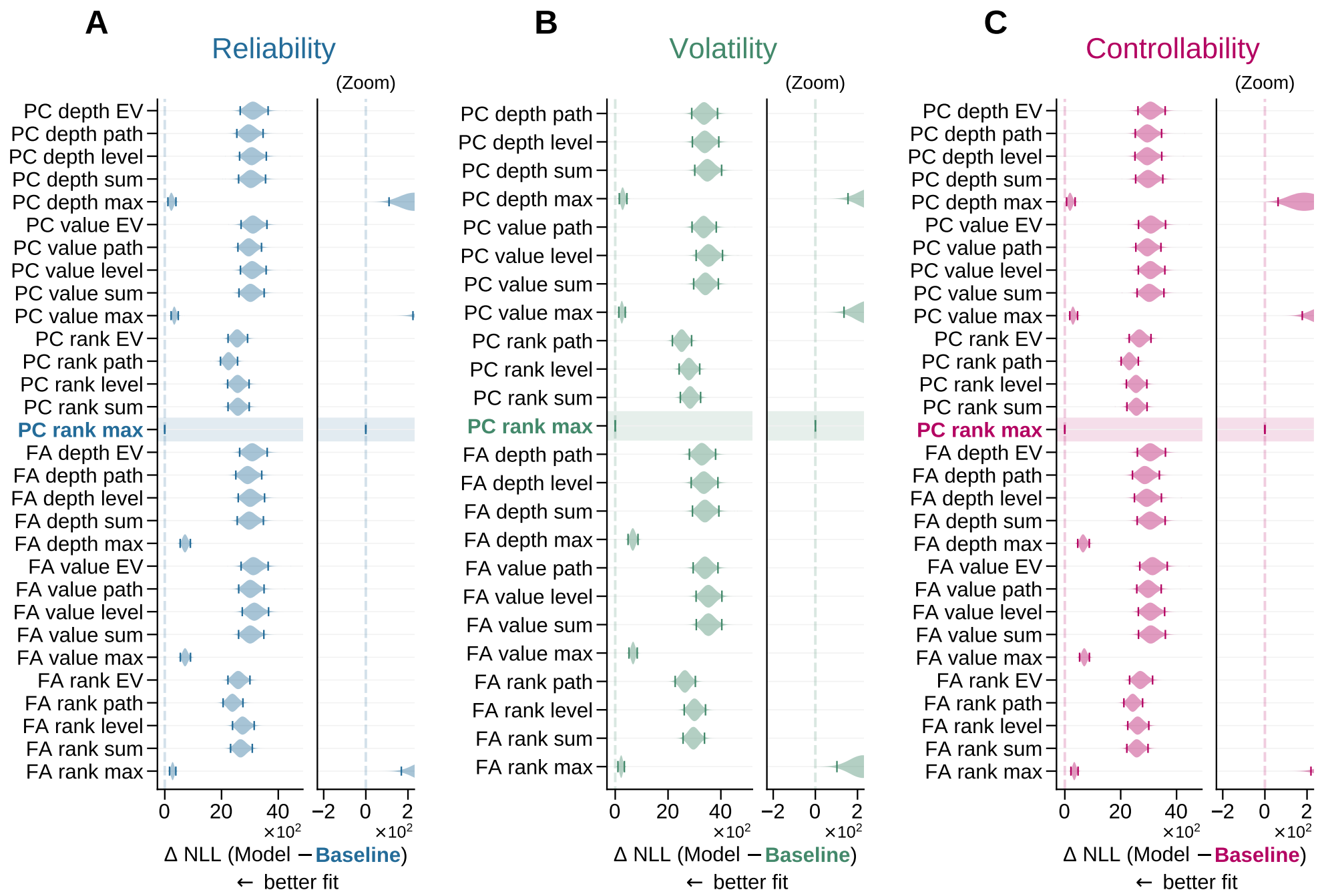

Fig. S22: Model Recovery based on Simulated Data from the PC Rank-filtering Max (baseline) model. Bootstrapped differences (n = 100 simulated participants per condition, 10^6 resamples) between the 5-fold cross-validated negative log likelihoods of alternative models and the baseline model. Bars represent mean ± 95% CI of the bootstrap distribution. Each panel includes a zoomed-in inset to the right. **A.** Reliability. **B.** Volatility. **C.** Controllability.

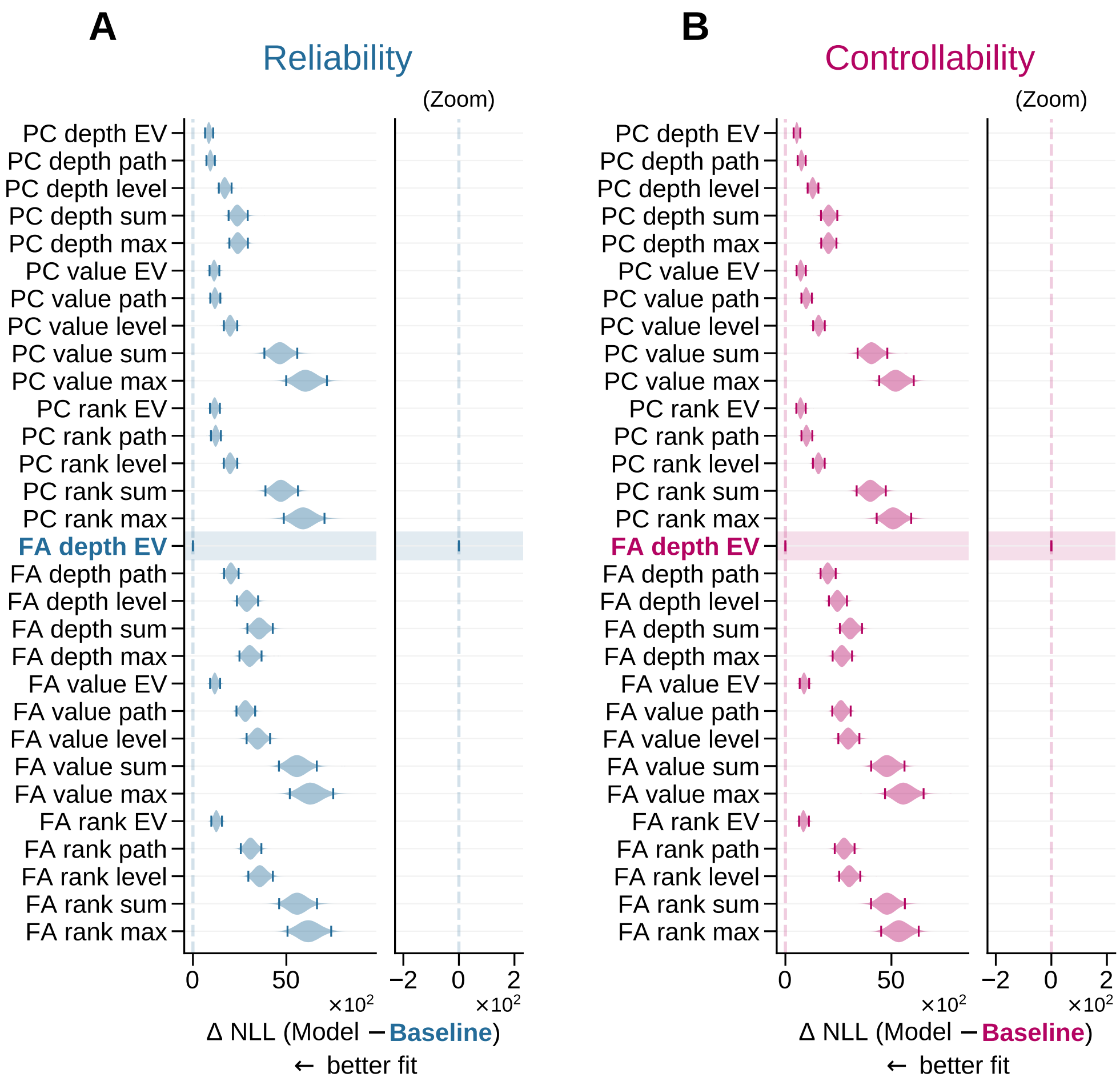

Fig. S23: Model Recovery based on Simulated Data from the FA Depth-filtering EV (baseline) model. Bootstrapped differences (n = 100 simulated participants per condition, 10^6 resamples) between the 5-fold cross-validated negative log likelihoods of alternative models and the baseline model. Bars represent mean ± 95% CI of the bootstrap distribution. Each panel includes a zoomed-in inset to the right. **A.** Reliability. **B.** Controllability.

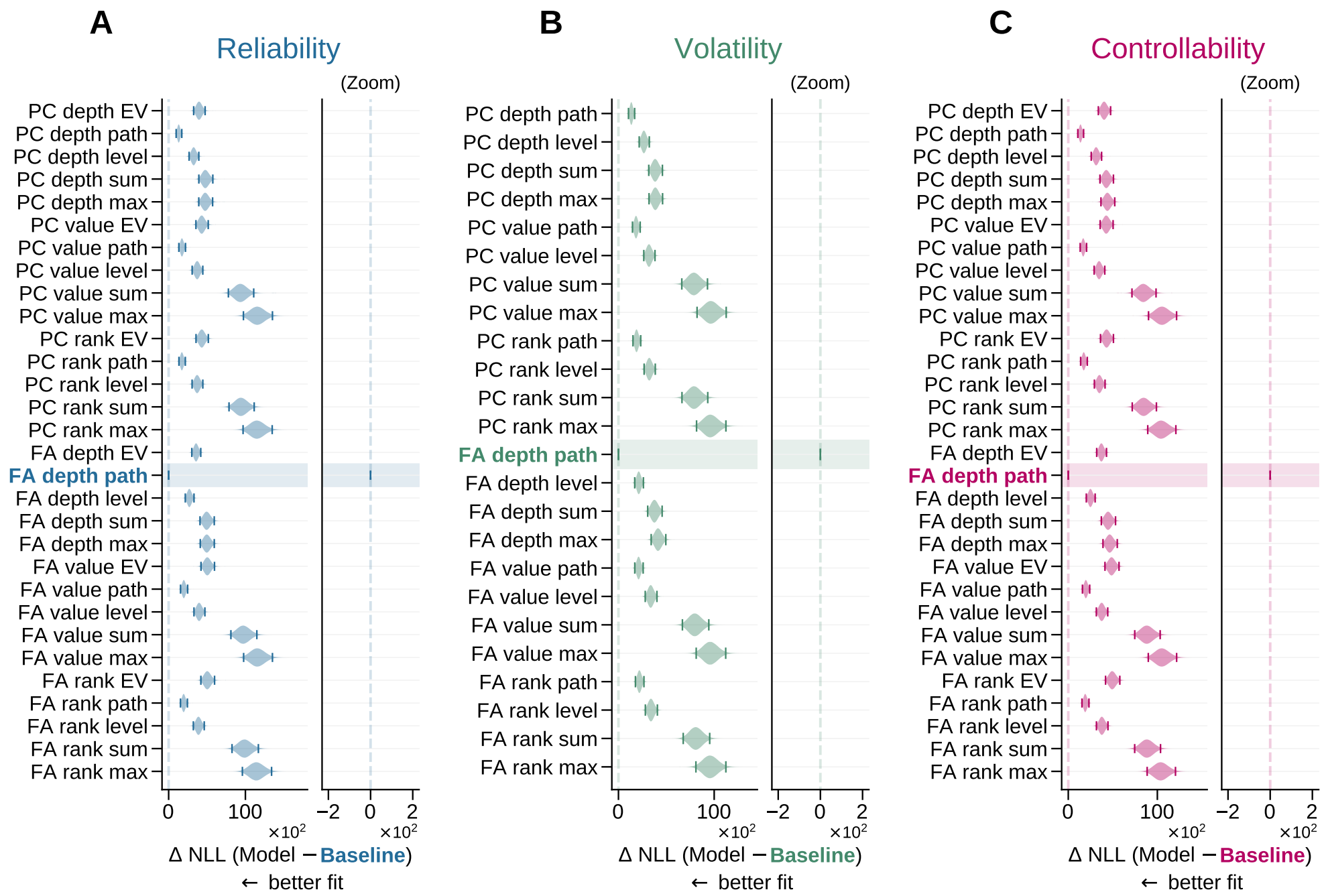

Fig. S24: Model Recovery based on Simulated Data from the FA Depth-filtering Path (baseline) model. Bootstrapped differences (n = 100 simulated participants per condition, 10^6 resamples) between the 5-fold cross-validated negative log likelihoods of alternative models and the baseline model. Bars represent mean ± 95% CI of the bootstrap distribution. Each panel includes a zoomed-in inset to the right. **A.** Reliability. **B.** Volatility. **C.** Controllability.

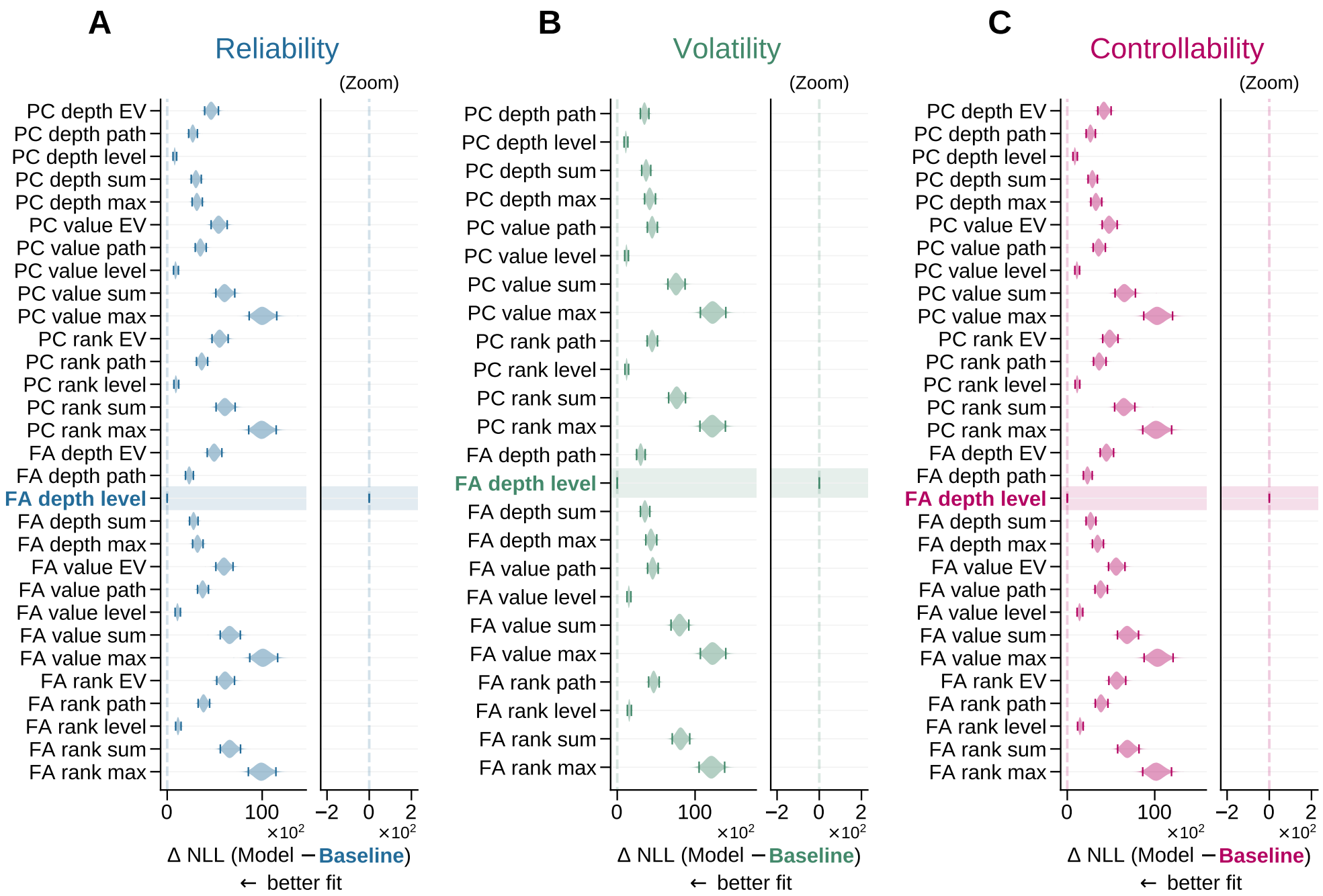

Fig. S25: Model Recovery based on Simulated Data from the FA Depth-filtering Level-Mean (baseline) model. Bootstrapped differences (n = 100 simulated participants per condition, 10^6 resamples) between the 5-fold cross-validated negative log likelihoods of alternative models and the baseline model. Bars represent mean ± 95% CI of the bootstrap distribution. Each panel includes a zoomed-in inset to the right. **A.** Reliability. **B.** Volatility. **C.** Controllability.

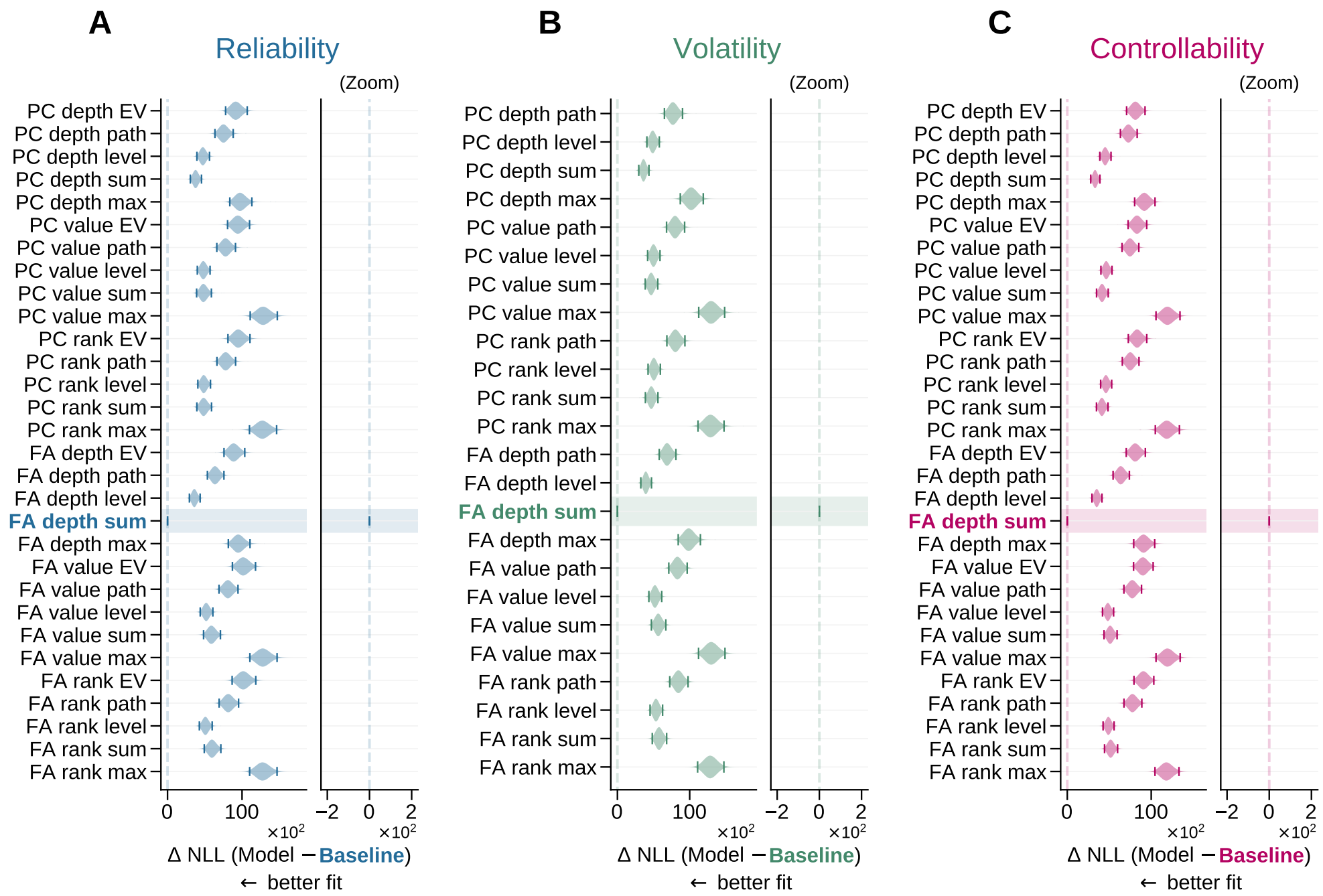

Fig. S26: Model Recovery based on Simulated Data from the FA Depth-filtering Sum (baseline) model. Bootstrapped differences (n = 100 simulated participants per condition, 10^6 resamples) between the 5-fold cross-validated negative log likelihoods of alternative models and the baseline model. Bars represent mean ± 95% CI of the bootstrap distribution. Each panel includes a zoomed-in inset to the right. **A.** Reliability. **B.** Volatility. **C.** Controllability.

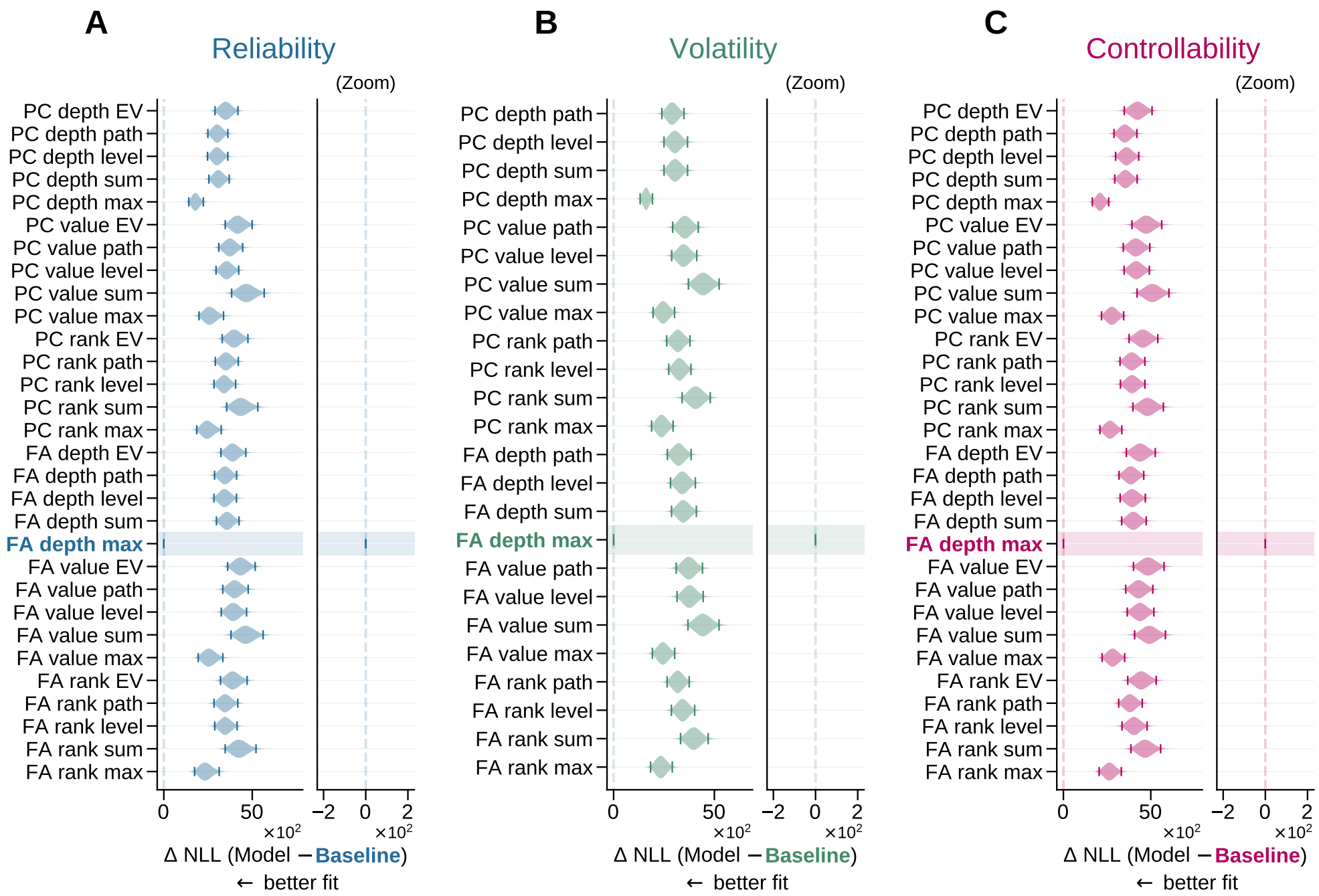

Fig. S27: Model Recovery based on Simulated Data from the FA Depth-filtering Max (baseline) model. Bootstrapped differences ($n = 100$ simulated participants per condition, 10^6 resamples) between the 5-fold cross-validated negative log likelihoods of alternative models and the baseline model. Bars represent mean ± 95% CI of the bootstrap distribution. Each panel includes a zoomed-in inset to the right. **A.** Reliability. **B.** Volatility. **C.** Controllability.

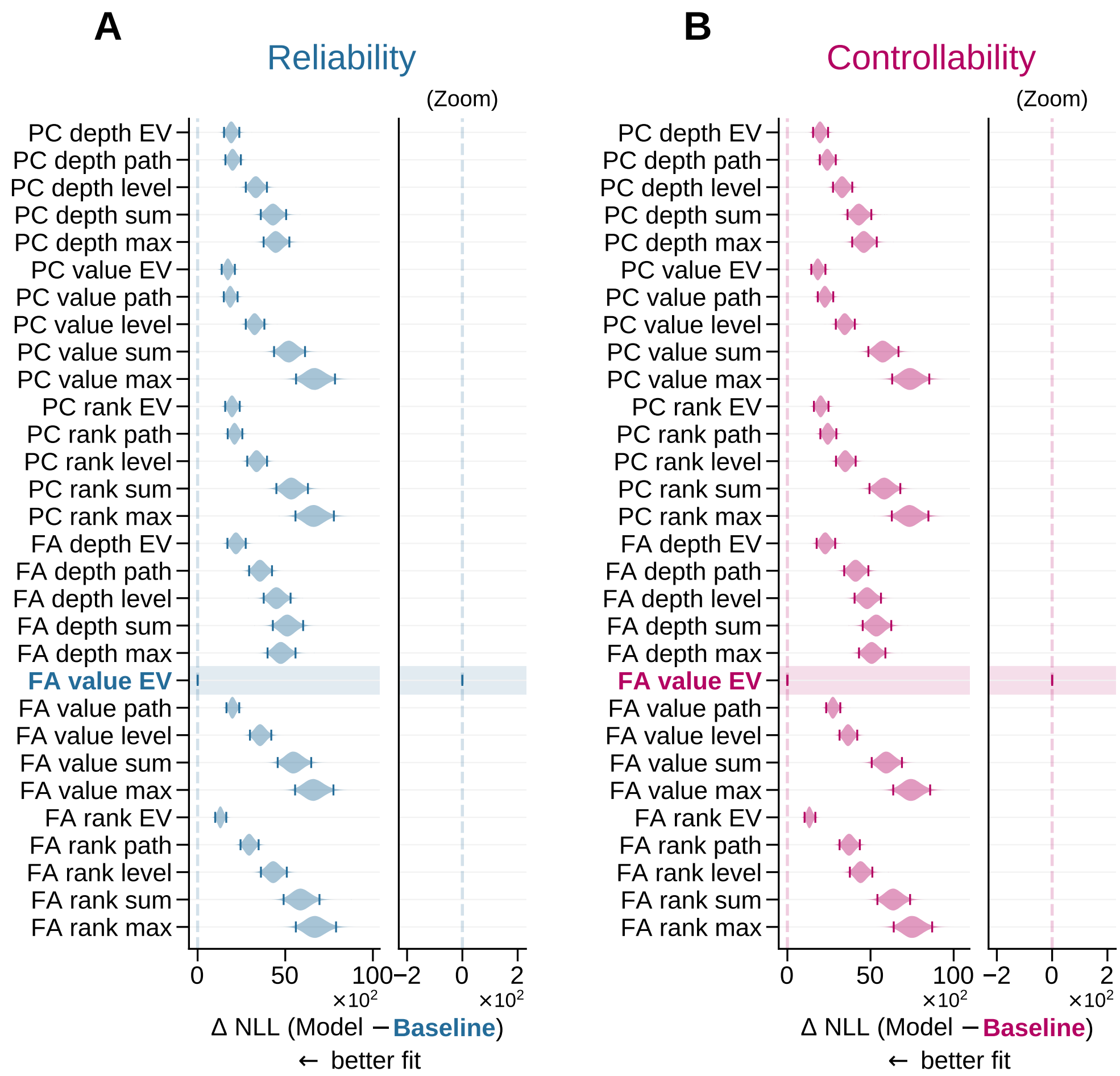

Fig. S28: Model Recovery based on Simulated Data from the FA Value-filtering EV (baseline) model. Bootstrapped differences ($n = 100$ simulated participants per condition, 10^6 resamples) between the 5-fold cross-validated negative log likelihoods of alternative models and the baseline model. Bars represent mean ± 95% CI of the bootstrap distribution. Each panel includes a zoomed-in inset to the right. **A.** Reliability. **B.** Controllability.

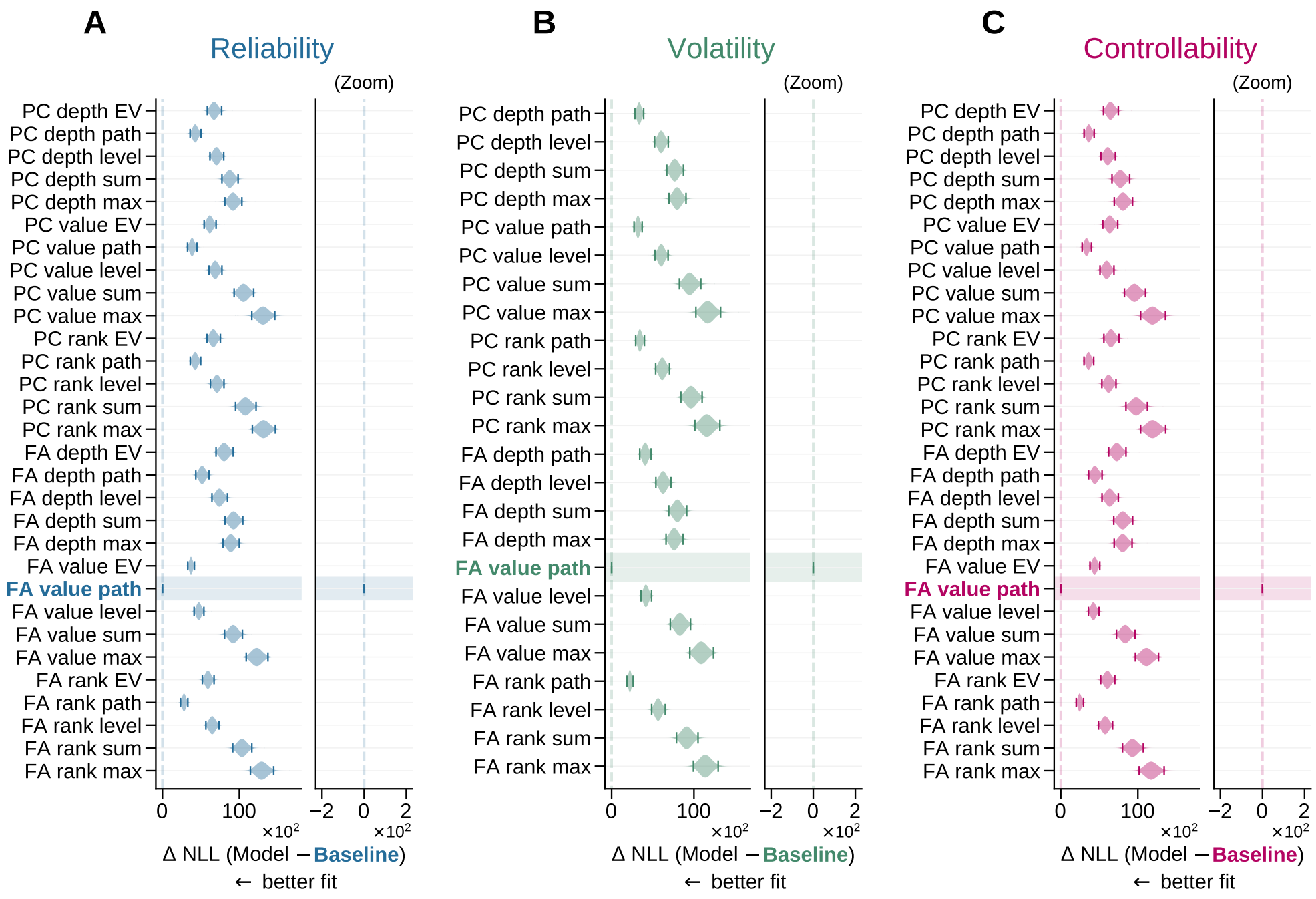

Fig. S29: Model Recovery based on Simulated Data from the FA Value-filtering Path (baseline) model. Bootstrapped differences ($n = 100$ simulated participants per condition, 10^6 resamples) between the 5-fold cross-validated negative log likelihoods of alternative models and the baseline model. Bars represent mean ± 95% CI of the bootstrap distribution. Each panel includes a zoomed-in inset to the right. **A.** Reliability. **B.** Volatility. **C.** Controllability.

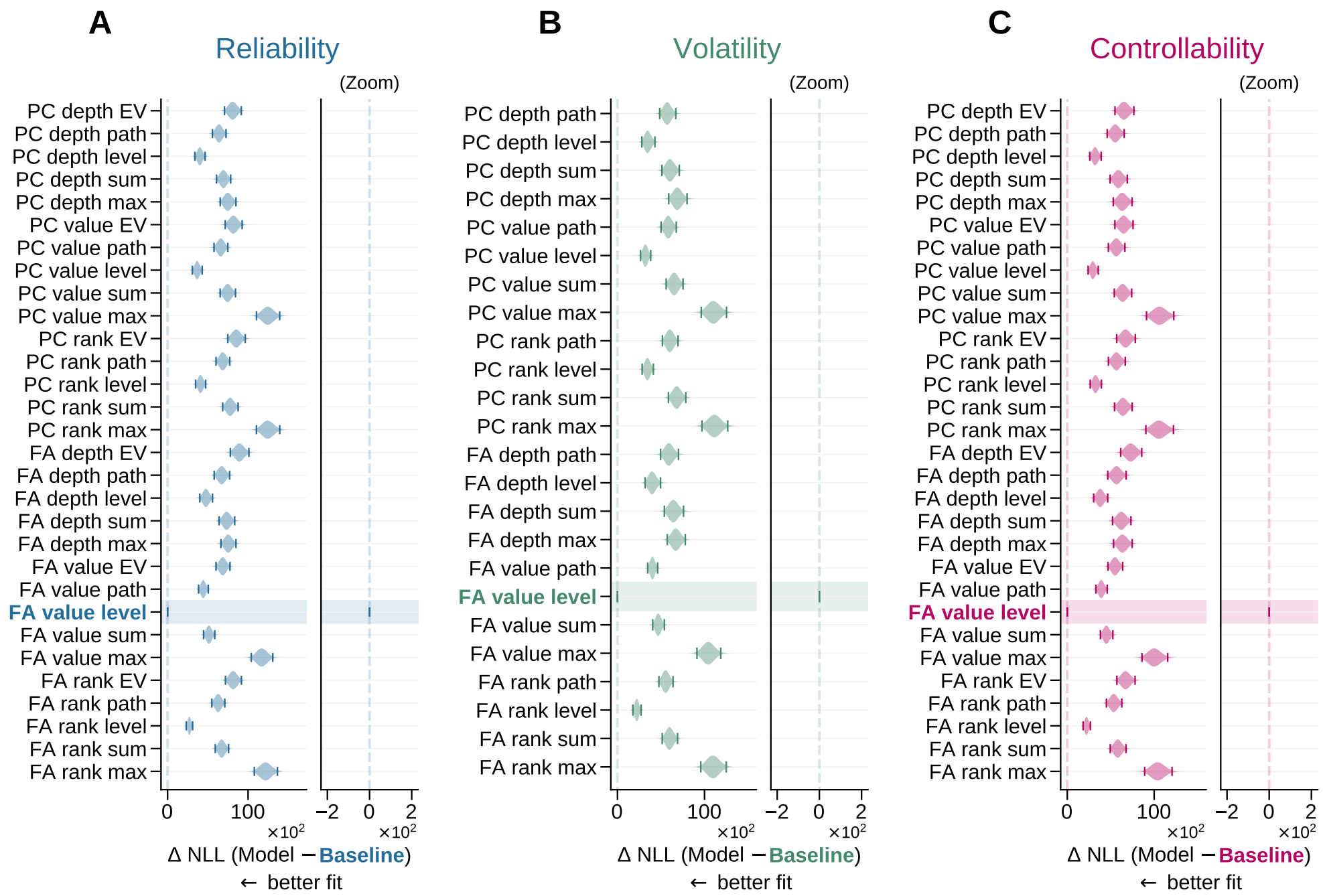

Fig. S30: Model Recovery based on Simulated Data from the FA Value-filtering Level-Mean (baseline) model. Bootstrapped differences ($n = 100$ simulated participants per condition, 10^6 resamples) between the 5-fold cross-validated negative log likelihoods of alternative models and the baseline model. Bars represent mean ± 95% CI of the bootstrap distribution. Each panel includes a zoomed-in inset to the right. **A.** Reliability. **B.** Volatility. **C.** Controllability.

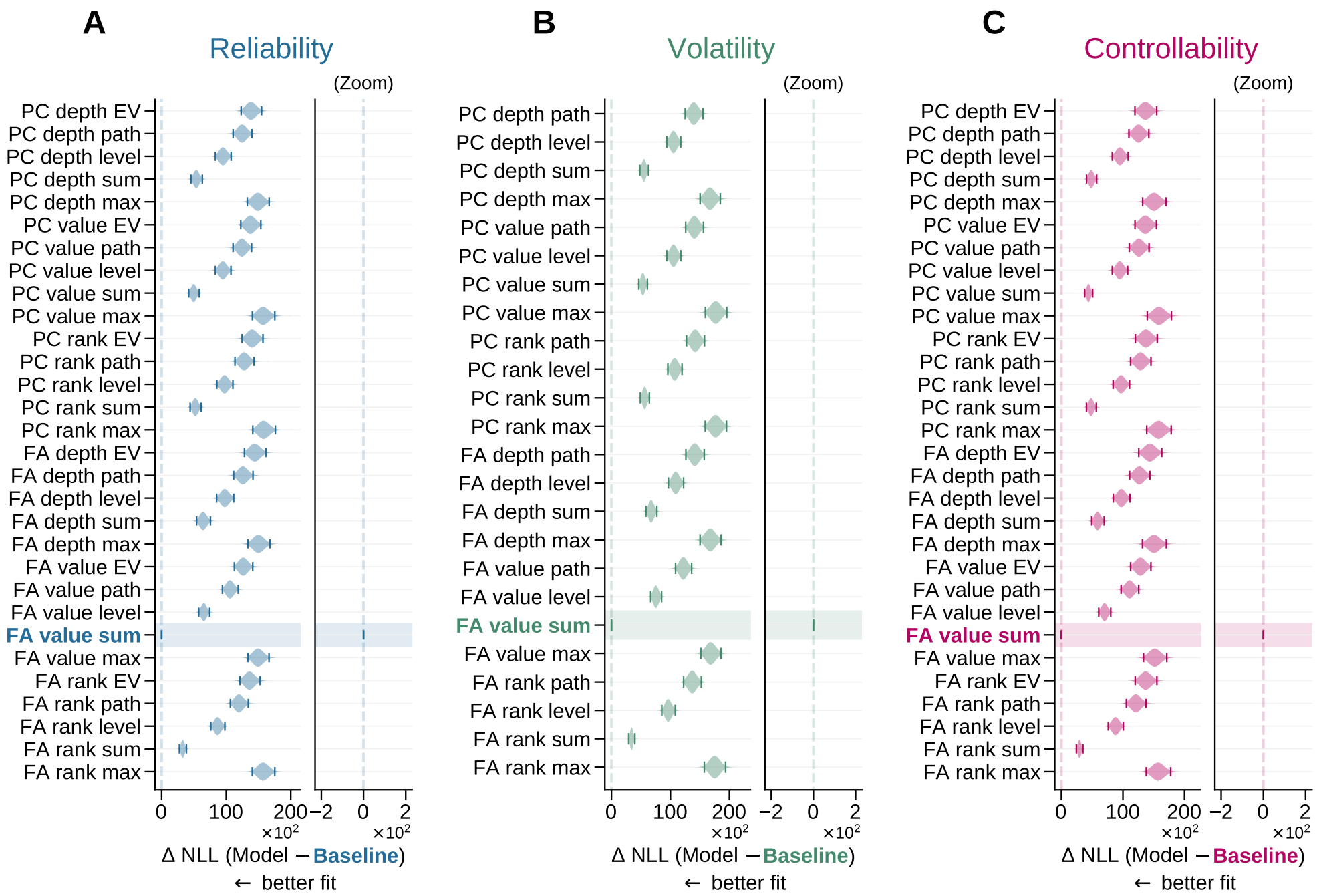

Fig. S31: Model Recovery based on Simulated Data from the FA Value-filtering Sum (baseline) model. Bootstrapped differences ($n = 100$ simulated participants per condition, 10^6 resamples) between the 5-fold cross-validated negative log likelihoods of alternative models and the baseline model. Bars represent mean ± 95% CI of the bootstrap distribution. Each panel includes a zoomed-in inset to the right. **A.** Reliability. **B.** Volatility. **C.** Controllability.

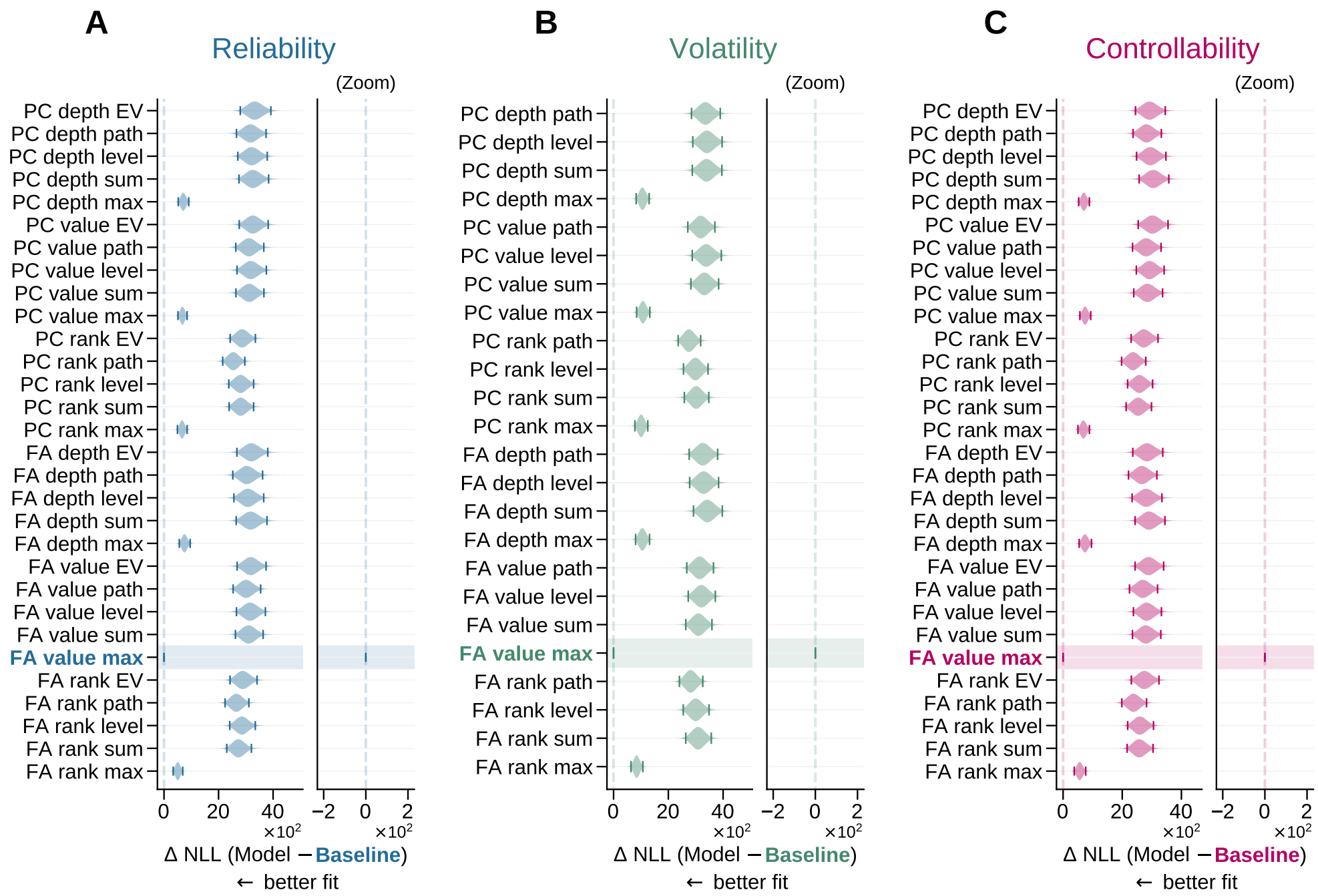

Fig. S32: Model Recovery based on Simulated Data from the FA Value-filtering Max (baseline) model. Bootstrapped differences ($n = 100$ simulated participants per condition, 10^6 resamples) between the 5-fold cross-validated negative log likelihoods of alternative models and the baseline model. Bars represent mean ± 95% CI of the bootstrap distribution. Each panel includes a zoomed-in inset to the right. **A.** Reliability. **B.** Volatility. **C.** Controllability.

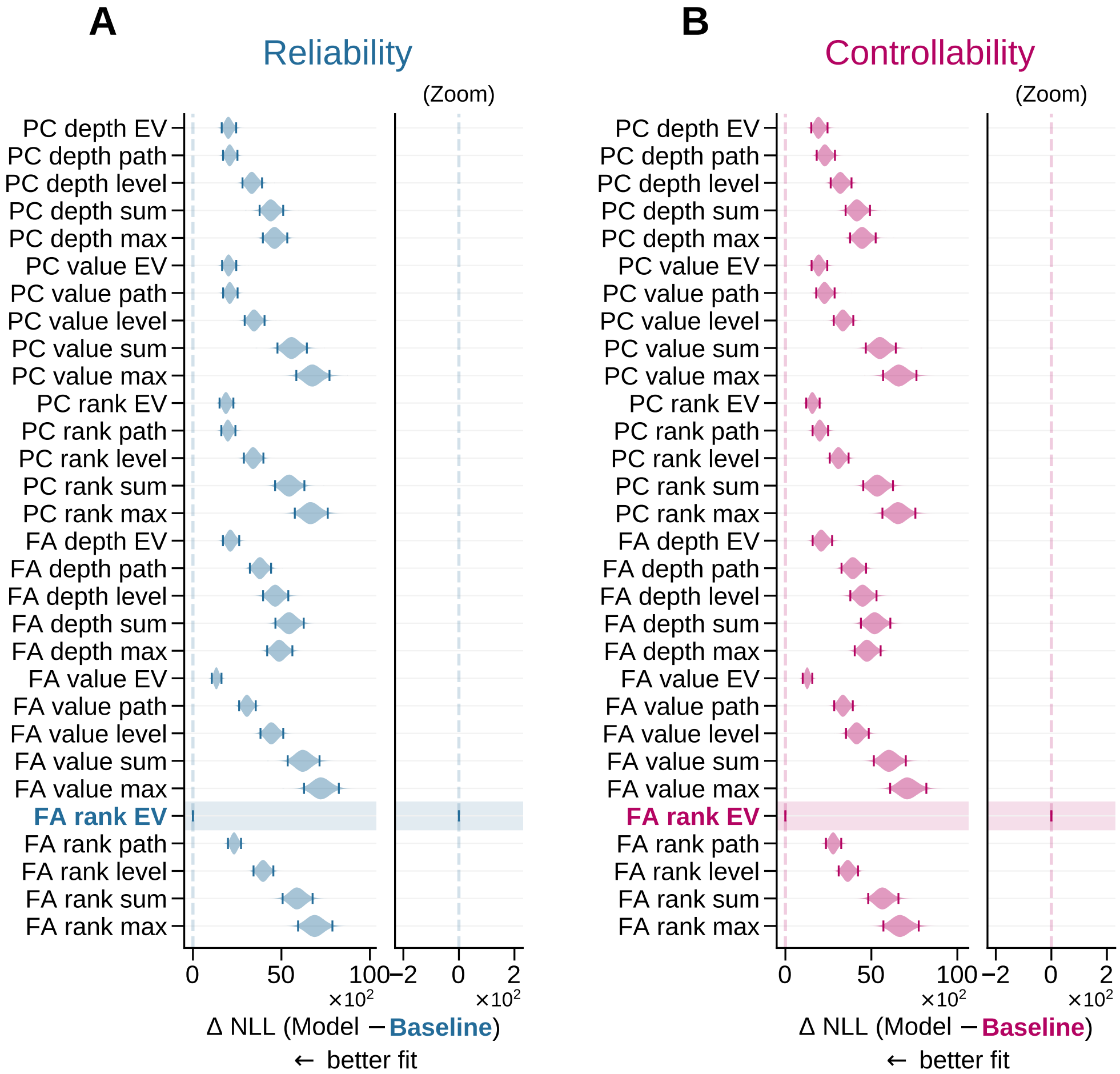

Fig. S33: Model Recovery based on Simulated Data from the FA Rank-filtering EV (baseline) model. Bootstrapped differences (n = 100 simulated participants per condition, 10^6 resamples) between the 5-fold cross-validated negative log likelihoods of alternative models and the baseline model. Bars represent mean ± 95% CI of the bootstrap distribution. Each panel includes a zoomed-in inset to the right. **A.** Reliability. **B.** Controllability.

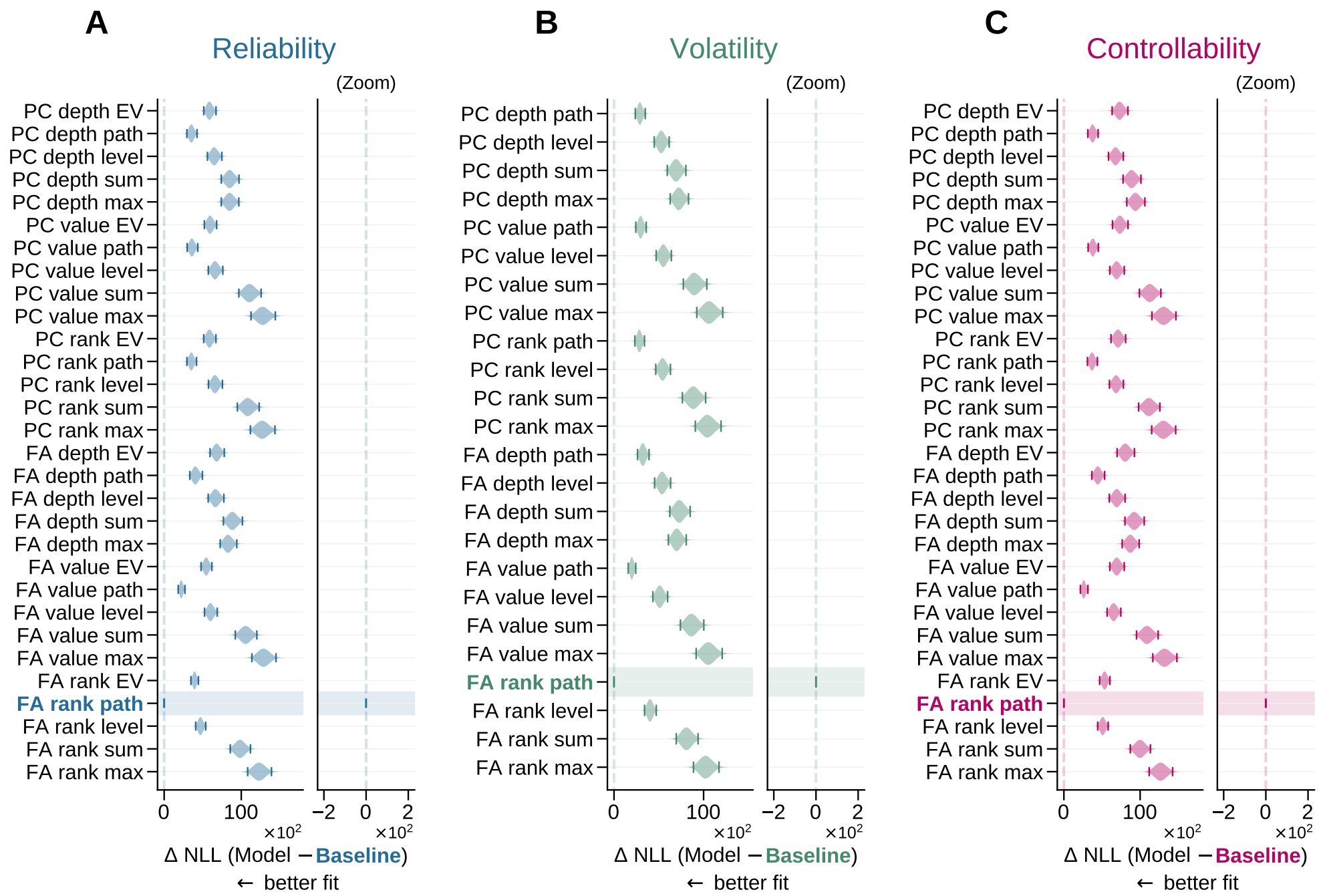

Fig. S34: Model Recovery based on Simulated Data from the FA Rank-filtering Path (baseline) model. Bootstrapped differences (n = 100 simulated participants per condition, 10^6 resamples) between the 5-fold cross-validated negative log likelihoods of alternative models and the baseline model. Bars represent mean ± 95% CI of the bootstrap distribution. Each panel includes a zoomed-in inset to the right. **A.** Reliability. **B.** Volatility. **C.** Controllability.

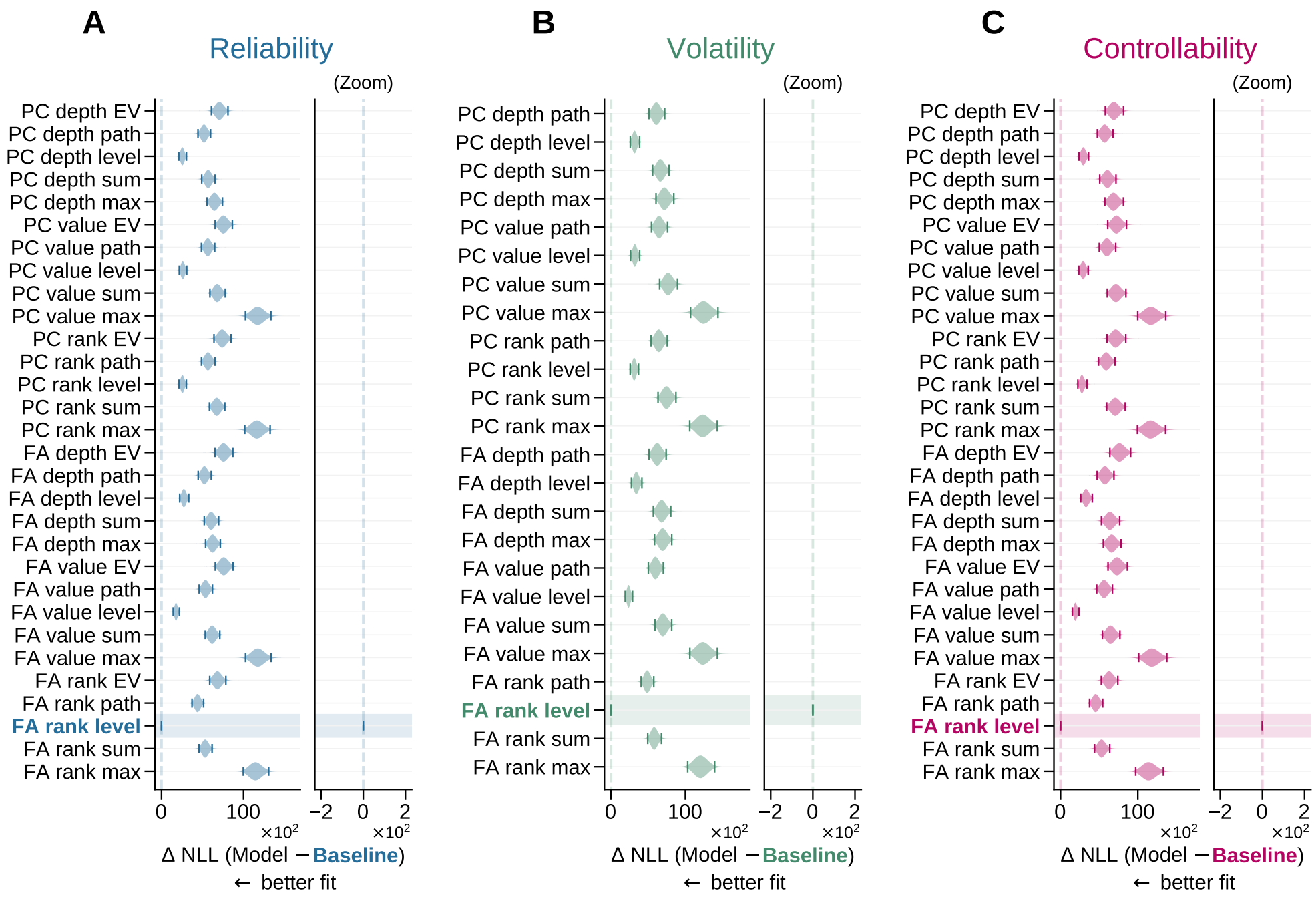

Fig. S35: Model Recovery based on Simulated Data from the FA Rank-filtering Level-Mean (baseline) model. Bootstrapped differences ($n = 100$ simulated participants per condition, 10^6 resamples) between the 5-fold cross-validated negative log likelihoods of alternative models and the baseline model. Bars represent mean ± 95% CI of the bootstrap distribution. Each panel includes a zoomed-in inset to the right. **A.** Reliability. **B.** Volatility. **C.** Controllability.

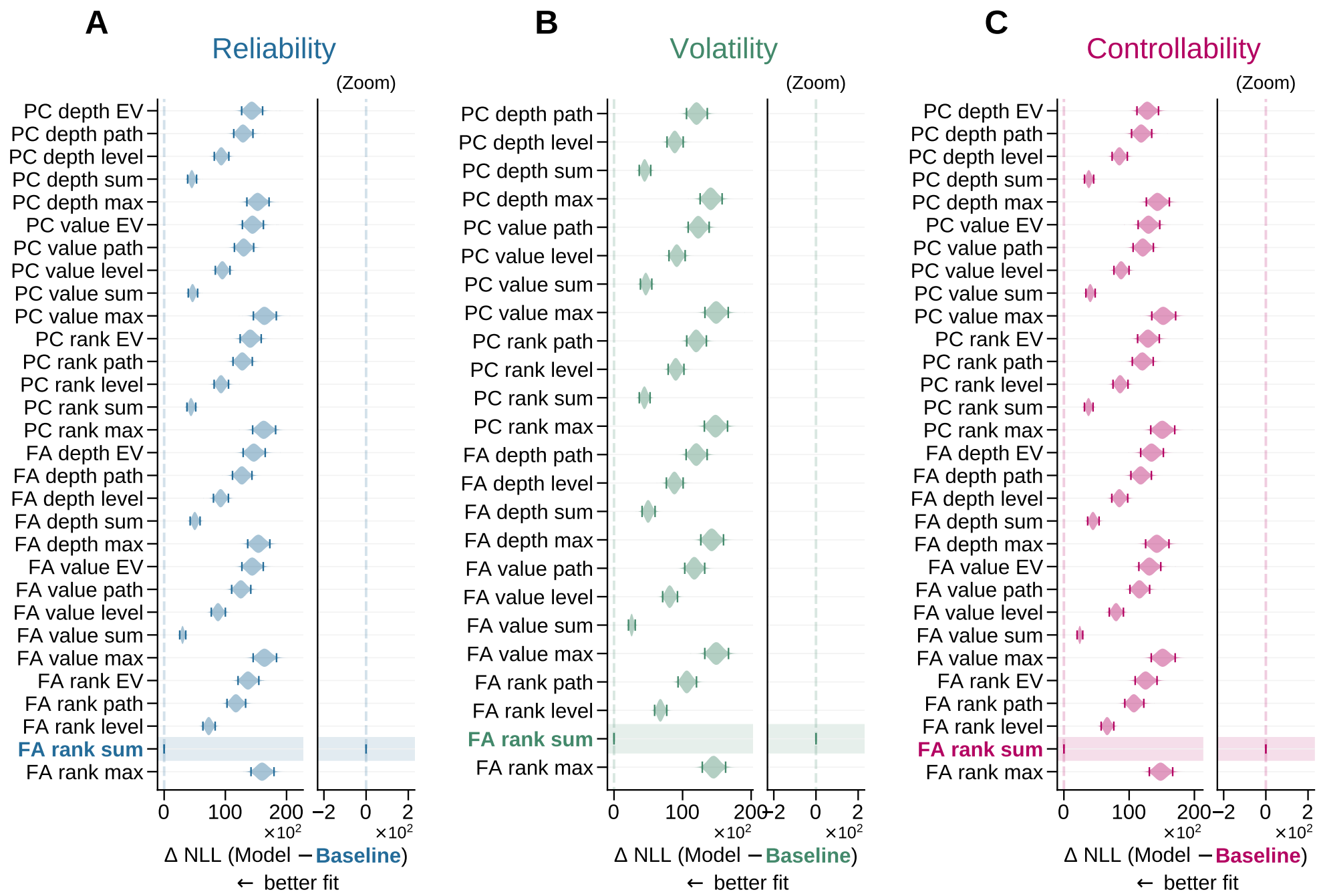

Fig. S36: Model Recovery based on Simulated Data from the FA Rank-filtering Sum (baseline) model. Bootstrapped differences (n = 100 simulated participants per condition, 10^6 resamples) between the 5-fold cross-validated negative log likelihoods of alternative models and the baseline model. Bars represent mean ± 95% CI of the bootstrap distribution. Each panel includes a zoomed-in inset to the right. **A.** Reliability. **B.** Volatility. **C.** Controllability.

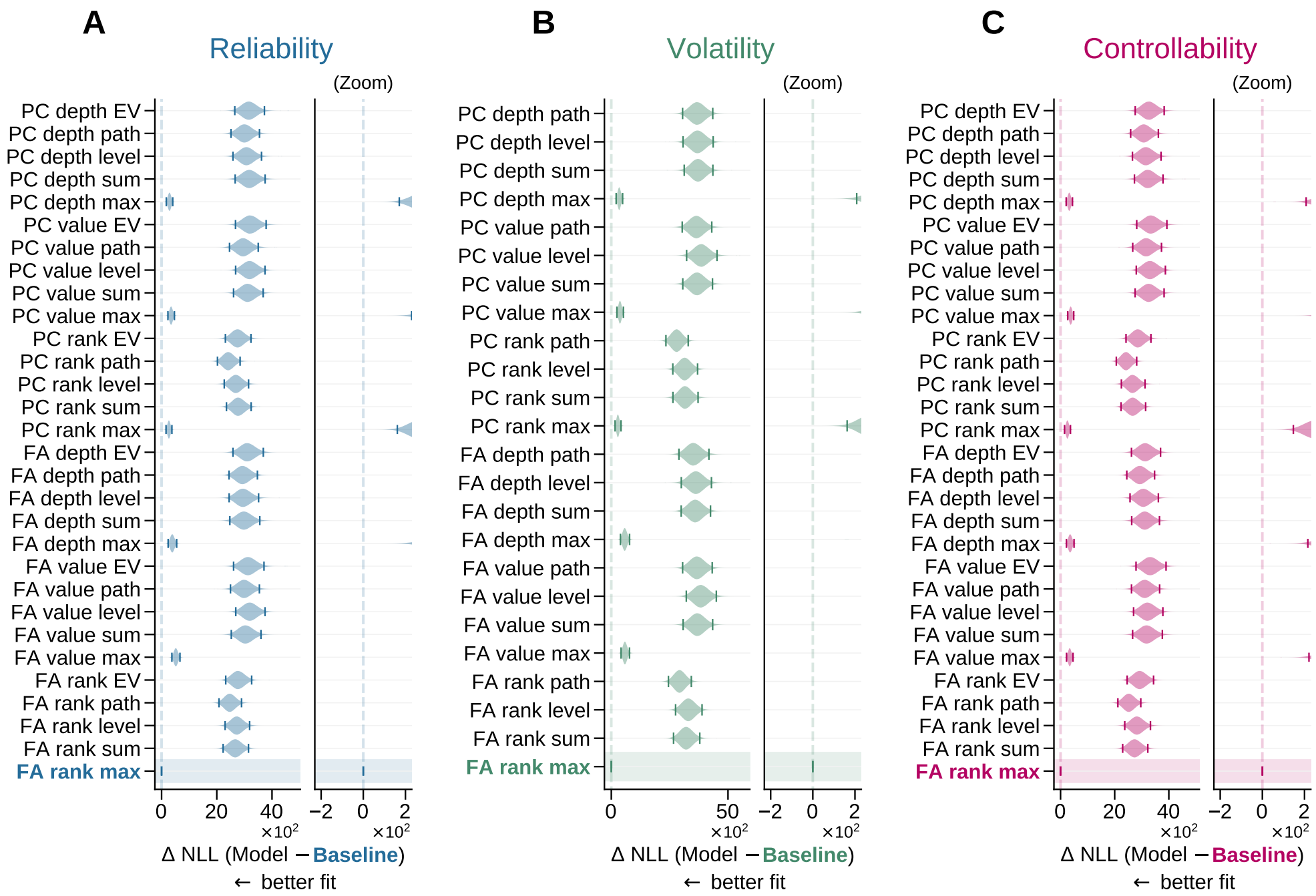

Fig. S37: Model Recovery based on Simulated Data from the FA Rank-filtering Max (baseline) model. Bootstrapped differences ($n = 100$ simulated participants per condition, 10^6 resamples) between the 5-fold cross-validated negative log likelihoods of alternative models and the baseline model. Bars represent mean ± 95% CI of the bootstrap distribution. Each panel includes a zoomed-in inset to the right. **A.** Reliability. **B.** Volatility. **C.** Controllability.

7.3 Parameter recovery

We have now shown that our models are recoverable, but we also would like to show that each of the parameters are individually identifiable. To do this, we took the fitted synthetic data and compared the fitted parameters with the ground-truth parameter values.

Successful recovery of the parameters means that the majority of the points should lie on the diagonal; we find that this is indeed the case for our best-fitting models, the PC Depth-filtering Path (Fig S38) and Level-Mean (Fig S39), demonstrating that our parameter recovery is sound. We also find that the PC Depth-filtering Sum (Fig S40) and Max (Fig S41) models, which are also competitive in the Volatility condition, show decent recovery. As expected, parameter recoverability naturally decreases in regions of structural non-identifiability — e.g., at high depth levels where deeper planners behave identically once the horizon is reached, and for extreme $\log(\beta)$ values where behavior becomes either near-random or near-deterministic. These regimes correspond to regions where the likelihood surface is flat by construction.

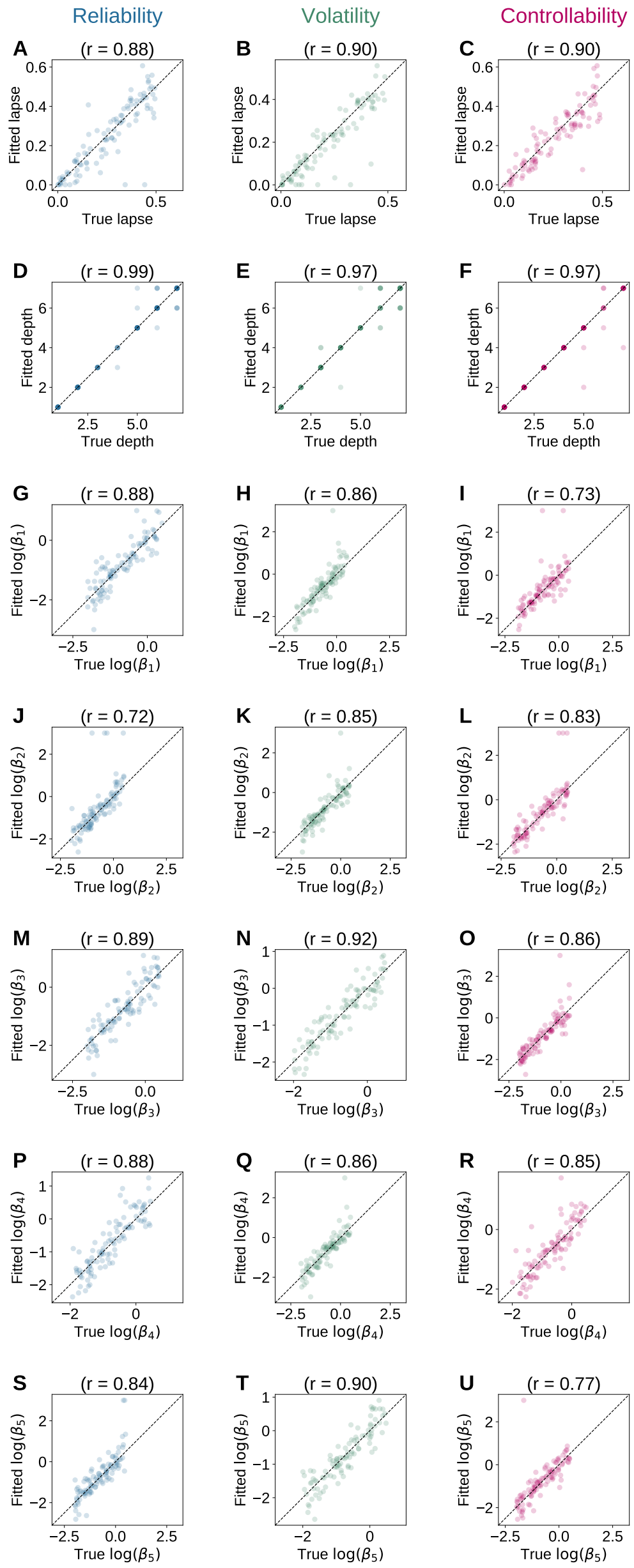

Fig. S38: Parameter Recovery for Simulated Data from the PC Depth-filtering Path model (n = 100 simulated participants per experiment). **A–C.** Ground truth depth versus fitted depth. **D–F.** Ground truth lapse vs fitted lapse. **G–U.** Ground truth log(β) versus fitted log(β), for each stochasticity level of the given condition.

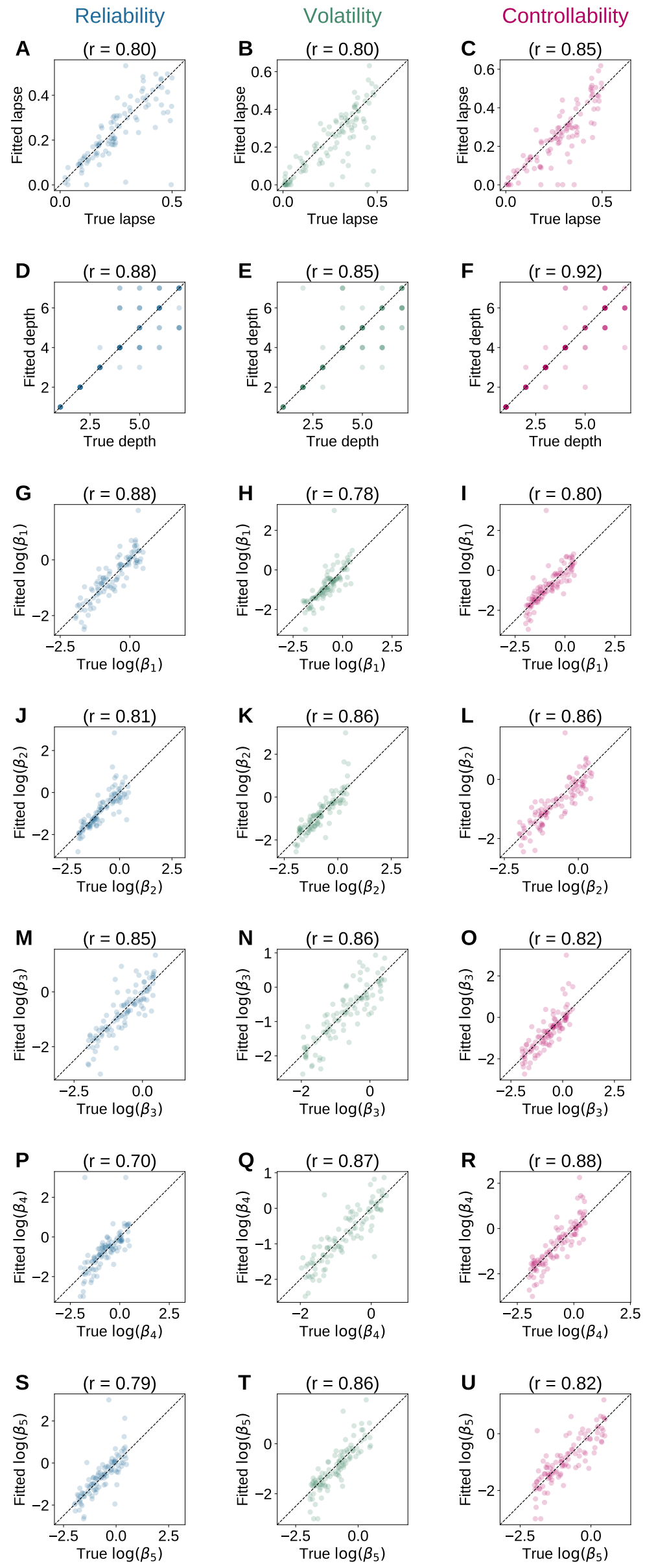

Fig. S39: Parameter Recovery for Simulated Data from the PC Depth-filtering Level-Mean model (n = 100 simulated participants per experiment). **A–C.** Ground truth depth versus fitted depth. **D–F.** Ground truth lapse vs fitted lapse. **G–U.** Ground truth log(β) versus fitted log(β), for each stochasticity level of the given condition.

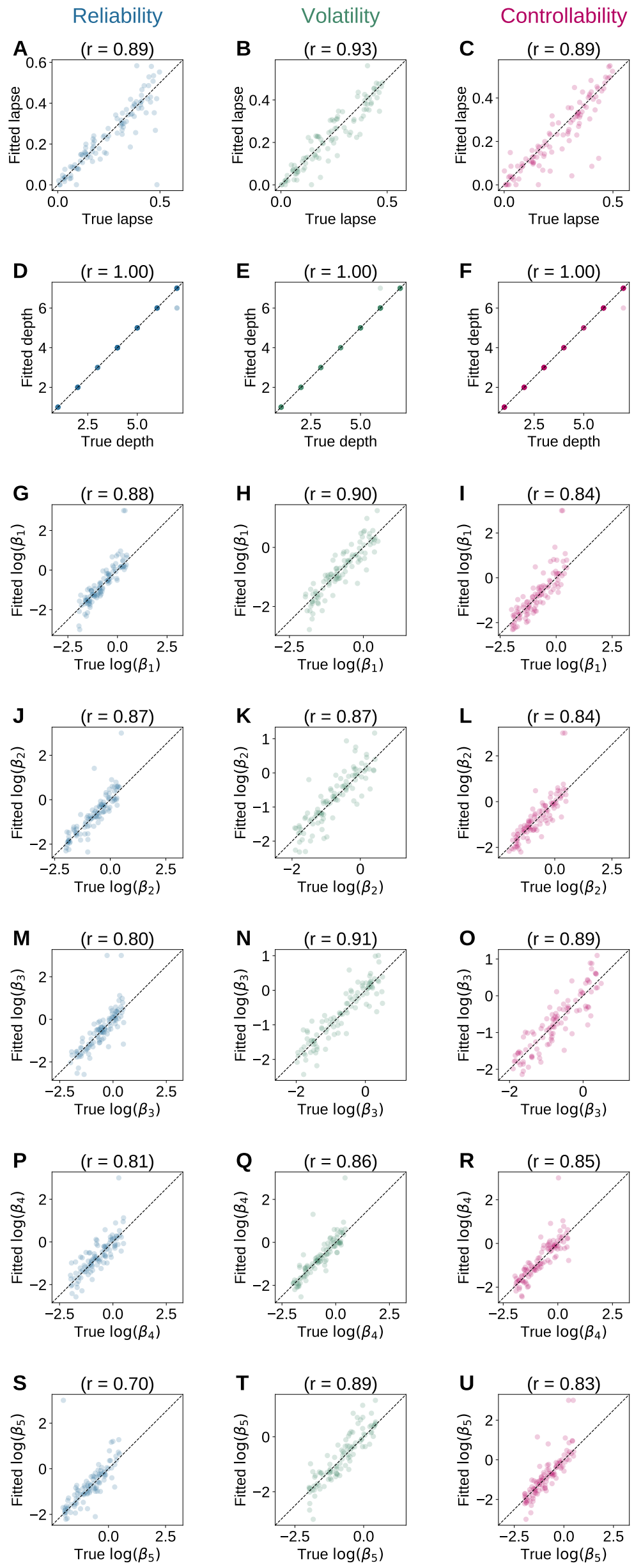

Fig. S40: Parameter Recovery for Simulated Data from the PC Depth-filtering Sum model (n = 100 simulated participants per experiment). **A–C.** Ground truth depth versus fitted depth. **D–F.** Ground truth lapse vs fitted lapse. **G–U.** Ground truth $\log(\beta)$ versus fitted $\log(\beta)$, for each stochasticity level of the given condition.

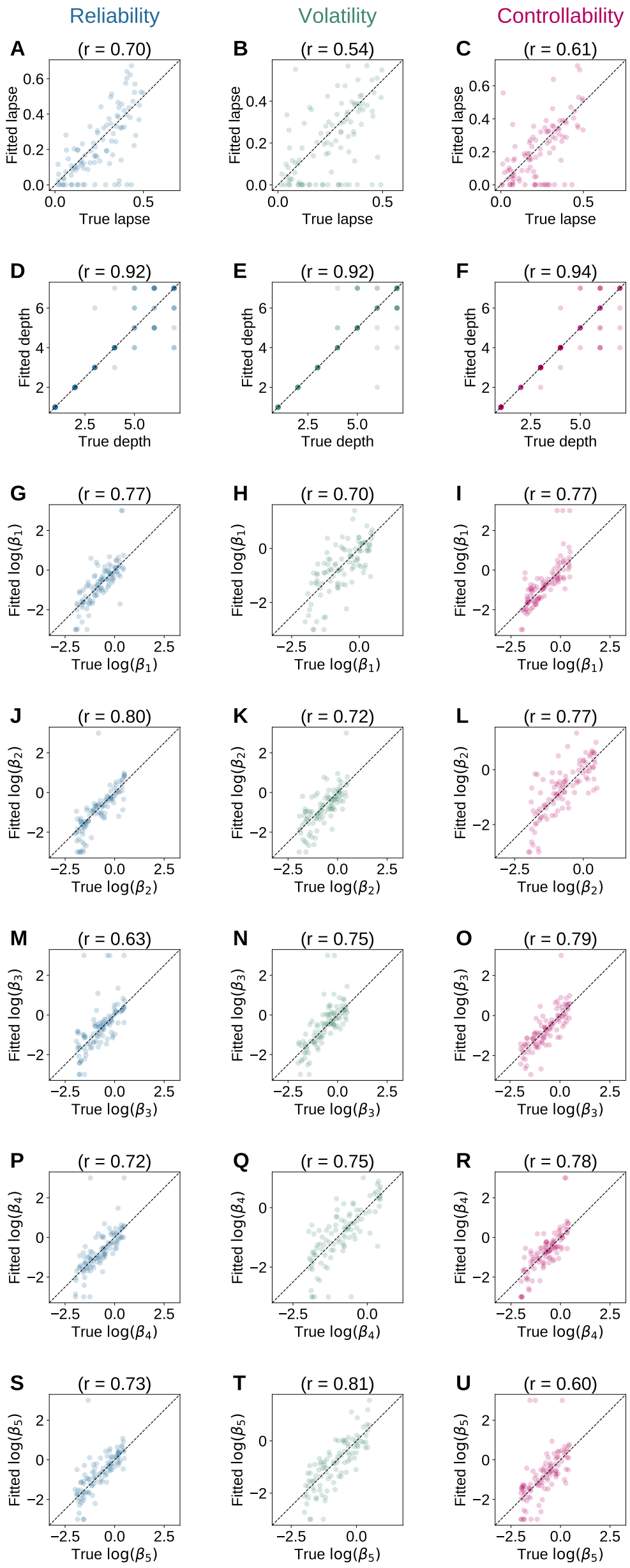

Fig. S41: Parameter Recovery for Simulated Data from the PC Depth-filtering Max model (n = 100 simulated participants per experiment). **A–C.** Ground truth depth versus fitted depth. **D–F.** Ground truth lapse vs fitted lapse. **G–U.** Ground truth $\log(\beta)$ versus fitted $\log(\beta)$, for each stochasticity level of the given condition.

Supplementary Note 8: Model heterogeneity

To assess potential heterogeneity in planning strategies across participants, we conducted random-effects Bayesian model comparison (RFX-BMC) across participants. This analysis estimates the probability that each model is the most frequent in the population, explicitly allowing for heterogeneity in strategy use across individuals [3].

Consistent with our model comparison procedure in the main text, we used cross-validated negative log-likelihood (CV-NLL), aggregated across folds (and across repeated random CV seeds where applicable), as a sample-based approximation to log model evidence for each participant and model. These subject-level estimates were then entered into a RFX-BMC procedure implemented in Python (adapted from the VBA toolbox [3]). This procedure yields two key summary statistics: (1) *posterior model frequency*, the estimated proportion of participants best explained by each model, and (2) the *protected exceedance probability* (PXP), the posterior probability that a given model is more frequent than all others in the population, accounting for the possibility that observed differences arise by chance.

8.1 Robustness of heterogeneity analysis

We treat RFX-BMC as a complementary summary of model heterogeneity rather than the primary basis for our main conclusions, which are based on aggregate cross-validated model performance. In particular, we are cautious about over-interpreting the RFX-BMC results because several closely related model classes exhibited similar cross-validated predictive performance. In this regime, RFX-BMC can be sensitive to small variations in subject-level evidence arising from different cross-validation partitions, which can lead to variability in posterior model frequencies and protected exceedance probabilities.

To assess the robustness of the RFX-BMC procedure on our data, we re-split the empirical data with five independent CV seeds, re-fit all models under each split, and applied RFX-BMC separately to each fit cohort—five instances of 5-fold cross-validation in total. This analysis quantifies the stability of RFX-BMC inferences under reasonable perturbations of the data partitioning and allows us to assess whether observed patterns of model heterogeneity are robust to the choice of CV split. Importantly, these repeated analyses were used exclusively as a robustness check for model heterogeneity; the additional CV realizations were not used in the primary aggregate model-comparison figures or to select among models.

8.2 Heterogeneity results

As anticipated, the RFX-BMC procedure exhibited substantial variability across cross-validation realizations, indicating sensitivity to small differences in subject-level model evidence induced by the CV partitioning procedure (Fig S42 - S46). Notably, we did not find similar instability in our main model comparison result. Despite the variability in RFX-BMC inferences, a consistent qualitative pattern emerged across all stochasticity conditions: models belonging to the PC Depth-filtering family reliably achieved the highest posterior model frequencies and protected exceedance probabilities, supporting the broader conclusion that this model class best captures participant

behavior. By contrast, finer-grained distinctions between closely related value-function variants within the PC Depth-filtering family (e.g., Level-Mean, Sum, Max, Path) were considerably less stable across CV realizations. In many cases, differences in posterior model frequency between these variants were small, and protected exceedance probabilities remained modest, indicating insufficient evidence to support strong claims about participant-level preference for specific variants. Importantly, non-determinizing expected-value models rarely achieved the largest exceedance probabilities (see Fig S43F for the singular instance where this was the case), further supporting the broader conclusion that participants relied primarily on determinizing planning strategies. Together, these analyses suggest that the aggregate computational principles identified in the main text are robust, while finer-grained claims about heterogeneous strategy selection remain underdetermined by the present dataset.

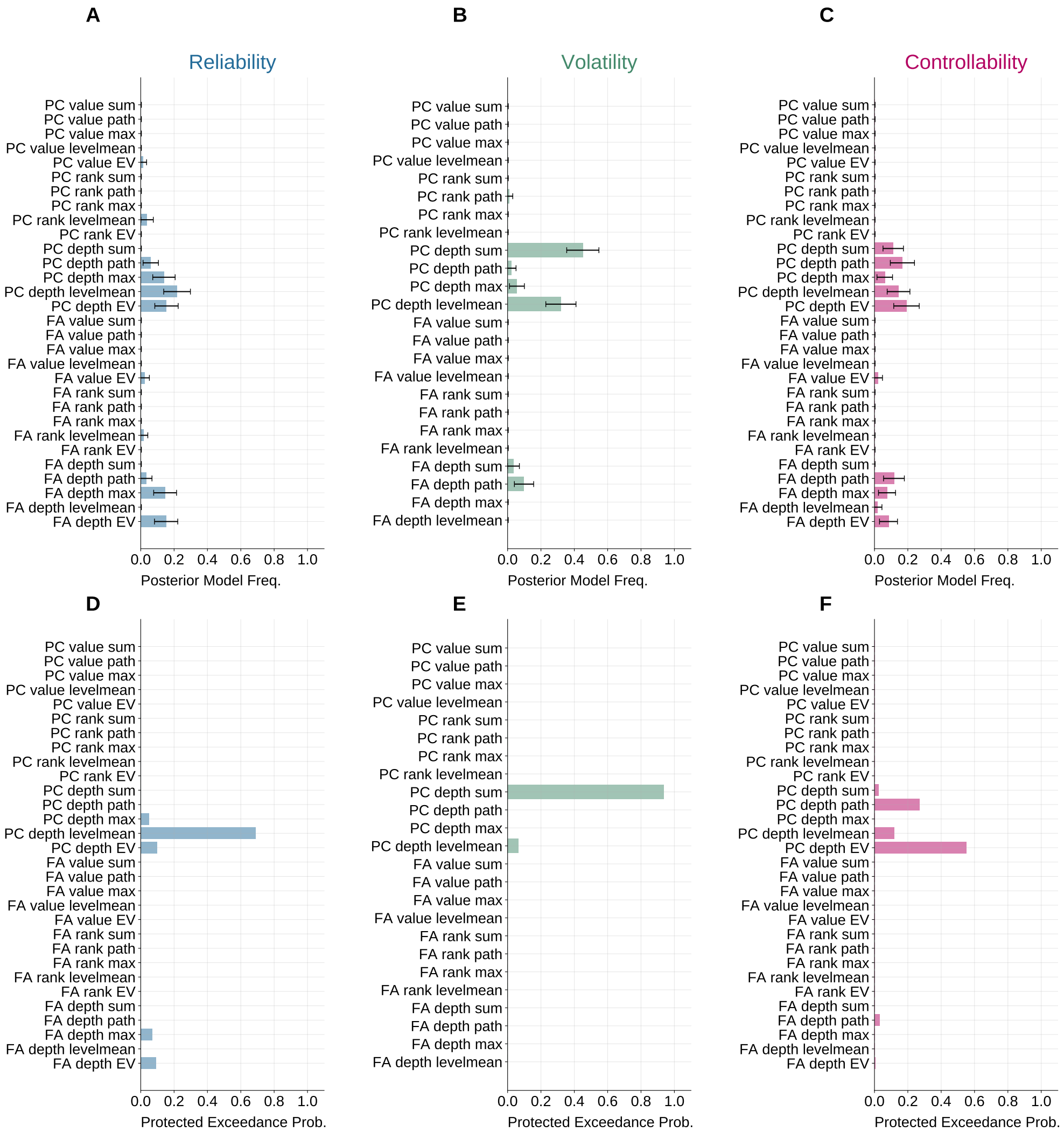

Fig. S42: Random-Effects Bayesian Model Comparison (n = 100 participants per condition). **A–C.** Estimated posterior model frequencies. Bars show the posterior mean and error bars the 95% credible interval (±1.96 posterior SD). **D–F.** Protected exceedance probabilities for each model. Both quantities are parameters of a single group-level posterior estimated from all participants jointly, not averages over per-participant values; protected exceedance probabilities are point-valued and so carry no interval.

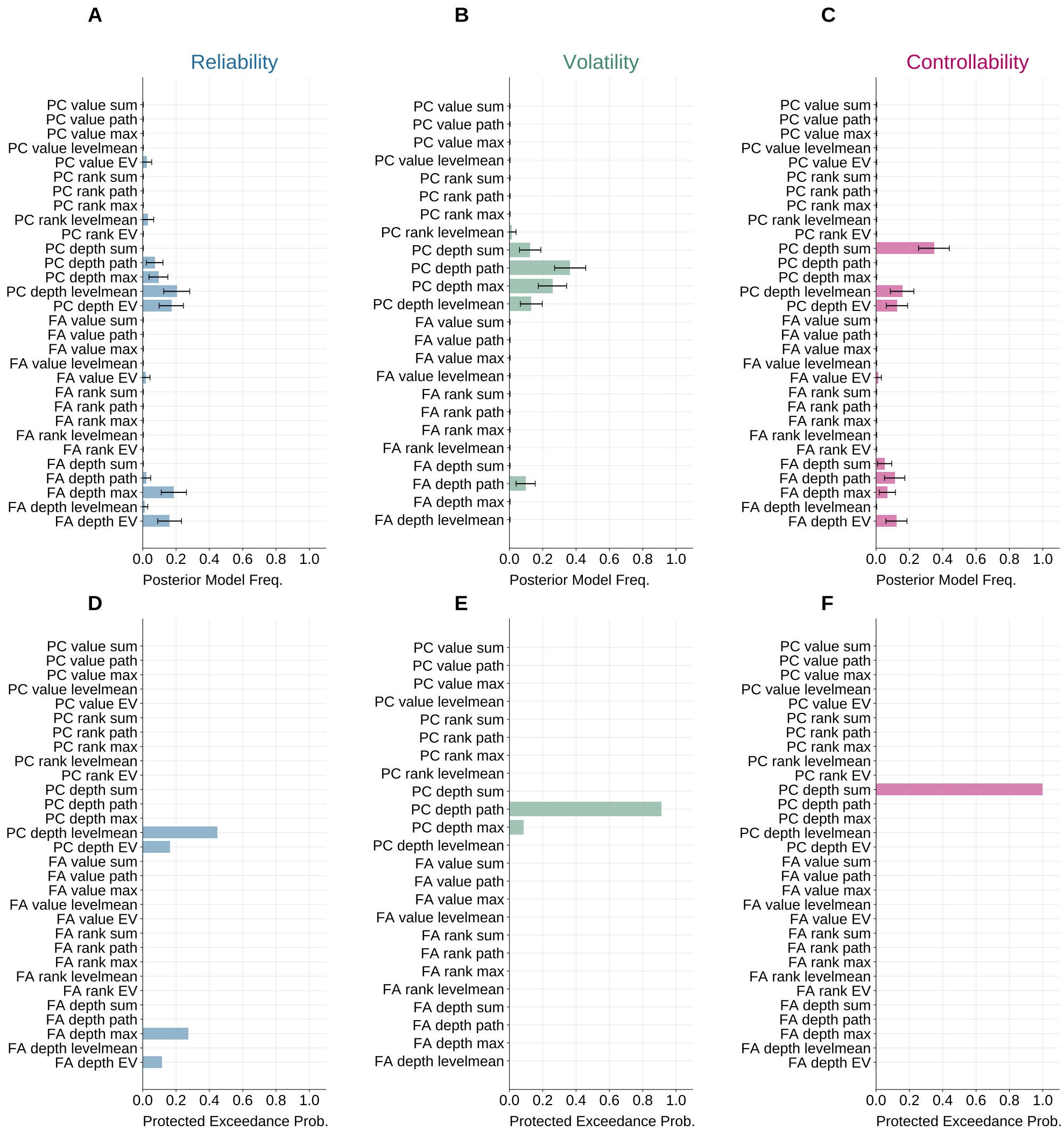

Fig. S43: Random-Effects Bayesian Model Comparison Robustness Check 1 ($n = 100$ participants per condition). **A–C.** Estimated posterior model frequencies. Bars show the posterior mean and error bars the 95% credible interval (±1.96 posterior SD). **D–F.** Protected exceedance probabilities for each model. Both quantities are parameters of a single group-level posterior estimated from all participants jointly, not averages over per-participant values; protected exceedance probabilities are point-valued and so carry no interval.

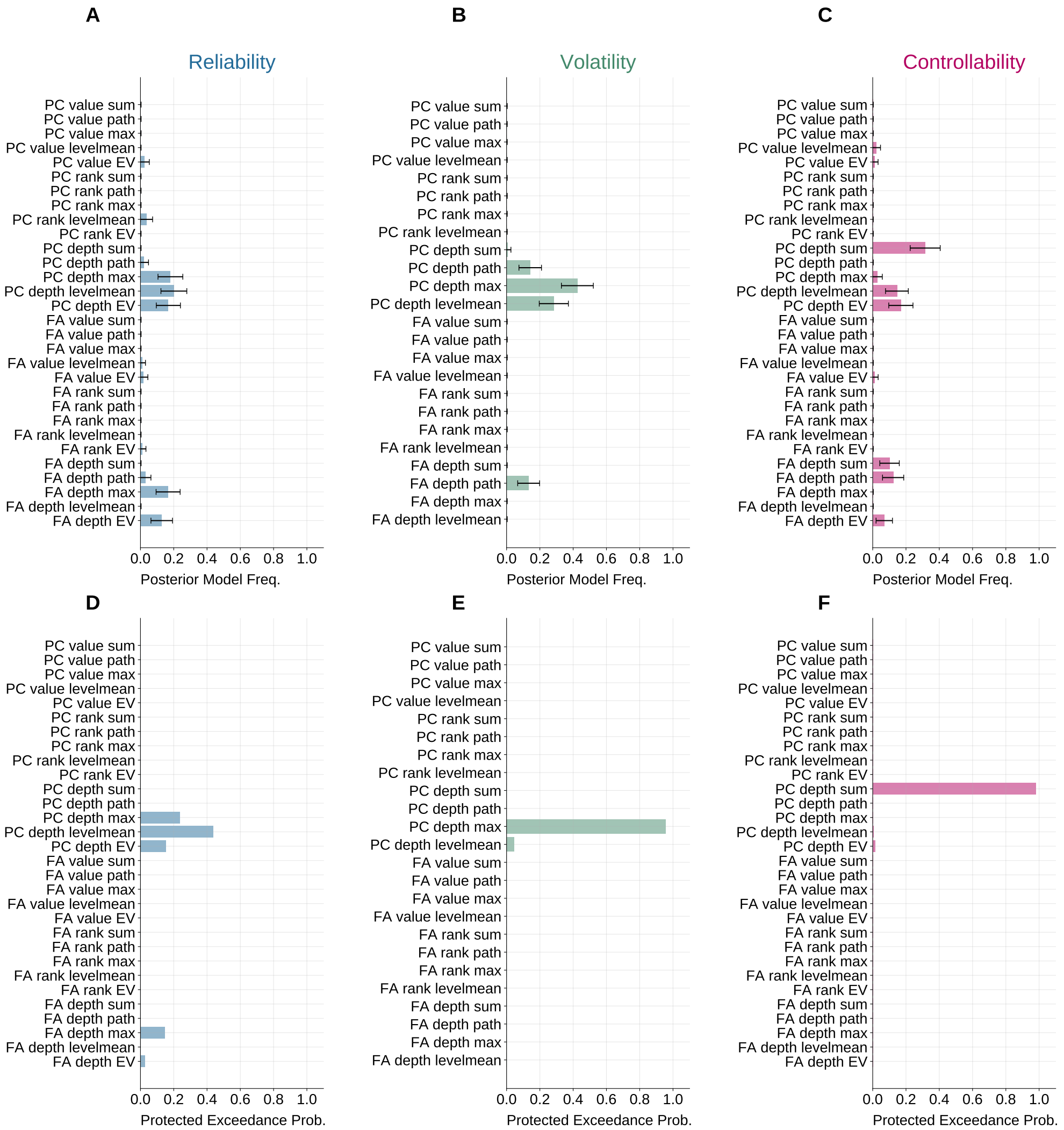

Fig. S44: Random-Effects Bayesian Model Comparison Robustness Check 2 ($n = 100$ participants per condition). **A–C.** Estimated posterior model frequencies. Bars show the posterior mean and error bars the 95% credible interval (±1.96 posterior SD). **D–F.** Protected exceedance probabilities for each model. Both quantities are parameters of a single group-level posterior estimated from all participants jointly, not averages over per-participant values; protected exceedance probabilities are point-valued and so carry no interval.

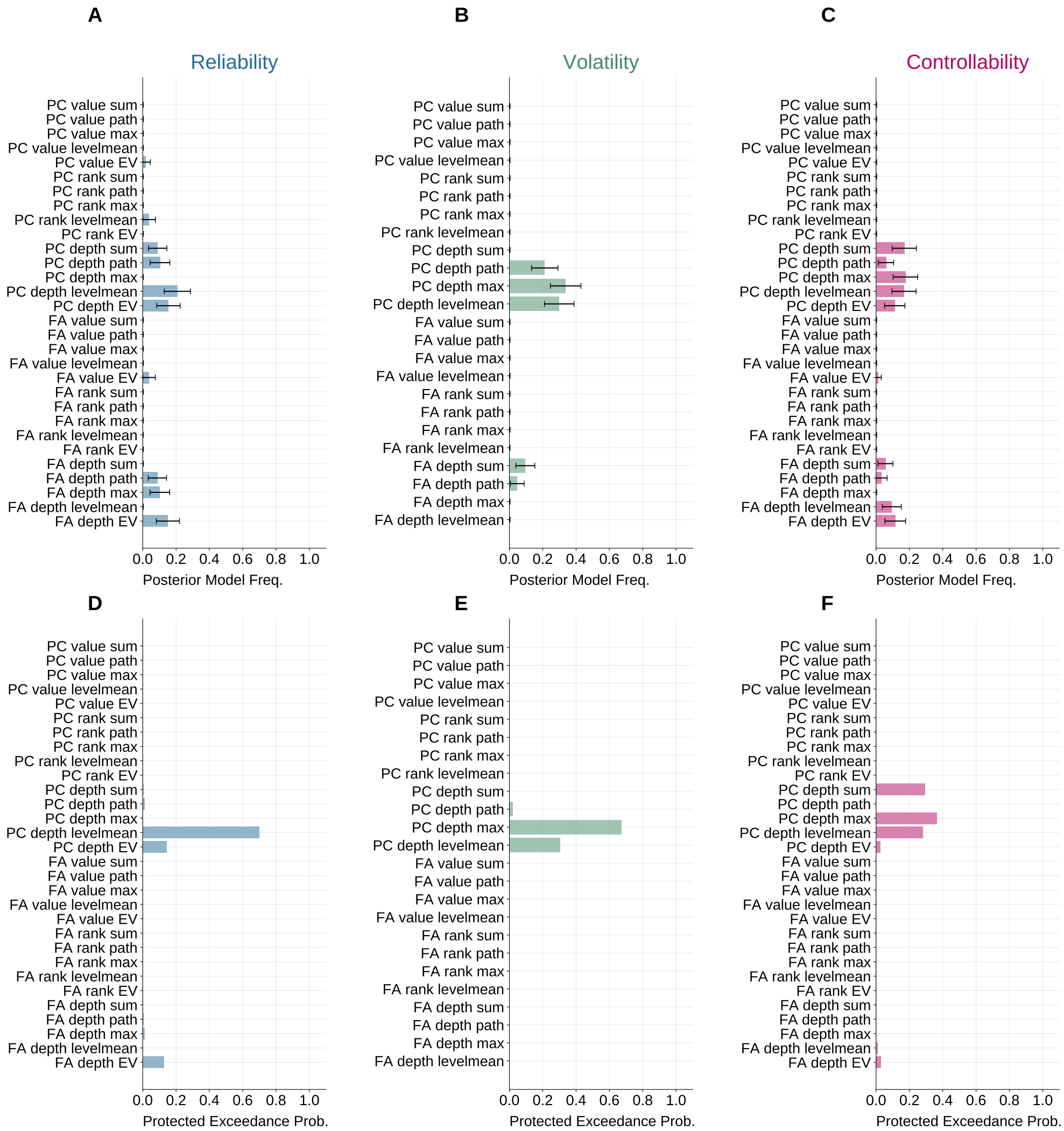

Fig. S45: Random-Effects Bayesian Model Comparison Robustness Check 3 ($n = 100$ participants per condition). **A–C.** Estimated posterior model frequencies. Bars show the posterior mean and error bars the 95% credible interval (±1.96 posterior SD). **D–F.** Protected exceedance probabilities for each model. Both quantities are parameters of a single group-level posterior estimated from all participants jointly, not averages over per-participant values; protected exceedance probabilities are point-valued and so carry no interval.

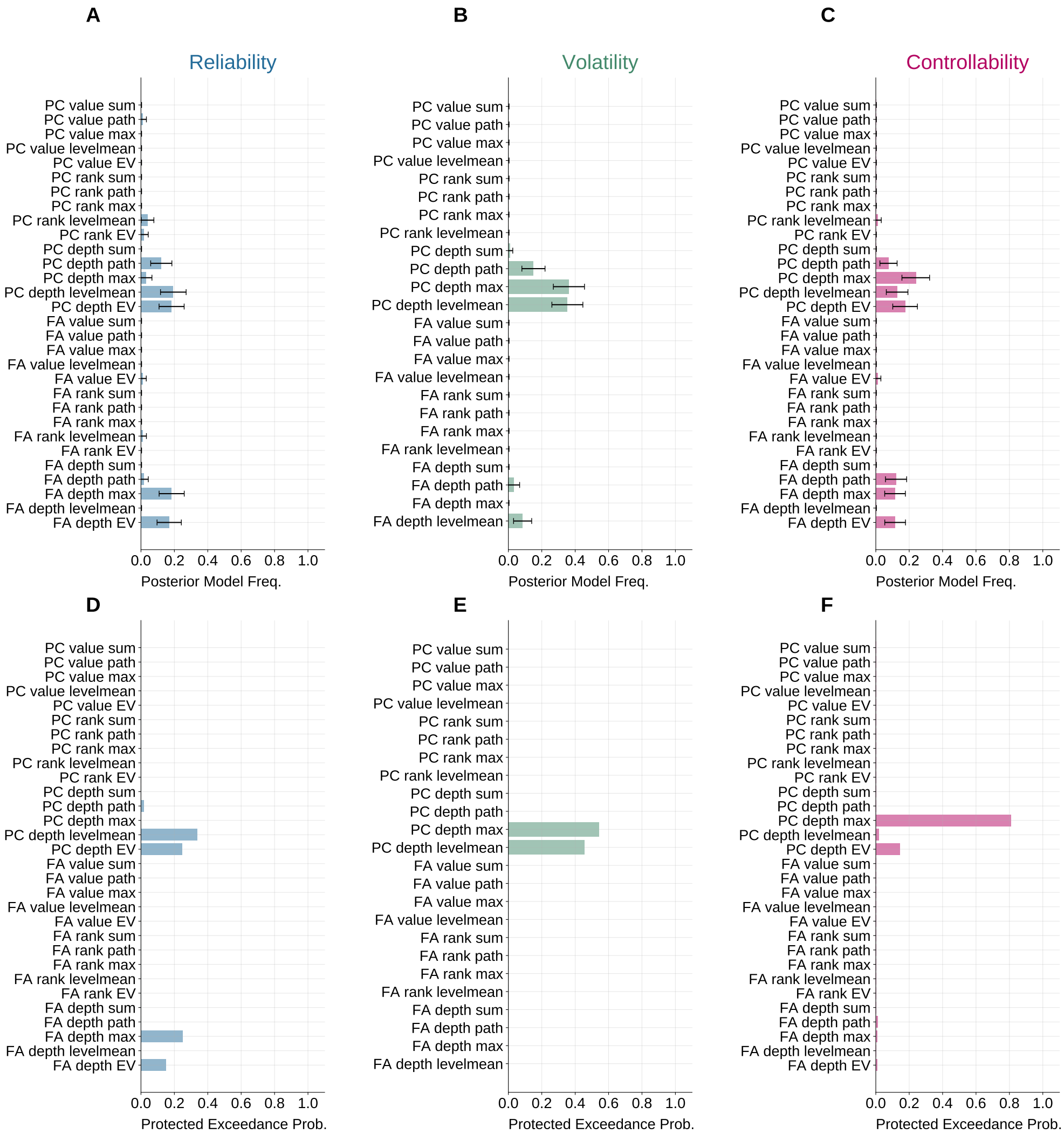

Fig. S46: Random-Effects Bayesian Model Comparison Robustness Check 4 ($n = 100$ participants per condition). **A–C.** Estimated posterior model frequencies. Bars show the posterior mean and error bars the 95% credible interval (± 1.96 posterior SD). **D–F.** Protected exceedance probabilities for each model. Both quantities are parameters of a single group-level posterior estimated from all participants jointly, not averages over per-participant values; protected exceedance probabilities are point-valued and so carry no interval.

Supplementary References

[1] Lieder, F. & Griffiths, T. L. Resource-rational analysis: Understanding human cognition as the optimal use of limited computational resources. *Behavioral and Brain Sciences* **43**, e1 (2020). URL https://www.cambridge.org/core/product/identifier/S0140525X1900061X/type/journal_article.

[2] Callaway, F. *et al. A resource-rational analysis of human planning. Proceedings of the 40th Annual Meeting of the Cognitive Science Society, CogSci 2018*, 178–183 (The Cognitive Science Society, 2018). URL https://collaborate.princeton.edu/en/publications/a-resource-rational-analysis-of-human-planning.

[3] Rigoux, L., Stephan, K. E., Friston, K. J. & Daunizeau, J. Bayesian model selection for group studies - revisited. *NeuroImage* **84**, 971–985 (2014).
