# Supplementary material for: Environmental stochasticity reduces human planning effort
Source: Nat Commun. 2026 Sep 28;17:11199. doi: 10.1038/s41467-026-78023-9 (PMC13714268; doi:10.1038/s41467-026-78023-9)
Supplement: Supplementary file 2 [file 41467_2026_78023_MOESM2_ESM.pdf]

nature portfolio

Corresponding author(s): Jordan Lei

Last updated by author(s): Aug 21, 2026

Reporting Summary

Nature Portfolio wishes to improve the reproducibility of the work that we publish. This form provides structure for consistency and transparency in reporting. For further information on Nature Portfolio policies, see our Editorial Policies and the Editorial Policy Checklist.

Statistics

For all statistical analyses, confirm that the following items are present in the figure legend, table legend, main text, or Methods section.

n/a	Confirmed	
☐	☒	The exact sample size (*n*) for each experimental group/condition, given as a discrete number and unit of measurement
☐	☒	A statement on whether measurements were taken from distinct samples or whether the same sample was measured repeatedly
☐	☒	The statistical test(s) used AND whether they are one- or two-sided<br>*Only common tests should be described solely by name; describe more complex techniques in the Methods section.*
☐	☒	A description of all covariates tested
☐	☒	A description of any assumptions or corrections, such as tests of normality and adjustment for multiple comparisons
☐	☒	A full description of the statistical parameters including central tendency (e.g. means) or other basic estimates (e.g. regression coefficient) AND variation (e.g. standard deviation) or associated estimates of uncertainty (e.g. confidence intervals)
☐	☒	For null hypothesis testing, the test statistic (e.g. *F*, *t*, *r*) with confidence intervals, effect sizes, degrees of freedom and *P* value noted<br>*Give P values as exact values whenever suitable.*
☒	☐	For Bayesian analysis, information on the choice of priors and Markov chain Monte Carlo settings
☐	☒	For hierarchical and complex designs, identification of the appropriate level for tests and full reporting of outcomes
☒	☐	Estimates of effect sizes (e.g. Cohen's *d*, Pearson's *r*), indicating how they were calculated

Our web collection on statistics for biologists contains articles on many of the points above.

Software and code

Policy information about availability of computer code

Data collection	All code for data collection, including the code for running the experiment and demonstrations of the experiment, are publicly available on our Github repository: https://github.com/WeiJiMaLab/stochasticity_planning_effort. Release v1.0.0 is archived at Zenodo; concept DOI 10.5281/zenodo.22035888
Data analysis	All code for data analysis, including modeling, running significance tests, and generating figures, are publicly available on our Github repository: https://github.com/WeiJiMaLab/stochasticity_planning_effort. Release v1.0.0 is archived at Zenodo; concept DOI 10.5281/zenodo.22035888

For manuscripts utilizing custom algorithms or software that are central to the research but not yet described in published literature, software must be made available to editors and reviewers. We strongly encourage code deposition in a community repository (e.g. GitHub). See the Nature Portfolio guidelines for submitting code & software for further information.

Data

Policy information about availability of data

All manuscripts must include a data availability statement. This statement should provide the following information, where applicable:

- Accession codes, unique identifiers, or web links for publicly available datasets
- A description of any restrictions on data availability
- For clinical datasets or third party data, please ensure that the statement adheres to our policy

The anonymized, raw trial-level behavioural data generated in this study, comprising participant choices, response times, points earned, and stochasticity condition

for all 300 participants across the three experiments, have been deposited in the Open Science Framework (OSF) repository under https://doi.org/10.17605/OSF.IO/VYH8U, and are publicly available without restriction. The deposit contains the raw trial-level data, together with the derived cross-validation splits, and model fits needed to reproduce the analyses. The deposit does not include participants' individual-level demographic survey responses, which were collected only to describe the sample and are not used in any analysis, figure, or reported statistic; the aggregate demographics in the Methods are the only use made of them. They are withheld because participants did not consent to the release of individual-level demographic data. Every figure and table in this article and its Supplementary Information can be regenerated from these data using the analysis code (see Code availability), so no separate Source Data file is provided.

Research involving human participants, their data, or biological material

Policy information about studies with human participants or human data. See also policy information about sex, gender (identity/presentation), and sexual orientation and race, ethnicity and racism.

Reporting on sex and gender	The findings of our study are not specific to an individual sex or gender. We used the online data gathering platform Prolific to collect our data and designed our study to have a balanced gender cohort of English-speaking participants from the United States. Our work does not distinguish across sex and gender as we are looking for within-individual changes in planning strategy which we anticipated would generalize across sex and gender. Gender identity was collected for institutional reporting requirements, such data was anonymized and not used in the analysis for our study.<br><br>We collected self-reported age and gender but did not separately collect information about biological sex. In the reliability experiment (n = 100), the mean age was 36.9 years (SD = 11.2), with 50 male, 48 female, and 2 nonbinary participants. In the volatility experiment (n = 100), the mean age was 37.8 years (SD = 10.6), with 50 male, 48 female, and 2 nonbinary participants. In the controllability experiment (n = 100), the mean age was 38.0 years (SD = 10.6), with 50 male, 48 female, and 2 nonbinary participants. No sex or gender-based analysis was performed because the study was not designed to test for sex, gender, or individual differences in planning behavior.
Reporting on race, ethnicity, or other socially relevant groupings	In our study, we do not consider socially constructed categorization of variables or race, ethnicity, socially relevant groupings, or proxies thereof as a variable in our analysis. A brief survey on demographic information was collected for institutional reporting requirements, such data was anonymized and not used in our analysis for our study.
Population characteristics	No additional comments, see population details below.
Recruitment	Eligible participants needed to be at least 18 years old, fluent in English, and based in the United States. To maintain data quality, participants were automatically excluded if they failed two out of three attention checks, refreshed or restarted the experiment, or exited before completing the study. Prolific automatically timed out sessions exceeding 140 minutes. The analyzed dataset comprises all complete sessions that passed the attention checks and timeout conditions from Prolific. Each participant faced only one form of stochasticity within an experiment. Within each experiment, the order in which stochasticity levels were presented was randomized within each session and mirrored across its two halves to control for order effects.<br><br>The sample size was specified in advance in our pre-registration and was determined on the basis of an experimental pilot, whose data were not included in the analyses reported here; no statistical method was used to predetermine sample size. The experiments were not explicitly randomized: each was run as a separate Prolific study, and participants were not assigned to experiments on the basis of any characteristic. No data were excluded at the analysis stage; criteria were pre-established in the pre-registration.<br><br>The risk for self-selection bias is low; the only requirements for participation were a valid Prolific account and access to a computer and valid Chrome browser. We do not anticipate that this would systematically impact the planning ability or behavior of the selected participants.
Ethics oversight	The study was approved by the IRB of New York University.

Note that full information on the approval of the study protocol must also be provided in the manuscript.

Field-specific reporting

Please select the one below that is the best fit for your research. If you are not sure, read the appropriate sections before making your selection.

☐ Life sciences ☒ Behavioural & social sciences ☐ Ecological, evolutionary & environmental sciences

For a reference copy of the document with all sections, see nature.com/documents/nr-reporting-summary-flat.pdf

Behavioural & social sciences study design

All studies must disclose on these points even when the disclosure is negative.

Study description	This is a quantitative experimental study where we analyze how environmental stochasticity affects planning depth in humans. The study involves an online experiment and provides a quantitative account of planning depth through analysis of reaction times and computational cognitive modeling. Subjects include human participants recruited through an online experiment platform. The data collection, analysis, and study design was preregistered on OSF: https://osf.io/u6nfj
Research sample	Data was collected through Prolific with a gender-balanced cohort, selected out of English-speaking people in the United States who

	were at least 18 years old. The study was conducted online and participants could work on the task anywhere they preferred, so long as they had adequate access to reliable internet, a laptop or desktop, and a Google Chrome web browser. Beyond gender-balancing we did not establish quotas for other demographic features, as we focus on within-subject effort and do not anticipate nor test demographic-specific effects.
Sampling strategy	All recruitment and sampling was conducted through Prolific, subject to the constraints above. It is important to note that our study primarily involves within-participant comparisons; the randomization strategy across each stochasticity condition is described below. No statistical method was used to predetermine sample size. We pre-registered a target of 100 participants per experiment (300 in total) at https://osf.io/u6nfj, prior to data collection. That target was chosen on the basis of a separately collected experimental pilot, whose data are not included in these analyses.
Data collection	The entirety of the experiment was conducted online. Participants were required to use a laptop or desktop computer. Participants first completed a brief demographic survey before proceeding to the main experiment. Because the study was conducted online, the participants and experimenter were never face-to-face. All participant information was de-identified prior to analysis.<br><br>Data collected involved participant's choices, responses to questions, and key presses, as well as the timing of events (e.g. reaction times). Participants were provided with detailed step-by-step instructions, including diagrams illustrating the task setup. They also completed practice trials at each stochasticity level, which were excluded from the final analysis. Comprehension checks were added to verify their understanding of the task design and basic understanding of probability.<br><br>The main task consisted of a game where participants were asked to move left or right down a decision tree to gather points. Various manipulations of stochasticity were introduced to study how participants would respond.
Timing	All data was collected for this study between 06 August 2024 and 17 September 2024.
Data exclusions	To maintain data quality, participants were automatically excluded if they failed two out of three attention checks, refreshed or restarted the experiment, or exited before completing the study. Prolific automatically timed out sessions exceeding 140 minutes. Beyond these criteria, no additional data were excluded.
Non-participation	N/A - no participants declined to participate.
Randomization	All participants were registered through Prolific. We created three similar variants of the task and posted them concurrently online, and participants were not told in advance which variant they were assigned to. Participants were only allowed to participate in one of the three variants; no participant took part in more than one variant of the study. It is worth emphasizing that our results primarily focus on within-participant results, not across-participant manipulations.<br><br>Each participant experienced 5 different levels of stochasticity for their given variant. Each session consisted of 10 blocks, two for each stochasticity level. Each block contained 15 games. The stochasticity level remained constant within each block and was explicitly communicated to the participants. At the beginning of each block, participants confirmed the stochasticity level by pressing the corresponding number (1 for the lowest stochasticity and 5 for the highest). To control for order effects, the sequence of stochasticity levels was randomized and mirrored in the second half of the session. The true values of the treasure chests were randomly generated from a uniform distribution ranging from 1 to 9, inclusive. The stochasticity level was presented in multiple formats, as both a proportion and percentage (e.g. 3/4 and 75%).

Reporting for specific materials, systems and methods

We require information from authors about some types of materials, experimental systems and methods used in many studies. Here, indicate whether each material, system or method listed is relevant to your study. If you are not sure if a list item applies to your research, read the appropriate section before selecting a response.

Materials & experimental systems

n/a	Involved in the study
☒	☐ Antibodies
☒	☐ Eukaryotic cell lines
☒	☐ Palaeontology and archaeology
☒	☐ Animals and other organisms
☒	☐ Clinical data
☒	☐ Dual use research of concern
☒	☐ Plants

Methods

n/a	Involved in the study
☒	☐ ChIP-seq
☒	☐ Flow cytometry
☒	☐ MRI-based neuroimaging

Plants

Seed stocks	N/a
Novel plant genotypes	N/a
Authentication	N/a
