# Supplementary material for: Environmental stochasticity reduces human planning effort
Source: Nat Commun. 2026 Sep 28;17:11199. doi: 10.1038/s41467-026-78023-9 (PMC13714268; doi:10.1038/s41467-026-78023-9)
Supplement: Supplementary file 3 [file 41467_2026_78023_MOESM3_ESM.pdf]

nature portfolio

Peer Review File

Environmental stochasticity reduces human planning effort

Corresponding Author: Dr Jordan Lei

This file contains all reviewer reports in order by version, followed by all author rebuttals in order by version.

Version 0:

Reviewer comments:

Reviewer #1

(Remarks to the Author)
In this paper, Lei and colleague report 3 human behavioral experiments, along with computational modeling, which support the hypothesis that humans mitigate the intractability of planning by determinizing paths through a stochastic decision tree. The main behavioral finding is that people appear to be doing less planning as stochasticity increases, as measured by the first-choice response time.

I think this is an interesting and well-written paper. It addresses an important issue in realistic planning problems, and offers a quantitative account of how the human may address the issue. The experiments are carefully designed and the methods are rigorous.

I think the authors could do more with their data to provide behavioral evidence for the model assumptions. In particular, the only behavioral proxy for amount of planning is the first-choice RT, but the authors have the entire sequence of RTs. If people are planning to a limited depth, then there should be a sharp discontinuity in their RTs at that depth (i.e., where they need to plan again), assuming they ballistically follow the chosen path without replanning contingent on observed outcomes.

Another issue in relating the model to the behavior is that there are no response times in the model, and in fact it's not clear to me why the path-determinization strategy should produce different amounts of planning depending on stochasticity. I think what's going on here is that the model itself doesn't provide a fully endogenous explanation of planning depth as a function of stochasticity; this is captured by the free parameters. So in that sense it's a somewhat unsatisfying account.

From a computational perspective, I'm wondering whether the path-determinization strategy is really so useful in non-trivial domains; it still requires exhaustive enumeration of paths, and the number of such paths will increase exponentially with planning depth. I guess this is okay as long as the depth is limited (as in the winning model). It might be useful to readers to lay out explicitly what the different trade-offs are here.

(Remarks on code availability)

Reviewer #2

(Remarks to the Author)
The manuscript presents a set of experiments that illustrate how different forms of environmental stochasticity curb planning. Though there are related works, none has shown this effect, at least not as systematically and convincingly. The experiment and analyses are mostly well designed to answer the study question. And the conclusion is based both on reaction time data and computational modeling of choice. I think this manuscript makes a valuable contribution to the literature. However, I am worried that the models oversimplify what participants are actually doing, since they rely on unrealistic assumptions that people only use this or that heuristic, and that all people use the same heuristic. Another potentially important issue is that the G/LMM do not include random slopes, which is standard practice if one is to avoid false positive results. Below I detail specific comments and questions:

- There seems to be some systematic deviation in the posterior predictive checks at 0% stochasticity, which the text could flag. I guess some misfit is to be expected given the simplicity of the models and the assumption that all participants used the same heuristics. In this respect, it seems likely that people, either as a group or even as individuals, would use a mixture of

the proposed heuristics. For instance, looking only at a certain depth unless a '9' captures the eye beyond it. Many such combinations can be thought of. However, perhaps it is easiest and most important to fix the assumption of uniformity across the population. This can be achieved by creating hybrid models where each sub model gets a participant-level weight.

- Isn't the 'max' heuristic equivalent to filtering by rank 1?

- I am not sure it is accurate to say that all the determinizing heuristics act as if there is no stochasticity. The 'sum' heuristic can be understood as approximating the correct computation in the case of zero controllability. Low controllability (along with limited panning resources) is a key reason to consider outcomes that do not lie on the best path. This also suggests a more effective heuristic - averaging the values for each depth of the tree, and then summing these averages.

- The discussion with regards to epistemic uncertainty is key for understanding how the results apply or do not apply to real-world decision making. One thing to add here is that in many cases outcomes are deterministic but we are not willing or able to gather the necessary information. This is reflected even in the authors' first example of outdated recommendations. Presumably it is possible to collect new recommendations, but if a person is not willing or able to do so, then epistemic uncertainty may have the same effect of first-order uncertainty.

- The discussion could also link to known work about how uncertainty determines whether planning is initiated (Daw, Niv, & Dayan, 2005, Nat Neuro).

- The findings also cohere with a previous analysis of when it pays to plan performed by Kool, Cushman, & Gershman (2016). Several of the factors they identify as improving the payoff of planning reflect forms of stochasticity.

- It seems the G/LMMs do not include random slopes for the effects of interest. This carries the risk of substantially shrinking p values, and is usually considered invalid unless there is a reason not to include such random slopes.

- Perhaps I missed this, but I could not find model recovery and parameter recovery tests.

(Remarks on code availability)

Reviewer #3

(Remarks to the Author)
This article investigates how people plan ahead while traveling through stochastically rewarded paths. The authors find that more stochasticity in the rewards/paths leads to less behavioral sensitivity to the displayed rewards and to faster decisions. They fit some planning models and find that people only search a few steps into the future, ignoring the stochasticity in their calculations, but planning less far with more stochasticity.

Overall, my impression of the article is somewhat negative. While I appreciate the way in which the authors used variants on the same task to study different forms of stochasticity, I found the manipulation poorly motivated, the conclusions obvious, and the modeling under-developed. This feels like a good start to a project, not a complete story.

Throughout the paper, the distinction between unreliable and volatile is not well articulated. The examples in the introduction do not paint a clear picture. In the restaurant example, you arrive at a restaurant and discover that the food is worse than you remembered. In the weather example, you go for a walk and unexpectedly get rained on. Why is the first example about reliability and the second example about volatility? In the context of the task, the distinction is also not that clear. In the unreliable case, you choose a chest and instead get a random reward. In the volatile case, you choose a path and instead get random future rewards. The only difference is that in the unreliable case you experience the random reward immediately, while in the volatile case you have to wait one or more turns to collect, or avoid, the random reward. So what? Why is that difference important?

The behavioral results hypotheses/results strike me as quite obvious and would seemingly result from any model of costly cognitive effort. If your decisions have less impact on your outcomes, you should exert less effort in making the correct decisions. There are countless models and papers on this idea. What does the current paper add to the discussion?

In my view the only notable finding in the paper is that the depth of search varies with stochasticity. But again, what’s a plausible alternative? Given the empirical finding that people make quicker, less reward-sensitive decisions with more stochasticity, how else could the depth-path model account for that? Couldn’t it instead be that people use a depth-path model, but compute less precise approximations of the path values with increasing stochasticity? Basically, I’m not convinced that the authors’ models are anywhere near exhaustive, and they don’t seem to consider many ways in which the amount of stochasticity could degrade the estimates of the future paths.

Setting those issues aside, I still find the conclusions underwhelming. I was expecting to see something tying the different stochasticity manipulations together. Instead, we just learn that every manipulation leads to less depth of planning with increasing stochasticity. But how should we interpret the coefficients? Is this the ranking that we would expect? It seems that some manipulations might be worse than others. For instance, the reliability manipulation only affects a single reward at a

time, while the volatility manipulation affects multiple rewards at a time. So, you might expect volatility to have a larger effect on behavior than reliability. That does not seem to be the case. In any case, it seems important to include an analysis of the benefit of planning ahead in these different stochastic settings. That would really help improve the contribution here. It would also help with the connection to resource rationality, which the authors claim, without elaboration, is not supported by these data.

Minor comments:
Please include details of the instructions, practice trials, and comprehension checks. These details are important to evaluate how participants likely came up with strategies for this task.

How could participants continue planning/deciding while waiting for the animation to resolve? While that might be possible in the reliability case, it doesn't seem possible in the other cases.

(Remarks on code availability)

Version 1:

Reviewer comments:

Reviewer #1

(Remarks to the Author)
The authors have done a good job addressing my comments. The only remaining criticism I have (unrelated to my original comments but related to some of the new content) is that what they are calling "policy compression" isn't strictly speaking what that term refers to. They model changes in value sensitivity, which is indeed one aspect of policy compression. But another aspect is absent: the marginal policy (perseveration) term, which is needed to connect the model to compression in the information-theoretic sense. To be clear, I'm not suggesting that the authors have to add this term (though of course they could try that); I just think there needs to be a caveat somewhere that clarifies this point. The substantive reason it's important is that the marginal policy term is what separates policy compression from some form of generic value insensitivity, noise, or explore/exploit mechanism.

(Remarks on code availability)
Code is impressively thorough and well-documented.

Reviewer #2

(Remarks to the Author)
The authors thoroughly addressed all my comments, and I find the revised manuscript substantially stronger. The only remaining issue is that, to my understanding, the new random-effects Bayesian model selection procedure allows heterogeneity across but not within individuals, in the sense of an individual combining multiple heuristics. If my understanding is correct, I suggest that the authors at least note in the main text the possibility that participants used hybrid strategies.

The hypothesis and experimental design in the preregistration and manuscript are consistent. The analysis is also largely consistent. There are two minor changes: the method of model comparison has been changed (AIC/BIC/bootstrap in the prereg), and the method of measuring effects across stochasticity levels (ANOVA in the prereg) - in my view these changes merely need some justification. Additionally, there are some extra analyses added whether due to the authors or reviewer requests (e.g. reaction time analyses) that should be labeled exploratory.

(Remarks on code availability)

Reviewer #3

(Remarks to the Author)
The authors have largely addressed my concerns and I think that the article is much improved. I just have a few small lingering concerns.

(1) It isn't obvious, at least to me, that the resource rationality necessarily means using the expected value strategy. It seems like another approach could yield higher expected gains for the same amount of effort. It would be helpful if the authors could elaborate on this point and clarify why they think that expected value maximization is always optimal.

(2) I appreciate the model recovery exercise, but I think it is incomplete. We also need to know whether other models are correctly recovered when they are the true data generating process. Otherwise it is possible that other data generating processes could be mistaken as evidence for PC Depth-filtering Level-Mean or PC Depth-filtering Path.

(Remarks on code availability)

Version 2:

Reviewer comments:

Reviewer #2

(Remarks to the Author)
The authors adequately addressed all my remaining concerns.

(Remarks on code availability)

Reviewer #3

(Remarks to the Author)
The authors have addressed my remaining comments.

(Remarks on code availability)

Open Access This Peer Review File is licensed under a Creative Commons Attribution 4.0 International License, which permits use, sharing, adaptation, distribution and reproduction in any medium or format, as long as you give appropriate credit to the original author(s) and the source, provide a link to the Creative Commons license, and indicate if changes were made.
In cases where reviewers are anonymous, credit should be given to 'Anonymous Referee' and the source.
The images or other third party material in this Peer Review File are included in the article's Creative Commons license, unless indicated otherwise in a credit line to the material. If material is not included in the article's Creative Commons license and your intended use is not permitted by statutory regulation or exceeds the permitted use, you will need to obtain permission directly from the copyright holder.
To view a copy of this license, visit https://creativecommons.org/licenses/by/4.0/

Overview of Key Revisions

We thank all reviewers for their constructive and thoughtful feedback. In response, we have undertaken a major revision of the manuscript, incorporating new analyses, conceptual extensions, and clarifications throughout. Below, we summarize the most substantial changes and how they strengthen the paper.

Conceptual Revision: Policy Compression versus Filter Adaptation

The most significant conceptual change is the introduction of Policy Compression (PC) as a mechanism for adapting planning effort. Our original analysis focused on Filter Adaptation (FA), in which participants adjust how much information they consider when planning. Inspired by Reviewer 3's suggestion, we expanded this framework to test whether participants instead adjust their sensitivity to value differences—a process formalized as Policy Compression in the resource rationality literature. We re-ran our analysis pipeline, doubling the number of models to compare FA and PC mechanisms. The best fitting models used policy compression, depth-filtering, and determinizing value functions, resolving systematic biases in low-stochasticity conditions (which were pointed out by Reviewer 2) and providing a more unified account of planning effort.

These new results reframe our main conclusion: rather than showing that participants simply plan less deeply under higher stochasticity, we now demonstrate that they compress their policies—reducing decision sensitivity in a manner consistent with resource-rational theories of cognitive effort. The policy compression framework provides a principled explanation for how people allocate cognitive resources during planning.

New Normative Analysis: Resource-Rational Simulations

In response to Reviewer 1's request for an endogenous rationale linking stochasticity to planning effort, we conducted a new set of resource-rational simulations. These analyses simulate how rational agents allocate effort across stochasticity levels when trading off expected reward against the cost of policy compression. The simulations revealed a normative pattern that aligns with the empirical results: optimal planning sensitivity ($\log \beta$) decreases as environmental stochasticity increases. This occurs because the marginal benefit of precise planning diminishes when future outcomes are more uncertain. This analysis strengthens the theoretical grounding of our framework and demonstrates that reduced planning effort under higher stochasticity arises naturally from a reward–effort trade-off.

Expanded Behavioral Analyses: Total Response Times

In response to the reviewer comment, we conducted an analysis of total response times across the entire game sequence rather than only the first-move latencies (Supplementary Section S1). Total RTs decrease systematically with increasing stochasticity,

replicating the key behavioral effect across an independent measure. These confirm that participants invest less time and cognitive effort under higher uncertainty (Supplementary Fig. S1).

Improved Robustness: Mixed Models Analysis and Model Validation

We have completely re-run all hierarchical and mixed-effects analyses with maximal random-effects structures, following the recommendations of Reviewer 2. Our new analysis now adheres to best practices, starting with G/LMMs with maximal random effects and iteratively winnowing random effects upon singular fits. In addition, we included comprehensive model recovery and parameter recovery analyses, demonstrating that the model parameters and structure are recoverable under simulated data. These additions improve both the robustness and interpretability of our modeling framework.

Clarifications and Theoretical Extensions

In response to various reviewer comments, we have clarified several conceptual assumptions and theoretical connections throughout:

- **Conditional independence:** Explicitly stated in the Methods and Results to clarify that participants were modeled as replanning at each step rather than following precommitted sequences.
- **Scalability and pruning:** We expanded the discussion to link our framework to *pruning* and bounded-lookahead models [1–3], emphasizing that determinization and pruning represent complementary strategies for managing intractable planning.
- **Integration of new methods:** We explain how Filter Adaptation and Policy Compression capture distinct but complementary mechanisms of effort allocation, and why Policy Compression better accounts for our data.
- **Connections to existing theory:** The revised Discussion now explicitly integrates work on rational inattention [4, 5], uncertainty-based control [6], and ecological rationality [7].

Summary

Together, these changes yield a clearer and more principled account of our central conclusion: people adaptively reduce their planning effort under stochasticity via policy compression. Over the course of this revision, we conducted a comprehensive new set of analyses, including expanded behavioral measures, a significantly expanded model space comparing Filter Adaptation and Policy Compression mechanisms, and resource-rational simulations that provide a normative foundation for these effects. We find that in response to stochasticity, participants compress their policies, becoming less sensitive to value differences as environmental stochasticity increases. The revised manuscript now links this behavioral pattern to a normative, resource-rational principle and validates it across multiple behavioral and computational measures. We believe

these additions substantially strengthen the theoretical coherence and contribution of the work, and we are deeply grateful to the reviewers for strengthening this work.

In the following sections, we address each reviewer's comments thematically, highlighting reviewer notes in blue.

1 Reviewer 1 Comments

1.1 Behavioral Measures of Effort and Response Times

I think the authors could do more with their data to provide behavioral evidence for the model assumptions. In particular, the only behavioral proxy for amount of planning is the first-choice RT, but the authors have the entire sequence of RTs.

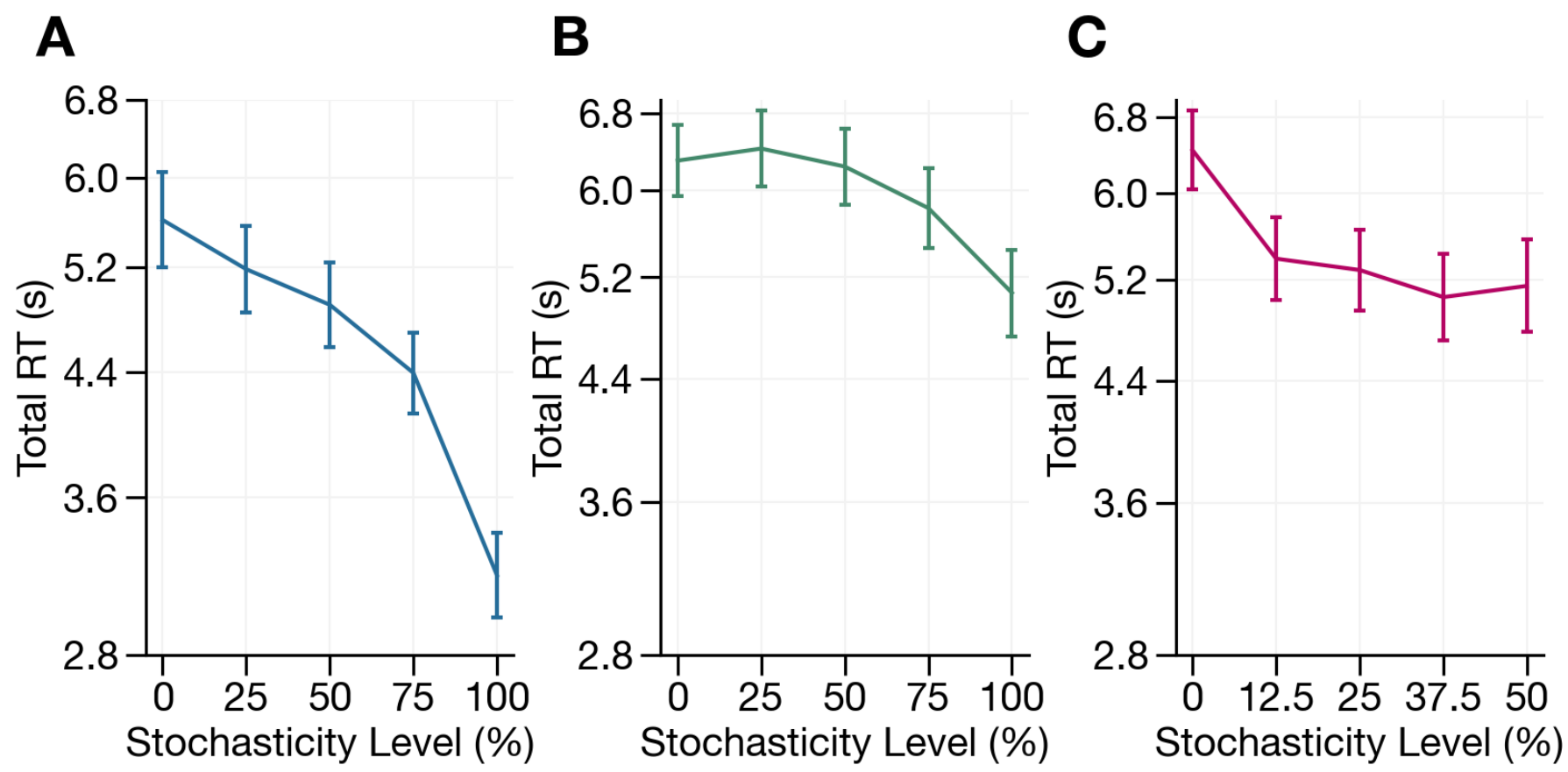

Fig. 1 Total response time results. **A - C.** Total response times as a function of stochasticity level, plotting on a log-scaled y-axis.

We followed the Reviewer 1's comment to conduct a new, more comprehensive analysis examining participants' full sequences of moves across all trials.

Our main text focuses on first-move response times, as we found that participants spent substantially more time deliberating on the first move compared to subsequent moves. However, we agree that examining the full trajectory provides valuable additional insight into how participants allocate effort throughout the game.

Consistent with the model's predictions, we find that (log-transformed) total response times decrease monotonically with increasing stochasticity (Fig. 1, Reliability LMM, $\beta = -0.52$, $t_{98} = -7.97$, $p < 10^{-10}$; Volatility LMM, $\beta = -0.21$, $t_{99} = -3.95$, $p = 1.5 \times 10^{-4}$; Controllability LMM, $\beta = -0.41$, $t_{98} = -4.17$, $p = 6.5 \times 10^{-5}$). This new analysis therefore provides convergent behavioral evidence, beyond the first choice, that participants exert less cognitive effort when the environment becomes less predictable, strengthening the link between our behavioral and modeling results.

1.2 Model Assumptions – Conditional Independence

If people are planning to a limited depth, then there should be a sharp discontinuity in their RTs at that depth (i.e., where they need to plan again), assuming they ballistically follow the chosen path without replanning contingent on observed outcomes.

We thank the reviewer for this comment as it made us realize we were not sufficiently clear about our conditional independence assumptions in the main text. Specifically, our models assume that each choice is conditionally independent of previous choices given the current state, meaning that participants evaluate their options at every step rather than commit to a fixed multi-step plan. We have added the following clarification in the main text:

> "Our models predicted participants' choices at each decision point, assuming that each choice was guided by the subjective values assigned to the left and right subtrees of the decision tree. The greater the difference in these values, the more likely participants were to select the higher-valued option. We assumed conditional independence across choices, such that each decision depended only on the values of the immediately available subtrees."

This conditional independence assumption is widely used in the planning and decision-making literature and allows for tractable computation of choice probabilities while still capturing dynamic replanning behavior. Because our task requires participants to adapt to newly revealed or resampled information, particularly in the controllability and volatility conditions, ballistic or precommitted planning would not be possible in all conditions. We therefore would not expect to see sharp discontinuities at specific depths.

1.3 Normative Rationale and Policy Compression

Another issue in relating the model to the behavior is that there are no response times in the model, and in fact it's not clear to me why the path-determinization strategy should produce different amounts of planning depending on stochasticity. I think what's going on here is that the model itself doesn't provide a fully endogenous explanation of planning depth as a function of stochasticity; this is captured by the free parameters. So in that sense it's a somewhat unsatisfying account.

We thank the reviewer for this thoughtful comment, which motivated us to substantially expand our analysis to consider possible endogenous accounts of planning effort. While our main text focuses on descriptive models that characterize how participants actually planned, this comment encouraged us to explore a normative, resource-rational perspective—one that explains *why* planning effort should vary systematically with environmental stochasticity.

We explored this idea using simulations. In these simulations, we treated planning effort itself as the outcome of an internal optimization process balancing costs and benefits. Specifically, greater planning effort (operationalized as higher β, i.e., greater sensitivity to value differences) improves expected rewards but incurs increasing cognitive cost. To formalize this tradeoff, we conducted simulations across a wide range of β values, estimating the expected utility at each level of effort. We then modeled the choice of β as an optimization over this utility function, providing an explicit normative account of how planning sensitivity should adapt to task structure.

We found that the optimal β decreases monotonically with increasing stochasticity, mirroring the empirical trends reported in the main text. These results suggest that the observed modulation of effort by stochasticity is normatively consistent with a reward–cost tradeoff. We emphasize that these simulations are illustrative rather than definitive; they show that a resource-rational tradeoff could give rise to the observed patterns, rather than claiming that participants implement this computation explicitly.

Together, these findings provide a theoretical complement to the descriptive policy-compression framework presented in the main text. The main text analysis shows that participants expend less effort, reflected in both reduced response times and lower inferred β, as environments become more stochastic. The new simulations show that this pattern can arise naturally from cost–benefit optimization, thereby providing a normative rationale for the descriptive model's parameters. Moreover, policy compression has been previously linked to response times [8], and our results extend this relationship by demonstrating that the same principles can explain adaptive planning effort as a function of environmental stochasticity.

1.4 Extensions to More Complex Environments

> From a computational perspective, I'm wondering whether the path-determinization strategy is really so useful in non-trivial domains; it still requires exhaustive enumeration of paths, and the number of such paths will increase exponentially with planning depth. I guess this is okay as long as the depth is limited (as in the winning model). It might be useful to readers to lay out explicitly what the different trade-offs are here.

We appreciate this insightful comment, which prompted us to more explicitly articulate the computational trade-offs inherent in our framework and to situate our modeling approach within a broader landscape of planning strategies. In response, we expanded our discussion in the main text to clarify both the assumptions and the limitations of path-based determinization, as well as how such strategies might scale in more complex environments:

> We do not intend to suggest that participants universally ignore expected value considerations when simplifying their planning, nor that they necessarily do so by means of the Path or Level-Mean algorithms. In our task, these determinizing heuristics were both simple and well-matched to the problem structure, likely because the branching factor at each decision point was small and the overall state space was tractable. In more complex environments, where each choice may open many possible continuations or the state space grows very large, we would expect participants to adopt alternative strategies — such as pruning [1–3] — to manage their computational load.

This clarifies that path-based determinization might serve as an effective heuristic only under restricted conditions—specifically, when planning depth and branching factor remain within cognitively manageable bounds. Beyond those limits, people might rely on hybrid strategies (e.g., pruning, partial evaluation, or hierarchical decomposition) that selectively ignore or collapse parts of the decision tree to maintain tractability.

We see this clarification as an opportunity to connect our work to the broader literature. It highlights that our models should be viewed as points along a continuum of resource-rational strategies, where individuals adaptively trade off precision for computational efficiency. By explicitly acknowledging these trade-offs, we connect our

findings to a growing literature showing that planning strategies scale adaptively with task complexity and cognitive cost, thereby situating our approach within a broader theory of bounded, adaptive planning.

2 Reviewer 2 Comments

2.1 Model Simplifications and Participant Variability

I think this manuscript makes a valuable contribution to the literature. However, I am worried that the models oversimplify what participants are actually doing, since they rely on unrealistic assumptions that people only use this or that heuristic, and that all people use the same heuristic.

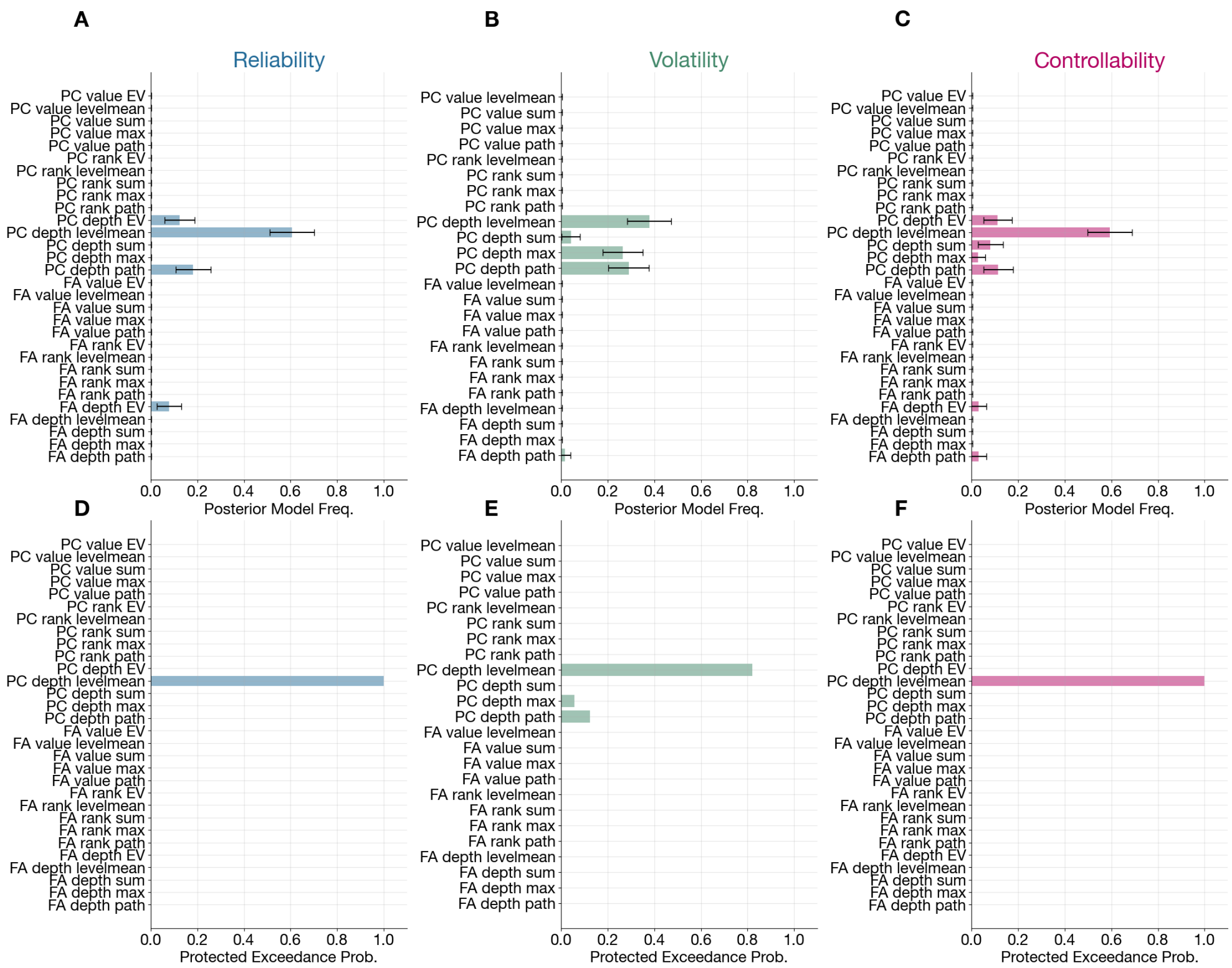

Fig. 2 Random-Effects Bayesian Model Comparison. **A–C.** Posterior frequencies for each model. Error bars represent 95%CI. **D–F.** Protected Exceedence probabilities for each model.

We thank the reviewer for this thoughtful point. We fully agree that individuals may rely on different planning strategies. To directly address this, we conducted a new random-effects Bayesian model comparison [9] to quantify heterogeneity across participants. This analysis revealed that the PC Depth-filtered Level–Mean model was the most prevalent strategy in the population, with high posterior model frequencies and significant protected exceedance probabilities in both the reliability and controllability conditions (Fig. 2).

Importantly, we also evaluated competitive alternative models (Supplementary Fig. S3–S5) and found that they produced the same qualitative conclusions: people plan using depth-limited policy compression and determinizing value functions.

Taken together, these results show that while individuals vary in their specific planning heuristics, the core theoretical conclusions are robust across modeling approaches and participant-level heterogeneity.

2.2 G/LMM Random Slopes

> Another potentially important issue is that the G/LMM do not include random slopes, which is standard practice if one is to avoid false positive results ... It seems the G/LMMs do not include random slopes for the effects of interest. This carries the risk of substantially shrinking p values, and is usually considered invalid unless there is a reason not to include such random slopes.

We are grateful to the reviewer for raising this important methodological point. In response, we re-ran all generalized and linear mixed-effects models (G/LMMs) reported in both the main text and Supplement, incorporating random slopes wherever justified by the design. Each model was initially fit with the *maximal random-effects structure* recommended by best-practice guidelines [10] and then simplified only when necessary due to convergence or singular fit issues. This required refitting and revalidating every analysis in the paper to ensure that our conclusions were fully robust to model specification.

The revised Methods now read as follows:

> "All models were first fit with the maximal random-effects structure justified by the design, including by-participant random intercepts and random slopes for all predictors of interest. If the maximal model failed to converge or yielded a singular fit, we simplified the random-effects structure stepwise. First, we removed correlations among random intercepts and slopes while retaining all random slopes. If this uncorrelated slopes model also failed, we further reduced the model to include only random intercepts. Finally, if this also failed to fit, we reduced the model to include only fixed effects. All reported results are based on the most complex model that converged reliably, adhering to best-practice recommendations for mixed-effects analyses."

Importantly, these reanalyses did not qualitatively alter any of our main findings. Across all updated models, stochasticity remained associated with decreased (log) first-move response times, and participants continued to show reduced planning effort (measured by β) with increasing stochasticity. Thus, our primary effects are robust even under stricter random-effects specifications, further strengthening confidence in the reliability and generality of our results.

2.3 Low Stochasticity Deviations

> - There seems to be some systematic deviation in the posterior predictive checks at 0% stochasticity, which the text could flag. I guess some misfit is to be expected given the simplicity of the models and the assumption that all participants used the same heuristics. In this respect, it seems likely that people, either as a group or even as individuals, would use a mixture of the proposed heuristics. For instance, looking only at a certain depth unless a '9' captures the eye beyond it. Many such combinations can be thought of. However,

perhaps it is easiest and most important to fix the assumption of uniformity across the population. This can be achieved by creating hybrid models where each sub model gets a participant-level weight.

We thank the reviewer for raising this point. As noted in our response above, we now explicitly examine heterogeneity in strategies using a random-effects Bayesian model comparison (RFX-BMC), which allows different participants to favor different heuristics. This analysis shows that the PC Depth-filtered Level–Mean model is the most prevalent strategy in the population, while competitive alternatives support the same qualitative results (Supplementary Fig. S3–S5).

We also now note in the text the small but systematic deviation at 0% stochasticity in the original model checks. As the reviewer suggests, this pattern is consistent with occasional use of deeper or more opportunistic lookahead (e.g., selectively inspecting large values). Importantly, our updated policy-compression implementation substantially reduces this deviation (Fig. 5A–C), and the core qualitative conclusions are unchanged across models that capture different planning heuristics.

Together, these additions clarify that modest misfit at 0% stochasticity reflects expected variability in planning behavior, and our results remain robust to individual differences in strategy use.

2.4 Model Extensions (Level-Mean)

This also suggests a more effective heuristic - averaging the values for each depth of the tree, and then summing these averages.

We thank the reviewer for this thoughtful and constructive suggestion. It directly inspired us to develop and implement a new value function, the *Level–Mean* heuristic, which averages rewards at each depth of the tree and sums these level–wise means. We integrated this heuristic into our full computational modeling pipeline and re–ran all model fitting and comparison analyses. As now described in the main text:

> "**Level-Mean:** a coarser deterministic heuristic [than the Path heuristic] that averages rewards at each level of the tree and sums these level-wise means, approximating subtree value without tracking specific trajectories ... participants approximate value by averaging rewards at each depth of the tree and summing these level-wise means. This provides a coarse, low-cost summary of tree structure without enumerating paths, serving as a simpler heuristic to the Path model."

This new model proved highly successful. The PC Depth-filteringLevel–Mean model emerged as the best–fitting model across conditions in our random–effects Bayesian model comparison (Supplementary Fig. S2), outperforming the previous winning model while preserving the same theoretical predictions. We found that increasing stochasticity robustly decreased $\log(\beta)$ across all three conditions (Reliability LMM, $\beta = -1.71$, $t_{98} = -9.11$, $p < 10^{-10}$; Volatility LMM, $\beta = -1.80$, $t_{98} = -8.60$, $p < 10^{-10}$; Controllability LMM, $\beta = -4.11$, $t_{98} = -14.3$, $p < 10^{-10}$). Full posterior predictive checks likewise demonstrate close alignment between this model and human behavior. We are grateful for this suggestion as it led to a simpler, more cognitively plausible model that ultimately provides the strongest account of participant behavior in our task.

2.5 Conceptual Clarifications

2.5.1 Max Heuristic vs Filtering by Rank 1

- Isn't the 'max' heuristic equivalent to filtering by rank 1?

We thank the reviewer for raising this point, which prompted us to clarify the conceptual distinction between *filter operations* and *value functions* in our framework. In our initial submission, this distinction may not have been sufficiently explicit, leading to the impression that rank-based filtering and value-based heuristics perform the same operation. In the revised manuscript, we have expanded the text to explain that filters and value functions serve distinct roles: a filter operation F selects or masks parts of the decision tree (e.g., by depth, value, or rank), whereas a value function V assigns a single scalar value to the filtered subtree. We have revised the main text to emphasize this distinction:

> "Given the environment's complexity, it is unlikely that participants evaluated all available information. Instead, we hypothesized that they employed *filtering* to focus on specific aspects of the tree ... After filtering, participants must assign scalar values to the left and right subtrees. We considered several *value functions* that vary in computational sophistication and cognitive effort"

We also made substantive updates to the Methods section to highlight these definitions:

> "We consider three broad classes of filtering strategies—*depth-filtered*, *value-filtered*, and *rank-filtered* models—each capturing a distinct way participants might limit their planning horizon or attentional scope. In all cases, a filter function F selects a subset of the state s based on a parameter f (which may correspond to depth d, value threshold v, or rank cutoff k) and sets the remaining values to zero ... After filtering, a value function $V(s, q)$ maps the filtered state to a scalar estimate of expected reward. We consider several possible value functions, which differ in how they approximate the utility of the filtered subtree."

We illustrate by example below that the behavior of a participant would differ if you took an unfiltered tree and performed the max operation compared to if you took the filtered tree and performed any of the heuristic operations on the resultant tree. Consider the following tree:

```
      3
     / \
    8   5
   / \ / \
  8   1   7
```

In this scenario, without any filtering, the "max" heuristic would see that the left subtree (8 - 8 - 1) has a maximum value of 8 and the right subtree (1 - 5 - 7) has a maximum value of 7, and therefore the value of moving left would be $8 - 7 = 1$

If we filtered by rank 1 instead, we would set all the values of the tree that are below rank 1 (the value 8) to zero, effectively removing them from consideration. The tree would look like the following after filtering:

```
      0
     / \
    8   0
```

```
 / \ / \
8   0   0
```

In this case, the participant only "sees" the value 8 and all the other values are set to 0.

The filtered state changes the inputs to all subsequent value functions. Applying the same heuristics to the filtered tree produces qualitatively different decision variables (e.g., for max: $8-0 = 8$; for path: $16-0 = 16$). This example demonstrates that filtering and valuation are independent operations, and that filtering by rank 1 followed by a heuristic is not equivalent to simply applying the max heuristic to the unfiltered tree.

2.5.2 Sum Heuristic as an Approximation to Zero Controllability

- I am not sure it is accurate to say that all the determinizing heuristics act as if there is no stochasticity. The 'sum' heuristic can be understood as approximating the correct computation in the case of zero controllability. Low controllability (along with limited panning resources) is a key reason to consider outcomes that do not lie on the best path.

This is a subtle point and requires a little bit of untangling. We agree that the sum heuristic can be understood to be a (very) coarse approximation of the optimal computation in the course of zero controllability; however, such an approximation is mathematically incorrect, ignores the transition structure, and nonetheless still represents a *deterministic surrogate* of stochasticity.

Once again we illustrate with an example. Suppose we are considering the value of a subtree (because this is a heuristic, it operates on some subtree of the full tree, not shown here) of depth 3:

```
    1
   / \
  1   1
 / \ / \
1   9   2
```

This subtree of depth 3 yields four possible paths: 1–1–1, 1–1–9, 1–1–9, and 1–1–2. Under zero controllability (50% stochasticity), all paths are equally likely, giving an expected value of $0.25 \times (3 + 11 + 11 + 4) = 7.25$.

The *sum* heuristic, by contrast, simply adds all node values ($1+1+1+9+2 = 14$), ignoring that the upper nodes contribute to multiple paths while the terminal nodes differ in frequency. Because it neglects the transition probabilities that determine how often each node contributes to the expected value, it therefore cannot be considered a valid computation of the uncontrollable case.

2.6 Broader Theoretical Connections

2.6.1 Connections to Epistemic Uncertainty

- The discussion with regards to epistemic uncertainty is key for understanding how the results apply or do not apply to real-world decision making. One thing to add here is that in many cases outcomes are deterministic but we are not willing or able to gather the necessary information. This is reflected even in the authors' first example of outdated recommendations. Presumably it is possible to collect new recommendations, but if a

person is not willing or able to do so, then epistemic uncertainty may have the same effect of first-order uncertainty.

We thank the reviewer for this insightful comment and fully agree that the distinction between epistemic and first-order (outcome) uncertainty is critical for understanding how our results extend to real-world decision-making. Following this suggestion, we expanded the discussion to clarify that our framework applies not only to situations where outcomes are inherently stochastic, but also to those where uncertainty arises from limited access to or unwillingness to seek new information. This conceptual extension helps bridge our task design to more naturalistic forms of uncertainty.

The revised discussion now reads:

> "We would expect our findings to generalize to cases where it is possible *in principle* to obtain information but doing so is difficult or infeasible; in the example in our discussion of outdated recommendations, it may be possible to obtain more recent recommendations but doing so might be difficult in the moment or prohibitively costly."

This addition explicitly links epistemic uncertainty to the same cost–benefit trade-offs that underlie our modeling framework, extending the applicability of our findings to real-world planning scenarios.

- The discussion could also link to known work about how uncertainty determines whether planning is initiated (Daw, Niv, & Dayan, 2005, Nat Neuro).

We agree that this citation strengthens the paper. The article pairs nicely with our discussion about how in uncertain environments, planning may become worthwhile as a way of reducing uncertainty over outcomes. We have added this citation to our existing discussion of epistemic uncertainty:

> However, in other epistemically uncertain environments, planning more deeply may become more valuable as the act of planning may reduce uncertainty over outcomes [2, 6].

2.6.2 When it Pays to Plan

- The findings also cohere with a previous analysis of when it pays to plan performed by Kool, Cushman, & Gershman (2016). Several of the factors they identify as improving the payoff of planning reflect forms of stochasticity.

We thank the reviewer for highlighting this important connection. We agree that our results align closely with the framework established by [7] who analyzed the computational circumstances under which planning is advantageous. In response, we have expanded our discussion to explicitly situate our findings within this "when-it-pays-to-plan" perspective and to clarify how our modeling framework provides a quantitative complement to that theoretical analysis.

> "Our findings are qualitatively consistent with predictions of resource rationality, suggesting that people weigh the cognitive costs of planning against the potential benefits when deciding whether and how far to plan ahead. Existing work using simulated planning suggests that features like stochastic transitions and reward functions might diminish the utility of model-based planning relative to habit [7]. Additionally, policy compression

has been directly linked to resource-rational concepts of attention allocation [5]. Combining these principles, we show quantitatively that the effect of stochasticity on policy compression can be explained by the reward-cost tradeoff ...”

This addition both contextualizes our results within a well-established theoretical framework and clarifies how our models provide a direct computational realization of the trade-off between expected reward and cognitive effort described in prior work. Moreover, it provides a conceptual bridge to our supplementary analysis, where we explicitly optimize the utility of $\log(\beta)$ across stochasticity levels, demonstrating that similar cost–benefit principles identified naturally emerge from our framework.

2.7 Model and Parameter Recovery

- Perhaps I missed this, but I could not find model recovery and parameter recovery tests.

We thank the reviewer for pointing this out. We have now added both model-recovery and parameter-recovery analyses to the Supplement (Supplementary Section S4). For model recovery, we simulated data under each candidate model and refit all models using our full pipeline. The PC Depth–Level–Mean and PC Depth–Path models were reliably recovered across stochasticity conditions, confirming that the models are identifiable under realistic data regimes.

We also conducted parameter-recovery analyses using synthetic data generated under each model. Across depth, lapse, and inverse-temperature parameters, the recovered estimates closely tracked the ground-truth values (Supplementary Fig. S11), demonstrating robust identifiability of key parameters.

Together, these analyses confirm that our fitting procedure successfully recovers both model identity and underlying parameters, providing strong support for the reliability of our modeling framework.

3 Reviewer 3 Comments

3.1 Conceptual Motivation

> Overall, my impression of the article is somewhat negative. While I appreciate the way in which the authors used variants on the same task to study different forms of stochasticity, I found the manipulation poorly motivated, the conclusions obvious, and the modeling under-developed. This feels like a good start to a project, not a complete story.

We thank Reviewer 3 for their thoughtful feedback. While we have a different perspective on the impact of our work, we greatly appreciate the opportunity to clarify our motivation and substantially strengthen both the modeling framework and theoretical grounding of the manuscript in response to these points.

Since receiving the review, **we expanded our model space** to address concerns that the original modeling framework was under-developed. Specifically, we introduced additional value functions (including the new *Level-Mean* heuristic) and incorporated a novel class of models—motivated by Reviewer 3's feedback—that modulate planning *sensitivity* rather than *depth*. This alternative mechanism proved consistently superior to depth-filtering models. These results reinforce our central conclusion: participants reduce planning effort under higher stochasticity primarily by decreasing sensitivity to value differences, rather than by shortening their planning horizon. Importantly, this pattern held across all three stochasticity manipulations, resolving discrepancies in earlier posterior predictive checks and demonstrating that the effect generalizes across distinct uncertainty structures.

We also strengthened the theoretical motivation for our manipulations and clarified their connection to real-world planning challenges. Each form of stochasticity in our design (reliability, volatility, and controllability) corresponds to a class of uncertainty encountered in everyday decision-making, such as unpredictable outcomes, changing contingencies, or limited control over actions. Including these distinct manipulations was crucial for demonstrating the robustness and generality of our main effect. We have revised the Discussion to situate our new findings within the broader framework of resource-rational planning:

> "This strategic simplification is a core feature of policy compression, a mechanism that allows agents to balance policy complexity and reward maximization [8, 11]. This tradeoff is often framed as an information bottleneck, where a greater β corresponds to a larger information budget for implementing more complex and cognitively expensive policies [4, 5, 8, 11, 12]. Policy compression has been demonstrated in humans performing complex planning tasks [11], and emerging evidence suggests that midbrain dopamine signals are responsive to policy complexity [13]. Additionally, the principle of policy compression has been used to design more robust and resource-efficient artificial agents [14]. Our work adds to this rich domain by identifying environmental stochasticity as a key factor that drives policy compression dynamically."

To make this connection even more explicit, **we conducted a new set of resource-rational simulations** demonstrating that the observed decrease in policy sensitivity ($\log(\beta)$) with increasing stochasticity arises naturally from optimizing expected utility under cognitive cost constraints. Critically, this normative prediction held across all three stochasticity manipulations, mirroring the behavioral results.

To ground the motivation behind our work, we conducted a new set of simulations demonstrating that resource rationality predicts the normative effects that we see in the data. Based on our simulations, we find that a resource-rational agent would choose to diminish their sensitivity $\log(\beta)$ with increased stochasticity *across all three of our manipulations*, which is exactly what we find.

Finally, **we expanded the Discussion to incorporate cases where increased planning effort may instead be adaptive**, such as those involving epistemic uncertainty, emphasizing the fact that our conclusions are not trivial. This addition clarifies the boundary conditions of our account and helps reconcile our findings with real-world cases where uncertainty might instead *increase* planning effort:

> "We would expect our findings to generalize to cases where it is possible *in principle* to obtain information but doing so is difficult or infeasible; in the example in our discussion of outdated recommendations, it may be possible to obtain more recent recommendations but doing so might be difficult in the moment or prohibitively costly.However, in other epistemically uncertain environments, increasing planning effort or policy sensitivity may become more valuable as the act of planning may reduce uncertainty over outcomes [2, 6]."

3.2 Policy Compression as an Alternative Explanation

> Given the empirical finding that people make quicker, less reward-sensitive decisions with more stochasticity, how else could the depth-path model account for that? Couldn't it instead be that people use a depth-path model, but compute less precise approximations of the path values with increasing stochasticity? ... Basically, I'm not convinced that the authors' models are anywhere near exhaustive, and they don't seem to consider many ways in which the amount of stochasticity could degrade the estimates of the future paths.

We thank the reviewer for highlighting this alternative class of models. Rather than adapting planning depth (i.e., changing how much of the tree is filtered as stochasticity increases), this proposal suggests that participants may instead reduce the precision of their value computations — a mechanism closely related to *policy compression* and rational inattention. We found this insight compelling and reshaped our modeling framework in response.

In our revision, we more than doubled our model space to compare our original filter-adaptation models to policy-compression models in which planning effort is regulated through sensitivity to value differences rather than depth. We re-fit the full dataset with this expanded model suite. Surprisingly, the best-fitting models across all three stochasticity manipulations were the PC Depth-filtered Path and PC Depth-filtered Level-Mean models. This finding preserves our original qualitative conclusion that planning effort decreases as stochasticity increases, but reveals a new mechanism: rather than shortening their planning depth, participants appear to become *less sensitive* to value differences under higher stochasticity. This result aligns with prior work demonstrating that policy compression is associated with reductions in deliberation time [8], and provides a richer account of adaptive effort allocation in stochastic environments.

3.3 Connection to Resource Rationality

It would also help with the connection to resource rationality, which the authors claim, without elaboration, is not supported by these data.

We appreciate Reviewer 3's comment, as it helped us clarify the relationship between our findings and resource rational accounts of planning. We believe the concern reflects two possible interpretations, and we address both below.

First, Reviewer 3 may be concerned that our data do not support a resource rational interpretation. To address this, we conducted a **new resource rational simulation analysis** (Supplementary Section S3). In this analysis, we treat the selection of $\log(\beta)$ as a meta-decision: simulated agents choose their planning sensitivity to maximize a utility function trading off expected reward against cognitive cost. Consistent with our empirical results, the utility-maximizing $\log(\beta)$ decreases with increasing stochasticity across all three manipulations. These simulations suggest that the pattern we observe arises naturally from optimizing a reward–effort tradeoff, strengthening the link between our empirical findings and resource-rational principles.

Second, Reviewer 3 may have interpreted our discussion as implying that our results contradict resource rationality. This would be a misunderstanding; our findings deviate from strict resource rational optimality, but do not contradict the theory. To clarify, we added the following text to the Discussion:

> "While our findings broadly align with a resource-rational framework, they diverge from strict resource rationality. A fully resource-rational strategy assumes that people choose how much effort to exert and then act optimally given that effort. Under this framework, we would expect participants to follow the expected-value model, which explicitly calculates expected future rewards. Instead, they adopted a simpler heuristic, treating rewards as deterministic even when computing true expected values was feasible."

In other words, our results support the core idea of resource-rationality—people allocate effort adaptively—while highlighting that they do so using simplified heuristics rather than implementing the expected-value computation implied by strict optimality. We have revised the manuscript to make this distinction explicit and to better articulate how our normative analysis connects to the empirical findings.

3.4 Stochasticity Manipulations

3.4.1 Clarifying the Role of Volatility

Throughout the paper, the distinction between unreliable and volatile is not well articulated. The examples in the introduction do not paint a clear picture. In the restaurant example, you arrive at a restaurant and discover that the food is worse than you remembered. In the weather example, you go for a walk and unexpectedly get rained on. Why is the first example about reliability and the second example about volatility?

We thank Reviewer 3 for pointing out this source of confusion. We agree that the distinction between unreliability and volatility could be articulated more clearly, and we have revised the text and task descriptions accordingly. In particular, we have added a more concrete example of volatility and clarified the temporal nature of the distinction. The revised section now reads:

"In these scenarios, stochasticity diminishes as the future approaches and it is often possible to change course as the future becomes more certain. For example, when crafting an itinerary for a trip, one might choose to plan specific outdoor activities only when the weather forecast becomes less volatile. In our next task variant, we examined how this temporal fluctuation, or volatility, influences planning effort."

The key distinction between the two forms of stochasticity is *temporal*. In the *unreliable* case (e.g., outdated restaurant recommendations), uncertainty is constant: outcomes are noisy, but that noise does not change over time and cannot be reduced by waiting or observing. In the *volatile* case (e.g., an evolving weather forecast), uncertainty itself changes over time—the further into the future one looks, the more uncertain the outcome. Crucially, volatility often allows for *course correction*: as time passes and the world evolves, agents can update their beliefs and adjust their plans accordingly.

In the context of the task, the distinction is also not that clear. In the unreliable case, you choose a chest and instead get a random reward. In the volatile case, you choose a path and instead get random future rewards. The only difference is that in the unreliable case you experience the random reward immediately, while in the volatile case you have to wait one or more turns to collect, or avoid, the random reward. So what? Why is that difference important?

We appreciate this question, as it allowed us to clarify both the computational and behavioral implications of volatility in our task. While the two conditions may appear superficially similar, there are two critical differences that make volatility a distinct and consequential form of uncertainty for planning.

First, in the volatility condition, outcomes evolve dynamically as the agent moves forward. Each treasure chest below the current row has a probability p of being resampled on every turn, meaning that by the time the participant reaches row r, the chest values there could have been resampled up to $r-1$ times. The effective stochasticity at depth r therefore compounds approximately as p^{r-1}, creating qualitatively different planning challenges than in the reliability condition, where stochasticity is constant and does not accumulate over time.

Second, in the volatility condition, the displayed values are always *truthful*—they represent the chest's current value, even though that value may change in subsequent turns. This allows participants to update and replan dynamically as they descend the board. In contrast, in the reliability condition, the display itself is noisy: the participant cannot "wait out" the uncertainty, as it reflects inherent outcome unreliability rather than time-varying volatility.

3.4.2 Differences Between Stochasticity Conditions

Setting those issues aside, I still find the conclusions underwhelming. I was expecting to see something tying the different stochasticity manipulations together. Instead, we just learn that every manipulation leads to less depth of planning with increasing stochasticity. But how should we interpret the coefficients? Is this the ranking that we would expect? It seems that some manipulations might be worse than others. For instance, the reliability manipulation only affects a single reward at a time, while the volatility manipulation affects multiple rewards at a time. So, you might expect volatility to have a larger effect on

behavior than reliability. That does not seem to be the case. In any case, it seems important to include an analysis of the benefit of planning ahead in these different stochastic settings. That would really help improve the contribution here.

We appreciate the reviewer's suggestion to directly compare the stochasticity manipulations. Consistent with the reviewer's intuition, we expected that volatility, by compounding uncertainty across future steps, might reduce the marginal benefit of planning more sharply than reliability, which introduces uncertainty uniformly at each step.

To test this hypothesis, we conducted resource–rational simulations (Supplementary Section S3) examining how optimal planning sensitivity ($\log \beta$) should vary across conditions when agents trade off expected reward against cognitive effort. These simulations show that volatility changes the reward–effort landscape. As predicted, increasing β yields diminishing returns more quickly under volatility than under reliability:

> "First, for a fixed β, as stochasticity q increases, the expected reward $R(\beta, q)$ decreases. Second, for a fixed q, increasing β improves performance but saturates eventually, reflecting diminishing marginal returns. The rate of saturation differs across conditions: in the volatility condition, rewards saturate earlier than in the reliability condition, reflecting compounding stochasticity across planning steps."

However, when translating reward landscapes into optimal effort choices, the simulations did not show robust qualitative differences between stochasticity conditions. Across both approaches, the dominant pattern was a shared monotonic decrease in planning sensitivity with increasing stochasticity. Any small differences that occasionally appeared were not consistent across runs.

Thus, while the stochasticity manipulations differ in how they structure the reward–effort tradeoff, both the behavioral data and normative analysis converge on the same conclusion: across reliability, volatility, and controllability, increasing stochasticity leads to reduced planning effort. This uniform adaptation supports a generalizable principle linking environmental uncertainty to effort allocation in planning.

3.5 Results and Alternatives

The behavioral results hypotheses/results strike me as quite obvious and would seemingly result from any model of costly cognitive effort. If your decisions have less impact on your outcomes, you should exert less effort in making the correct decisions. There are countless models and papers on this idea. What does the current paper add to the discussion? ... In my view the only notable finding in the paper is that the depth of search varies with stochasticity. But again, what's a plausible alternative?

We appreciate the reviewer's perspective and the opportunity to clarify the contribution. We agree that the intuition that effort should scale with the value of control is broadly consistent with resource-rational principles. However, this effect has not previously been demonstrated in the context of multi-step planning under environmental stochasticity, nor has prior work identified the mechanism by which planning effort changes in such settings. Our revisions directly address this gap.

As mentioned earlier, our resource rationality simulations show that the optimal level of planning sensitivity ($\log \beta$) decreases systematically with stochasticity. Crucially, this reproduces both the direction and shape of our empirical effects, linking behavior to a principled reward–effort trade-off rather than a post hoc explanation.

The reviewer asks specifically about plausible alternatives. Our expanded modeling pipeline directly tested filter adaptation and policy compression as possible mechanisms for allocating effort. We found that policy compression was a better fit to the participant behavior. This result is not trivial: one could expect depth to shorten (as we did in our first draft), remain constant (if constrained by working memory or attention limits), or even increase (to counteract uncertainty). We find that participants hold planning depth constant and selectively reduce value sensitivity as stochasticity increases.

Our findings reveal an additional novel phenomenon: participants consistently rely on determinizing heuristics when planning, even where computing expected values is tractable. To our knowledge, this is the first systematic demonstration of determinization as a planning mechanism in human behavior.

Finally, we acknowledge that resource rationality provides a high-level principle, echoing the reviewer's point that "if your decisions have less impact on your outcomes, you should exert less effort". Our contribution lies in specifying and testing concrete mechanisms under that principle, and showing that: (1) Planning effort decreases with stochasticity across three environment types (2) the mechanism is policy compression rather than depth reduction (or other forms of filter adaptation) (3) determinization plays a key role in this adjustment (4) these behavioral effects qualitatively match the findings of resource-rational simulations.

Taken together, our behavioral analyses, expanded model suite, and normative simulations advance the literature by moving beyond the intuition that people expend less effort under uncertainty. We provide a mechanistic account of how planning is simplified under stochasticity and demonstrate that both the empirical pattern and its computational form align with resource rational principles.

3.6 Minor Comments

3.6.1 Instruction Details

> Please include details of the instructions, practice trials, and comprehension checks. These details are important to evaluate how participants likely came up with strategies for this task.

We have implemented the reviewer's suggestion and have now added a new section to the Supplement with a detailed account of all instructions, practice trials, and comprehension checks (Supplementary Section S6).

3.6.2 Clarification of Planning Animation

> How could participants continue planning/deciding while waiting for the animation to resolve? While that might be possible in the reliability case, it doesn't seem possible in the other cases.

We apologize for the confusion. What we meant is that participants were not withheld from *acting* while an animation was resolving. We have clarified this in the main text:

> "Importantly, participants were not required to wait until the animation completed before making their next choice, allowing them to continue acting without waiting for visual feedback if they chose to do so."

Our main concern was that forcing participants to wait could have led to frustration or altered planning behavior. By allowing them to act at their own pace, we ensured that decision-making remained self-directed. Additionally, we matched the timing of the animations for shaking and opening treasure chests so that participants would not prefer one over the other based on the duration of outcome resolution.

References

[1] Huys, Q. J. M. *et al.* Bonsai Trees in Your Head: How the Pavlovian System Sculpts Goal-Directed Choices by Pruning Decision Trees. *PLOS Computational Biology* **8**, e1002410 (2012). URL https://journals.plos.org/ploscompbiol/article?id=10.1371/journal.pcbi.1002410. Publisher: Public Library of Science.

[2] Sezener, C. E., Dezfouli, A. & Keramati, M. Optimizing the depth and the direction of prospective planning using information values. *PLOS Computational Biology* **15**, e1006827 (2019). URL https://journals.plos.org/ploscompbiol/article?id=10.1371/journal.pcbi.1006827. Publisher: Public Library of Science.

[3] Van Opheusden, B. *et al.* Expertise increases planning depth in human gameplay. *Nature* **618**, 1000–1005 (2023). URL https://www.nature.com/articles/s41586-023-06124-2. Number: 7967 Publisher: Nature Publishing Group.

[4] Ortega, P. A. & Braun, D. A. Thermodynamics as a theory of decision-making with information-processing costs. *Proceedings of the Royal Society A: Mathematical, Physical and Engineering Sciences* **469**, 20120683 (2013). URL https://royalsocietypublishing.org/doi/full/10.1098/rspa.2012.0683. Publisher: Royal Society.

[5] Bhui, R., Lai, L. & Gershman, S. J. Resource-rational decision making. *Current Opinion in Behavioral Sciences* **41**, 15–21 (2021). URL https://linkinghub.elsevier.com/retrieve/pii/S2352154621000371.

[6] Daw, N. D., Niv, Y. & Dayan, P. Uncertainty-based competition between prefrontal and dorsolateral striatal systems for behavioral control. *Nature neuroscience* **8**, 1704–1711 (2005). ISBN: 1097-6256 Publisher: Nature Publishing Group US New York.

[7] Kool, W., Cushman, F. A. & Gershman, S. J. When Does Model-Based Control Pay Off? *PLOS Computational Biology* **12**, e1005090 (2016). URL https://dx.plos.org/10.1371/journal.pcbi.1005090.

[8] Lai, L. & Gershman, S. J. in *Chapter Five - Policy compression: An information bottleneck in action selection* (ed.Federmeier, K. D.) *Psychology of Learning and Motivation*, Vol. 74 of *The Psychology of Learning and Motivation* 195–232 (Academic Press, 2021). URL https://www.sciencedirect.com/science/article/pii/S0079742121000049.

[9] Rigoux, L., Stephan, K. E., Friston, K. J. & Daunizeau, J. Bayesian model selection for group studies - revisited. *NeuroImage* **84**, 971–985 (2014).

[10] Barr, D. J., Levy, R., Scheepers, C. & Tily, H. J. Random effects structure for confirmatory hypothesis testing: Keep it maximal. *Journal of Memory and Language* **68**, 255–278 (2013). URL https://linkinghub.elsevier.com/retrieve/pii/

S0749596X12001180.

[11] Lai, L. & Gershman, S. J. Human decision making balances reward maximization and policy compression. *PLOS Computational Biology* **20**, e1012057 (2024). URL https://journals.plos.org/ploscompbiol/article?id=10.1371/journal.pcbi.1012057. Publisher: Public Library of Science.

[12] Gershman, S. J. & Lak, A. Policy Complexity Suppresses Dopamine Responses. *The Journal of Neuroscience* **45**, e1756242024 (2025). URL https://www.jneurosci.org/lookup/doi/10.1523/JNEUROSCI.1756-24.2024.

[13] Akam, T., Costa, R. & Dayan, P. Simple Plans or Sophisticated Habits? State, Transition and Learning Interactions in the Two-Step Task. *PLOS Computational Biology* **11**, e1004648 (2015). URL https://dx.plos.org/10.1371/journal.pcbi.1004648.

[14] Eysenbach, B., Salakhutdinov, R. & Levine, S. Robust Predictable Control (2021). URL http://arxiv.org/abs/2109.03214. ArXiv:2109.03214 [cs].

Overview of Key Revisions

We thank the reviewers for their suggestions, comments, and feedback. In response, we have revised our pipeline to include more robust and methodologically rigorous analysis and tightened our main text to reflect these changes. Below we summarize the main methodological and interpretational changes introduced in this revision.

Improved methodological robustness and verification of recovery

The largest substantive change in this revision cycle was switching our goodness-of-fit metric from NLL / AIC / BIC to cross-validated negative log likelihood, a key methodological improvement. In earlier versions, we used Δ NLL (in our case, equivalent to Δ AIC / BIC because all models had the same number of parameters) as our way of distinguishing the best-fitting model from the alternatives.

This change was motivated by R3's suggestion to extend model recovery across all model classes. In doing so, we found that raw NLL was poorly suited to recover some of our models given the model structure. Specifically, while all models share the same number of parameters, some parameter values disproportionately affect certain models' behavior. For example, the models with Max value function were relatively insensitive to the rank filter parameter k compared to models with other value functions (e.g. Path). As such, we chose a more robust metric by using 5-fold cross-validation and using the sum of the held-out NLLs as the final metric to capture out-of-sample predictive accuracy. Upon changing our pipeline we found that all models were recoverable and that our main findings remained unchanged. The only substantive change was observed instability in the secondary RFX-BMC analysis, which we discuss in the Results and Supplement.

Clarifications

In light of reviewer comments, we made several clarifications in the main text regarding conceptual assumptions and theoretical connections:

- **Consistency with preregistration.** Where appropriate, in the main text we have labeled additional analysis (RT, RFX-BMC) as exploratory and have clarified our deviation from preregistered analyses (using CV-NLL instead of AIC / BIC, using LMMs instead of ANOVA).
- **Model heterogeneity.** We clarify the possibility of model heterogeneity both across and within subjects in the Discussion and document our preliminary RFX-BMC analysis in the Supplement.
- **Policy compression.** We address how we operationalize policy compression in our particular case and explicitly mention the lack of a marginal / default policy and perseveration term in the main text Modeling section.
- **Resource-rational strategy selection.** We discuss the possibility of resource rational strategies that do not utilize the expected-value calculation and how they may arise from resource rational strategy selection in the Discussion.

1 Reviewer 1 Comments

1.1 Policy compression and perseveration term

The authors have done a good job addressing my comments. The only remaining criticism I have (unrelated to my original comments but related to some of the new content) is that what they are calling "policy compression" isn't strictly speaking what that term refers to. They model changes in value sensitivity, which is indeed one aspect of policy compression. But another aspect is absent: the marginal policy (perseveration) term, which is needed to connect the model to compression in the information-theoretic sense. To be clear, I'm not suggesting that the authors have to add this term (though of course they could try that); I just think there needs to be a caveat somewhere that clarifies this point. The substantive reason it's important is that the marginal policy term is what separates policy compression from some form of generic value insensitivity, noise, or explore/exploit mechanism.

We thank the reviewer for this thoughtful clarification and agree that our original framing did not sufficiently distinguish between our implementation and fuller information-theoretic formulations of policy compression. In our models, we operationalize policy compression through adaptive changes in policy sensitivity (inverse temperature), which we view as one way in which effective policy complexity may be reduced under stochastic environments.

We retained the term "policy compression" because the formulation was motivated by that literature and because reductions in policy sensitivity provide a principled mechanism through which policies may become compressed or simplified under increasing uncertainty. Our use of the term "policy compression" follows broader formulations that define compression as the reduction of policy complexity under computational constraints, often operationalized as reduced sensitivity to state distinctions or reduced policy precision.

At the same time, we agree that our implementation does not include an explicit marginal or "default" policy term, which plays an important role in some canonical information-theoretic formulations of policy compression and rational inattention by regularizing policies relative to prior action tendencies. Such terms can additionally give rise to perseveration-like effects that we do not explicitly model in our present study, but consider an interesting direction in future work.

As such, we have added an explicit clarification point in our model specification in the main text describing the particular operationalization of policy compression adopted in the present work:

> We operationalize policy compression as adaptive changes in policy sensitivity (inverse temperature), abstracting away additional effects of explicit policy priors (e.g., perseveration or a default reference policy).

2 Reviewer 2 Comments

2.1 Model heterogeneity across and within subjects

> The authors thoroughly addressed all my comments, and I find the revised manuscript substantially stronger. The only remaining issue is that, to my understanding, the new random-effects Bayesian model selection procedure allows heterogeneity across but not within individuals, in the sense of an individual combining multiple heuristics. If my understanding is correct, I suggest that the authors at least note in the main text the possibility that participants used hybrid strategies.

We thank the reviewer for this comment. We have added a section dedicated to the possibility of hybrid strategies to the Discussion section of our main text:

> One open direction involves the study of individual differences in strategy selection and adaptation to stochasticity. The connection to resource rationality suggests that participants may themselves engage in multiple levels of strategy simplification, a phenomenon that may be better characterized at the individual level with richer datasets or adaptive designs. Our current experimental and computational methods do not capture within-individual mixing of strategies; although hybrid strategy use remains plausible, distinguishing such models would likely require task designs and computational frameworks explicitly constructed to dissociate dynamic strategy switching at an individual level.

Additionally, we observed variability in the results of the RFX-BMC analysis (a thorough discussion of the robustness analysis can be found in the Supplement). For this reason, as well as the fact that our study focuses primarily on aggregate level modeling of participant behavior, we concluded that conducting finer-grained analysis within and across individuals would require the design of different experiments and computational methodologies not present in this paper

> To assess participant-level heterogeneity in strategy selection, we conducted an exploratory random-effects Bayesian model comparison (RFX-BMC; Supplementary Section S7). Results were not robust enough to support strong quantitative claims about individual differences in strategy use. We note that RFX-BMC captures heterogeneity across but not within individuals, and therefore does not speak to the possibility of hybrid or mixed strategies within a single participant.

2.2 Consistency with preregistration

> The hypothesis and experimental design in the preregistration and manuscript are consistent. The analysis is also largely consistent. There are two minor changes: the method of model comparison has been changed (AIC/BIC/bootstrap in the prereg), and the method of measuring effects across stochasticity levels (ANOVA in the prereg) - in my view these changes merely need some justification. Additionally, there are some extra analyses added whether due to the authors or reviewer requests (e.g. reaction time analyses) that should be labeled exploratory.

We thank the reviewer for this point and for ensuring our analyses are consistent with our preregistered intent. In this revision we have switched to cross-validated negative log likelihood as a measure of goodness of fit, driven by a desire to be more methodologically rigorous and to capture out-of-sample predictive accuracy. Here, we

have made an effort to clarify *why* we use this metric instead of AIC / BIC in the Methods section of our main text:

> Our choice of 5-fold cross-validation over AIC / BIC as suggested in our pre-registration reflects the fact that (1) some parameters disproportionately affect the behavior of particular model subclasses more than others, and (2) we care about the out-of-sample predictive accuracy of our models when inspecting model fit. For our across-model analysis of goodness-of-fit and model comparison, we used 5-fold CV NLLs, consistent with best practices. For our within-model analysis (i.e. extracting winning parameter values for a given model), we re-ran the full fitting procedure for the model on the entire dataset to extract the parameter set that achieved the lowest negative-log-likelihood.

Similarly, we have added a section to clarify why we have used GLMMs and LMMs in place of ANOVA:

> Our choice to use GLMMs and LMMs in place of ANOVA (as suggested in our preregistration) reflects our interest in treating stochasticity as a continuous variable varying along a spectrum, rather than solely as a set of discrete experimental conditions.

We also added explicit clarification for exploratory analyses for different parts of the main text and supplement, for the response time analysis:

> To assess planning effort behaviorally, we conducted exploratory analyses of response times as an empirical proxy for planning effort.

as well as for the RFX-BMC analysis suggested by a prior reviewer comment:

> To assess participant-level heterogeneity in strategy selection, we conducted an exploratory random-effects Bayesian model comparison (RFX-BMC; Supplementary Section S7).

3 Reviewer 3 Comments

The authors have largely addressed my concerns and I think that the article is much improved. I just have a few small lingering concerns.

(1) It isn't obvious, at least to me, that the resource rationality necessarily means using the expected value strategy. It seems like another approach could yield higher expected gains for the same amount of effort. It would be helpful if the authors could elaborate on this point and clarify why they think that expected value maximization is always optimal.

We thank the reviewer for this helpful clarification. We agree that our original framing of resource rationality was somewhat narrow, as it focused on how people might adapt within a given model class rather than also considering how the choice of model class itself could be constrained by computation. In our initial framing, we effectively assumed that participants start from an optimal planning strategy and then adjust its parameters (such as policy sensitivity) in a resource-rational way. As the reviewer points out, it is possible that resource constraints also shape the strategy selection process itself, with people first selecting a tractable approximation to optimal planning and then optimizing within that approximation. We now clarify this distinction in the main text and soften our claim that resource-rationality uniquely motivates an expected-value baseline:

> An additional level of resource rationality may also operate in the selection of the model itself. In principle, an unconstrained optimal agent could compute expected future rewards directly. Under realistic computational constraints, however, resource-aware agents may instead favor simpler "good-enough" approximations that achieve favorable reward-effort tradeoffs. From this perspective, participants may first select a tractable planning strategy and then adapt the parameters of that strategy to different levels of stochasticity. Consistent with this interpretation, the best-fitting models in our study relied on simpler determinizing heuristics that treated the environment as effectively deterministic, even in settings where explicit expected-value computations remained computationally tractable in principle.

3.1 Model recovery

(2) I appreciate the model recovery exercise, but I think it is incomplete. We also need to know whether other models are correctly recovered when they are the true data generating process. Otherwise it is possible that other data generating processes could be mistaken as evidence for PC Depth-filtering Level-Mean or PC Depth-filtering Path.

The reviewer raises an important point, and we agree that a full model recovery analysis should include testing whether each candidate model can be recovered when it is the true data-generating process. We have therefore extended our recovery analysis to include all candidate models in the model space and made note of our results in the Supplement.

In conducting this analysis, we also identified an issue with using NLL/AIC/BIC as the goodness-of-fit metric in this setting. Specifically, we found that some models were differentially sensitive to parameterization of the filtering component, leading to asymmetries in how parameter changes translated into behavioral predictions across model classes. As a result, models with the same nominal number of parameters could

still differ in their effective flexibility. To address this, we adopted cross-validated negative log likelihood as our primary model comparison metric, which is more robust to these asymmetries. We clarify this choice in the main text.

Using this updated procedure, we find that all models are recoverable: when each model is used to generate synthetic data, our fitting and comparison procedure correctly identifies the generating model as the best-fitting account in every case. We have added a full recovery analysis for all 30 model variants in the Supplement.

> We then fitted the synthetic data using our model fitting procedure to see whether the ground truth models were recoverable. Consistent with our model fitting procedure for our real data, we conducted five-fold cross-validation to establish the best-fitting model for each synthetic dataset, using the cross-validated negative log likelihood (sum of held-out fold NLLs) as our measure of goodness-of-fit. A model was said to be recoverable when bootstrap distributions of ΔCV-NLL (each alternative minus the generating baseline, with $n = 10^6$ participant resamples) showed that the generating model was substantially favored (using 95% confidence intervals) relative to all alternatives.
>
> We find that under all conditions, for *all* models, the best-fitting model corresponded with the model that actually generated the synthetic dataset, demonstrating that our models are both distinguishable and recoverable from simulated data.

One downstream consequence of this change was occasional instability in our secondary RFX-BMC analysis across different fold instantiations (importantly, note that we did *not* observe such instability in our primary model comparison analysis). This reflects the fact that in our main result, the cross-validated negative log likelihood showed that several members of the winning class of models (PC depth) were mutually competitive even at the population level. We document our analysis and results for this secondary analysis in the Supplement and updated our Results section to reflect these changes:

> To assess participant-level heterogeneity in strategy selection, we conducted an exploratory random-effects Bayesian model comparison (RFX-BMC; Supplementary Section S7). Results were not robust enough to support strong quantitative claims about individual differences in strategy use. We note that RFX-BMC captures heterogeneity across but not within individuals, and therefore does not speak to the possibility of hybrid or mixed strategies within a single participant.
